# The *C. elegans* embryonic transcriptome with tissue, time, and alternative splicing resolution

**DOI:** 10.1101/gr.243394.118

**Published:** 2019-06

**Authors:** Adam D. Warner, Louis Gevirtzman, LaDeana W. Hillier, Brent Ewing, Robert H. Waterston

**Affiliations:** Department of Genome Sciences, School of Medicine, University of Washington, Seattle, Washington 98195, USA

## Abstract

We have used RNA-seq in *Caenorhabditis elegans* to produce transcription profiles for seven specific embryonic cell populations from gastrulation to the onset of terminal differentiation. The expression data for these seven cell populations, covering major cell lineages and tissues in the worm*,* reveal the complex and dynamic changes in gene expression, both spatially and temporally. Also, within genes, start sites and exon usage can be highly differential, producing transcripts that are specific to developmental periods or cell lineages. We have also found evidence of novel exons and introns, as well as differential usage of SL1 and SL2 splice leaders. By combining this data set with the modERN ChIP-seq resource, we are able to support and predict gene regulatory relationships. The detailed information on differences and similarities between gene expression in cell lineages and tissues should be of great value to the community and provides a framework for the investigation of expression in individual cells.

As the *Caenorhabditis elegans* embryo develops from one cell to 558 cells, the transcriptional profile of each cell undergoes unique changes to drive differentiation into distinct cell types and tissues. Although the invariant lineage of the cell divisions has been mapped down to each division and apoptotic event (Sulston et al. 1983), we do not yet have a detailed map of the underlying changes in mRNA that are required for this distinct programmed cell differentiation. Gene expression has been measured in isolated cell types (Meissner et al. 2009; Spencer et al. 2011; Burdick et al. 2016); however, the data were primarily generated from either mixed populations or loosely synchronized single-time-point samples, giving a valuable but limited snapshot of transcription. Many of these previous studies have relied primarily on techniques such as serial analysis of gene expression (SAGE) and microarray to assay transcription, which have drawbacks compared with RNA-seq (Zhao et al. 2014). Using RNA-seq, a whole-embryo time series was produced (Boeck et al. 2016), which provides temporal data from the early embryo to late in embryonic development, but the data set lacks cell-type–specificity. Expression data sets with single-cell resolution (Hashimshony et al. 2012, 2015; Tintori et al. 2016; Cao et al. 2017) are promising in terms of refined temporal and spatial information but suffer from sparse sampling of the transcripts per cell. Many single-cell RNA-seq methods also rely on the end sequencing of cDNA molecules and thus largely fail to capture alternative splicing events.

To address the need for a more comprehensive mRNA data set that captures the transcripts in specific tissues during embryonic development, we have used fluorescence-activated cell sorting (FACS) to isolate fluorescent cells at multiple time points from synchronized embryos containing early and specific tissue/lineage markers (Murray et al. 2012). We observe differences in gene expression levels both between cell types and over time within the same population. Furthermore, we observe differential promoter and differential exon usage that leads to additional differences between cell types. To show the utility of our data set, we have focused on a set of 130 genes partially outlined by Etheridge et al. (2015), which are homologs of the more than 150 genes forming the highly conserved network of genes that comprise the integrin adhesome in mammalian species.

## Results

### Production of cell-type–specific transcriptome data sets as a time series

To obtain transcription profiles of the major tissues and organs of *C. elegans*, we exploited strains expressing fluorescent reporters in muscle (and coelomocytes; *hlh-1p::mCherry*), intestine (*end-1p::mCherry*), neurons (*cnd-1p::mCherry*), pharynx (*pha-4::GFP*), and hypodermis (*nhr-25::GFP*) (Murray et al. 2012). Because the latter two reporters are also expressed in the intestine, we used a doubly marked strain to exclude intestine (*end-1p::mCherry*). The lineages in which these genes are expressed, as determined by 4D microscopy, include most of the cells of the animal with the exception of the D and P4 lineages (Supplemental Fig. S1; Murray et al. 2012). The D lineage only produces body wall muscle, and the genes expressed there are likely to be overlapping with other *hlh-1*-labeled cells. P4 exclusively produces the germline precursors, whose development occurs largely post-embryonically. In addition to the tissue/organ samples, we used markers to recover descendants of ABa (*tbx-37p::mCherry*) and ABala (*ceh-32p::mCherry*). ABa produces a variety of neurons, pharynx, and hypodermis, whereas ABala produces a subset of neurons and glial cells (*ceh-32* also marks the ABarpp lineage, which gives rise to a subset of hypodermal cells, glial cells, and neurons) (Supplemental Fig. S1). To assay gene expression in each of these tissues over time, we sampled synchronized populations of embryos at 90-min intervals for five time points (Supplemental Fig. S2). Our initial time point was ∼120 min after embryo isolation, shortly after the fluorescent markers were first visible and about halfway through gastrulation. The last time point (∼480 min after isolation) was approximately when the embryo reached the threefold stage, when most cells are beginning terminal differentiation, but before the cuticle forms; when this outer layer is present, it complicates cell recovery. For each sample, we also collected a set of unlabeled cells. To obtain well-synchronized populations, we harvested young adult animals from a synchronized population just as the first eggs were appearing (for details, see Methods). At the initial time point, the bulk of these embryos had between eight and 26 cells, with 15-cell embryos being the most abundant (Supplemental Fig. S3); this synchrony persisted through successive time points (Supplemental Fig. S4). To obtain time- and tissue-specific exon usage, we sampled the full length of both nonpolyadenylated and polyadenylated transcripts (see Methods). Lastly, we devised a novel PCR duplicate detection method to identify the fraction of PCR duplicates and remove them from our sequencing output without distorting the expression values of highly expressed genes. Our method estimates the PCR duplicate rate in low-coverage genome regions where duplicates would be rare by chance and removes these duplicate reads as well as proportional numbers of detected duplicate read pairs in high-coverage regions (for details, see Supplemental Methods).

In total, we produced time series data for five different tissue/organ-specific cell populations and two lineage-specific populations, each of which is henceforth described as a tissue for the sake of simplicity. Each time series had two biological replicates, except for the ABa and muscle series, which had three and four replicates, respectively. After removal of rRNA and PCR duplicate reads, our 85 samples had a total of more than 1 billion paired-end reads, giving an average of 12 million read pairs mapped to the transcriptome for each sample and a minimum of 4 million read pairs. With this depth of coverage and approximately 20,000 mRNA molecules per post-mitotic cell (Boeck et al. 2016), each mRNA molecule should have been sampled at least once on average, even in the most cellularly complex sample (the *cnd-1* samples, which selected about 250 cells from the embryo). For each sample, expression levels were calculated (Glaus et al. 2012) in transcripts per million (TPM), and we confirmed quality of the replicates using Spearman's correlation and principal component analysis plots (Supplemental Figs. S5, S6).

As we noted with whole-animal rRNA-subtracted samples (Boeck et al. 2016), reads derived from nonpolyadenylated histone messages were abundant in the early samples, particularly for the rapidly dividing neuronal lineages. In this study, the histones accounted for 35%–43% of the total reads in early ABala samples (Supplemental Fig. S7). This high fraction of histone transcripts makes clear the demands that the rapid division rates place on the *C. elegans* embryo. Nevertheless, because the representation of histone transcripts and rRNA content vary considerably between tissues and time points and because they are not included in other studies that use poly(A)-selected transcripts, we excluded reads mapping to histones (and rRNA) in both the calculation of the TPM and the remainder of the analysis.

In aggregate, we found evidence for expression of 18,292 protein coding genes (TPM ≥ 1 in at least one sample) and robust expression for 11,408 genes (TPM ≥ 15 in at least one sample) (Supplemental Fig. S8). Our data confirmed 93,164 WormBase (WS245)-annotated introns and also confirmed 14,017 introns not annotated in WormBase WS245 that we detected previously (Boeck et al. 2016). An additional 1553 previously unannotated introns were identified; either these fall within a transcript and represent an intron missing from the gene model or they fall within 500 bases of an annotated WormBase transcript and could represent extensions of existing models. Finally, 2579 additional introns were identified that were more than 500 bases outside of annotated transcripts or spanned multiple WS245 transcript boundaries and could represent as yet unannotated genes.

### Dynamics of gene expression

To evaluate the dynamics of gene expression between tissues and over time, we compared expression among all pairwise combinations of the samples using DESeq2 (Love et al. 2014). In particular, we were interested in genes that changed expression significantly between tissues at the same time point or between time points within the same tissue. Within each series, the number of differentially expressed (DE) genes increases over time, reflecting the implementation of differentiation programming (Supplemental Fig. S9). Associated with this increase of DE genes, we note a shift in the distribution of expressed genes over time, with an increase of about 1000 in the number of modestly expressed genes (TPM > 6 and < 100) and a corresponding drop in nonexpressed or poorly expressed genes (TPM < 6) (Supplemental Fig. S10). In comparing the different tissues at similar time points, the intestinal samples consistently have the most DE genes, followed by muscle and neurons. As expected, the two lineage samples show fewer DE genes compared against tissues with which they share cells (Supplemental Fig. S11).

To find genes specifically enriched in a single series at particular times, we used DESeq2 to identify genes with a log base twofold change (log_2_FC) value ≥1.0 for the highest-expressed tissue over the second highest-expressed tissue and an adjusted *P*-value of ≤0.1, while excluding samples that shared common cells (see Methods; Supplemental Table S1). In total, 2700 genes were scored as DE in a single tissue (Table 1; for full data, see Supplemental Table S2a; for summary, see Supplemental Table S2b). The intestinal and muscle series have the highest number of DE genes with 1182 and 694 genes, respectively. These DE genes fall into the types of Gene Ontology categories expected for the cell types present in the samples (Supplemental File S1).

**Table 1.**
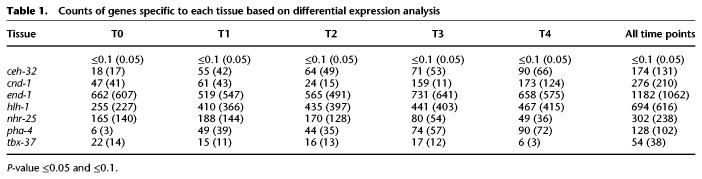
Counts of genes specific to each tissue based on differential expression analysis

**Figure 1.**
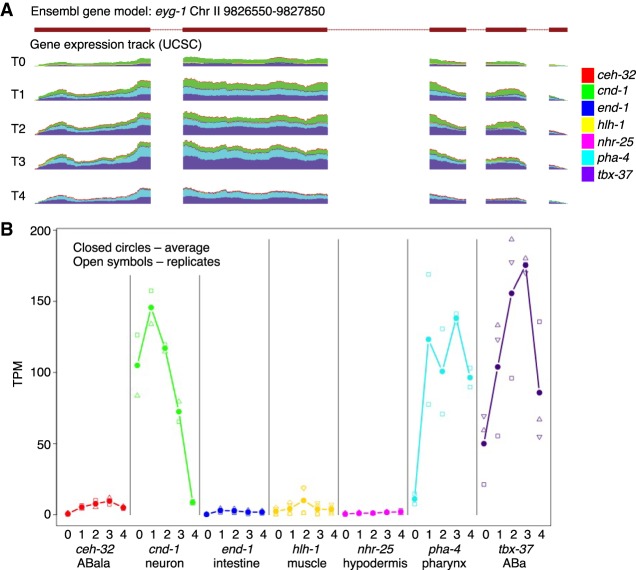
Gene expression changes spatially over time. In gene *eyg-1*, expression transitions from being expressed primarily in neurons at time point 0 to being expressed almost completely in pharyngeal cells by time point 4 (*A*). Significant expression occurs through all time points in ABa lineage, which encompasses a subset of both neuronal and pharyngeal cell types. (*B*) Average expression values for *eyg-1* are labeled as closed circles and are connected; individual data points are shown as open symbols. Legend indicates colors for each tissue. Scale is zero to 850 reads for each time point (normalized to 20 million total reads per sample; *A*).

An additional 54 genes showed differential expression for one series at one time point and a different series at a later time point in development, thus changing the tissue specificity over time. Often these appeared to represent earlier onset of expression in one tissue of a zygotically expressed gene, and the pattern is seen across replicates (Fig. 1; Supplemental Table S3). We also looked for genes whose expression varied minimally over time within each series and found 1572 genes that are stably expressed in one or more tissues over time (Supplemental Table S4).

**Figure 2.**
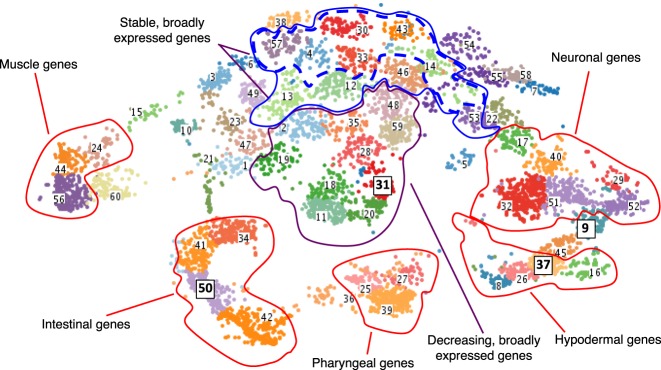
Clustered gene expression visualized with t-SNE. Gene expression values (TPM) were clustered using fuzzy *k*-means clustering and then visualized in t-SNE space to group genes with similar expression patterns. Genes that have enriched expression based on tissue are clustered outward from nonenriched genes in the plot and separated based on their specific tissue enrichment (outlined in red). Within nontissue enriched genes, the separation of genes into clusters is often temporal in nature, with stably expressed genes appearing in clusters toward the *top* of the plot (blue outline) and broadly expressed genes that are dropping in expression value located in the very *center* of the plot (purple outline). Clusters with the highest proportion of stable and broadly expressed genes are outlined in a dashed blue line. Clusters discussed in more detail in the text and in Figure 3 are highlighted with boxes around the label and larger font size.

To identify more complicated spatiotemporal relationships among genes, we used fuzzy *k*-means clustering (Karayiannis and Bezdek 1997) to cluster the 11,408 robustly expressed genes (≥15 TPM in at least one sample) into 60 clusters in 35-dimensional space (seven cell types and five time points in each) (Supplemental Table S5). To visualize the clusters, t-SNE (van der Maaten 2014) was used to reduce the dimensions, where each point is a gene in t-SNE space (Fig. 2).

**Figure 3.**
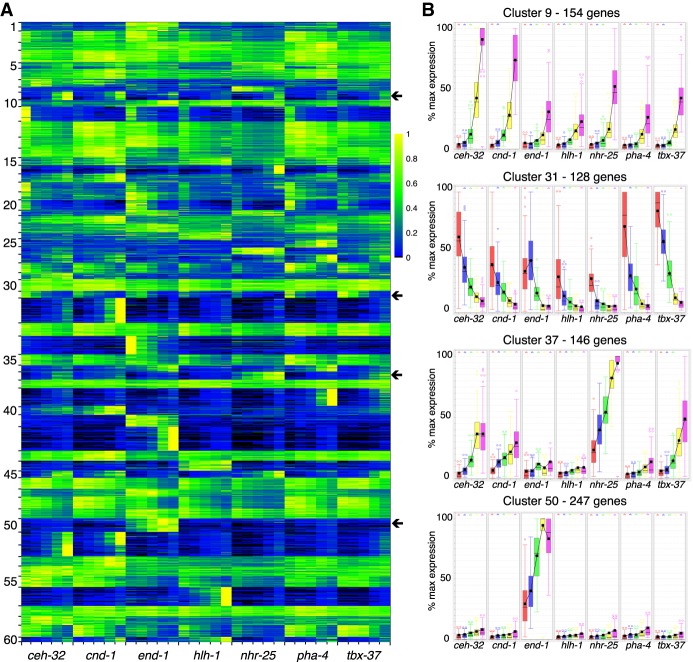
Gene expression across all tissues and stages. (*A*) By using the fuzzy *k*-means clustering displayed in Figure 2, expression was normalized to a maximum of one for the highest-expressed sample and then displayed as a heatmap. Each row is a gene, with the rows organized as clusters. Cluster numbers are given on the *left*. Within a cluster of gene expression, both temporal changes in gene expression and differential gene expression based on tissue are observed. (*B*) Examples of the different patterns of gene expression that occur within the clusters indicated by the arrows. There is broad, increasing zygotic expression in cluster 9, broad dropping expression in cluster 31, zygotic neuronal and hypodermal expression in cluster 37, and rapidly rising intestinal-specific expression in cluster 50.

To examine the expression patterns of genes in the clusters, we generated a heatmap and box-and-whisker plots of the expression of the clustered genes across the 35 different tissue/time points (Fig. 3). A variety of distinct patterns is evident, and nearby clusters have related patterns. For example, cluster 50 is composed of genes with increasing expression specifically in the intestinal (*end-1*) samples. Adjacent clusters have enriched intestinal expression but with different temporal patterns. Other groups of clusters have genes enriched in muscle, pharynx, hypodermis, and neuronal cells as indicated (Fig. 2). In total, 4370 genes of the 11,408 genes analyzed are associated with a tissue-specific cluster with the parameters used.

Some clusters contain genes expressed in more than one, but not all, tissues. For example, clusters 45 and 9 lie between the groups of clusters for the hypodermis and neurons (Fig. 2), and genes within clusters 45 and 9 have enriched expression in both the hypodermis and neurons. Similarly, cluster 36 lies between the groups of gene clusters for the pharynx and intestine and contains genes that are primarily expressed in both the pharynx and intestine.

Among genes that are more broadly expressed, we see temporal patterns. Genes in cluster 31 have decreasing expression across all tissues and represent maternally expressed genes. The labeled adjacent clusters also have genes that are decreasing in expression over time with fairly uniform expression among all tissues. Conversely, >70% of the genes in clusters 4, 14, 30, 33, 43, and 57 are expressed stably in every tissue type. Adjacent clusters 12, 13, 46, and 53 meet these criteria for 40% of their genes. A high proportion of the broadly and stably expressed genes are in operons: 913 (58%) genes in operons are among the 1572 stably expressed genes compared with 3128 genes in operons in the 11,408 genes analyzed (27%; chi-squared test *P*-value <0.00001, significant at *P* < 0.01).

The integrin adhesome provides an example of how our data set can provide insights into the changes in composition of macromolecular complexes in different tissues. About half of the 130 genes in *C. elegans* belonging to the integrin adhesome set are broadly expressed between tissues and time points; this suggests that they are used both maternally and zygotically and that they may represent core proteins of integrin-based adhesions (Supplemental Fig. S12). In contrast, 32 genes have gene expression restricted to muscle, including the known muscle genes *unc-89* (obscurin), *dys-1* (dystrophin), and the muscle-specific integrin *pat-2*. The specialized proteins that these genes encode presumably reflect the very different demands that contraction places on the adhesome structures in muscle. Another 18 genes are enriched in muscle but have low to moderate expression in other tissues, including *tln-1* (talin), *unc-112* (kindlin), and *unc-97* (PINCH), indicating either shared usage or tissue-specific isoforms. Another set of genes is expressed only at lower levels in muscle with primary expression in one or more other tissues. These include genes primarily expressed in the intestine—*plc-3* (phospholipase C), *pkc-2* (protein kinase 2), and *act-5* (actin)—and neurons *tiam-1* (TIAM) and *jnk-1* (Jun N-terminal kinase). Our findings also shed new light on prior genetic studies. For example, the gene *pxl-1* (paxillin), when mutated, results in death in the larval stage L1 owing to failure of the pharynx to pump (Warner et al. 2011), yet our data show *pxl-1* is most highly expressed in neurons, indicating a potential role in neuronal adhesion structures; the early death from pharyngeal dysfunction may have masked neuronal abnormalities. The expression data show that although integrin-based adhesions may share a substantial set of core proteins, the complexes require cell-specific components to accommodate specialized functions.

Finally, we used the plot of genes in 35-dimensional space to evaluate the expression of genes within operons, calculating the expression distance between adjacent genes in operons (Supplemental Table S6). The RNA levels of a downstream gene could differ from its upstream gene because of post-transcriptional mechanisms but also because of the use of independent promoters for the downstream gene. To detect the latter, we assayed the fraction of splice leader SL1 *trans*-splicing, which occurs predominantly at the initiation of the transcript, whereas SL2 is used predominantly in downstream operon genes (Allen et al. 2011). Although not always true for individual genes, SL1 usage as a fraction of SL1 + SL2 usage increased as expression distance increased (Supplemental Fig. S13), in accord with the notion that the gene has an independent promoter as well as being part of an operon. We also noted that the fraction of SL2 used in downstream genes decreased as expression increased, confirming our prior results (Supplemental Fig. S14; Boeck et al. 2016). Similarly, downstream genes with high expression distances more frequently had clusters of transcription factor binding sites (as measured by ChIP-seq) than those with low distances (Supplemental Fig. S15; Kudron et al. 2018), again suggesting the existence of an independent promoter for the downstream gene. The presence of a second promoter suggests these genes, while operating as an operon in some tissues, can be expressed independently in others. These observations suggest that independent promoters can contribute to the differences in expression patterns between genes in operons, but the absence of stronger correlations suggests that post-transcriptional mechanisms also play important roles.

### Differential exon, splice site, and splice leader usage

Because we obtained sequence reads from the entire length of transcripts, we were able to use JunctionSeq (Hartley and Mullikin 2016) to assay time- and tissue-specific usage of exons, splice junctions, splice leaders, and alternative initial and terminal exons. Overall, at least one third of the alternative exons and splice junctions annotated in WormBase show evidence of differential usage (defined as between two or more tissues at one or more time points compared to the rest of the gene) (Supplemental Table S7). Differentially used exons are more likely to be conserved in *Caenorhabditis briggsae* orthologs (85% vs. 75%; *Z*-score *P* < 0.0001). Also, we find that DE genes are ∼10% more likely to contain differentially used splice junctions.

Of the different types of alternative splicing, mutually exclusive exons show the most specificity, with ∼43% showing differential usage among tissues (supported by both exon and splice junction reads) and an additional 23% showing evidence from either exon or splice junction reads alone (Supplemental Table S7). In contrast, splice sites producing exons of different lengths showed the least specificity, with 70% showing no evidence of differential usage. Possibly, exons differing only by a few bases are functionally similar, with little advantage conferred by tissue-specific expression or they may be functionally unique, but their largely shared sequence may make detecting differences more difficult (Supplemental File S2).

Of the 2601 WormBase-annotated genes (WS245) with alternative start sites, 916 genes (1614 first exons and splice junctions) show some pairwise differential usage of the first exons (log_2_FC ≥ 1, *P*adjust ≤ 0.1) (Supplemental Tables S8, S9a–d). Further, there are 89 initial exons and 24 initial splice junctions that are significantly more frequently used (log_2_FC ≥ 1, *P*adjust ≤ 0.1) in one tissue than in all others, and there are 49 initial exons and 13 initial splice junctions that were significantly less frequently used in one tissue than in all other tissues (Supplemental Table S10).

In *C. elegans*, although earlier work suggested that 70% of mRNAs are *trans*-spliced to one of two spliced leaders, SL1 or SL2 (Allen et al. 2011), more recent estimates suggest as many as 85% are *trans*-spliced (Tourasse et al, 2017). We identified a total of 8830 sites (6190 genes) where splice leaders are added to a transcript, with 5099 (58%) of sites SL1 only, 2064 (23%) of sites SL2 only, and 1667 (19%) of sites both an SL1 and an SL2 (Supplemental File S3; Supplemental Table S11). Of the 2601 genes with alternative start sites, 1423 of these showed evidence of splice leader usage, including some SL sites that were tissue-specific. We also found broadly expressed genes that were only *trans*-spliced in one tissue and genes in which the acceptor site, and sometimes the splice leader type, varied between tissues (e.g., Supplemental Figs. S16, S17; Supplemental Tables S12–S14). These results reveal novel complexity in the regulation of splice leader usage.

**Figure 4.**
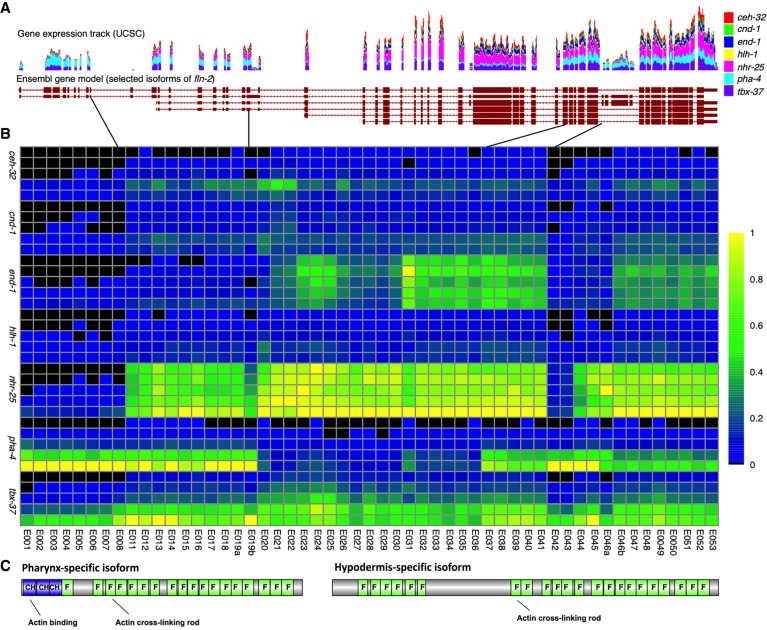
Differential exon usage for *fln-2*. Exon usage is highly differential between tissues for *fln-2*. (*A*) Selected isoforms of *fln-*2 with the UCSC gene expression track containing our data are displayed. Scale is zero to 2050 reads for time point 4 (normalized to 20 million total reads per sample). (*B*) In the heatmap displaying usage, tissues are organized by time from T0 to T4 along the *y*-axis within each tissue (as labeled), and exons are arranged by order on the *x*-axis. Expression values are normalized to a maximum of one for each exon and then colored by the percentage of expression compared with the maximum. Expression ranges from black (0) to yellow (1). (*C*) The resulting major protein products for the pharynx and hypodermis are shown, highlighting the major differences in number of filamin domains (label F) between the two products, as well as the total absence of calponin homology (label CH) domains in the hypodermal product.

Alternative splicing events sometimes involve multiple exons, producing substantially different proteins in one tissue versus another. We see examples of this in integrin adhesome genes, including previously published examples such as tropomyosin (*lev-11*) (Watabe et al. 2018) and perlecan (*unc-52*) (Rogalski et al. 1995). We also see novel alternative splicing events such as those that occur in filamin (*fln-2*), one of two genes encoding a filamin protein in the worm genome. The hypodermal isoform has an alternative start site and lacks the first 10 exons included in the pharyngeal isoform, as well as exons 42 and 43 (Fig. 4). In addition, a novel splice junction (missing from WormBase and other current models; Chr X: 9,407,498–9,420,709) skips exons 20–36 in the pharyngeal isoform, exons that are present in the hypodermal isoform. Together, these alternative splice forms result in substantial differences in the predicted proteins. The initial 10 exons of the pharyngeal isoform code for three calponin homology actin binding domains, absent in the hypodermal isoform. However, because the pharyngeal form skips 17 exons, it has fewer IG/Fil domains and lacks additional low complexity domains (Supplemental File S4) present in the hypodermal isoform. Intestinal *fln-2* codes for a protein most similar to the hypodermal isoform but contains only 13 IG/Fil domains. The ABa lineage produces cells in both pharyngeal and hypodermal tissues and appears to have both forms.

A second integrin adhesome gene, *tln-1*, also displays tissue-specific isoform usage that results in substantially different proteins. It has an alternative 3′ exon that is used almost exclusively in muscle (Supplemental Fig. S18). As a result, the shorter, muscle-specific isoform lacks the three VL actin binding domains and one of the two I/L WEQ binding domains present in the longer isoform. The shorter form also lacks the single Talin-½ domain that is predicted to facilitate disengagement of TLN-1 from the integrin. We speculate that in muscle integrin adhesion complexes (dense bodies), the absence of this dissociation domain contributes to the stability of the dense body in contrast to the less stable complexes found in migrating cells. The adhesome components thus differ substantially not only in the genes used but also in the specific isoforms produced by differential processing.

### RNA editing

RNA editing is the post-transcriptional modification of RNA by nucleotide modification/substitution, insertion, or deletion, increasing the number of different transcripts that can be produced (Zhao et al. 2015) by changing RNA structure and splicing patterns (Hundley and Bass 2010) and by regulating coding potential/expression (Kang et al. 2015). We looked for evidence of RNA editing in our data set to find support for the 11 A-to-G RNA editing sites identified in *C. elegans* by Zhao et al. (2015; Supplemental File S5). Although some support was found for six of the sites, strong support was found for editing in only one gene, *Y105E8A.3*, with no resulting change in the amino acid. No support was found for the C-to-U editing event suggested by Wang et al. (2004). Instead, the 1312 reads across our samples confirmed the consensus base.

By using an analysis pipeline similar to that of Zhao et al. (2015; Methods), we identified a total of 48 putative edits in coding exons that occurred in at least two samples and not in the corresponding unlabeled samples (Supplemental File S5). A total of 17 of the 48 edits resulted in an amino acid change (Supplemental Tables S15, S16), and one, in *C07E3.9*, resulted in a premature stop truncating the final 27 amino acids.

### Gene regulation

The combined tissue and temporal specificity of the data provide an opportunity to examine the regulation of gene expression. We used the data to ask (1) if genes potentially sharing upstream regulatory regions were more likely to show similar expression patterns, (2) which transcription factors are expressed in which tissues and in what order, and (3) which transcription factor binding sites were more likely to be associated with tissue-specific genes.

### Gene regulation—shared regulatory regions

We first asked if divergently transcribed genes (so-called head-to-head genes), which might be influenced by common regulatory elements, were more likely to have similar patterns of expression than other gene orientations (head-to-tail and tail-to-tail) or random pairs. We considered downstream genes in operons separately, because for the most part their expression is expected to be similar to the initial gene. We then compared the expression of gene pairs in 35-dimensional space for each class of genes.

**Figure 5.**
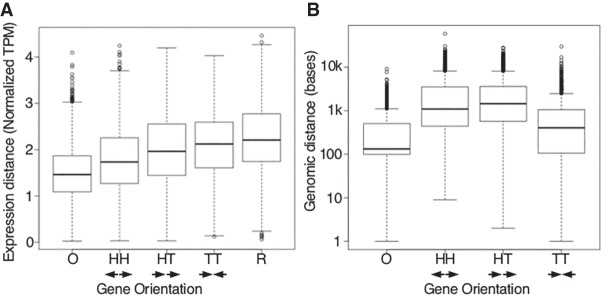
Gene expression similarities compared with regulatory regions. (*A*) Genes that have their promoter regions shared (in operons and head-to-head) have significantly more related spatiotemporal gene expression as measured by the Euclidean distance of 35-dimensional expression space than do genes oriented head-to-tail, tail-to-tail, or random pairs. (*B*) Genomic distance of gene pairs. Labels for gene orientation are as follows: (O) operon; (HH) head-to-head; (HT) head-to-tail; (TT) tail-to-tail; (R) random. Arrows indicate transcriptional direction of the genes analyzed.

As expected, gene pairs within an operon were found to be significantly similar in their spatiotemporal expression using a Student's *t*-test (Fig. 5A). Outside of operons, genes oriented head-to-head are also significantly more similar in gene expression values in time and space than are genes that were head-to-tail, tail-to-tail, or random pairs. Head-to-head genes are not statistically different in their genomic distance from one another than head-to-tail genes (Fig. 5B), consistent with the notion that head-to-head genes may be sharing regulatory information. These intergenic spaces presumably contain regulatory regions and binding sites for transcriptional regulators, with their lengths indirectly reflecting the amount of regulatory sequence (Fig. 5B).

### Gene regulation—transcription factor expression patterns

We modified a list of annotated transcription factors (Fuxman Bass et al. 2016), using more recent data to include 932 genes (Supplemental Table S17). Of these, 689 have robust gene expression in at least one of our samples (TPM ≥ 15). In total, 178 transcription factors show differential expression based on DESeq2 analysis and 259 transcription factors based on fuzzy *k*-means clustering (Supplemental Table S5; for gene lists, see Supplemental Tables S18, S19). In muscle, known muscle transcription factors are present such as *hlh-1* (Chen et al. 1994), *unc-120* (Williams and Waterston 1994; Baugh et al. 2005), and *pat-9* (Williams and Waterston 1994; Liu et al. 2012). However, additional transcription factors without a previously annotated role such as *snai-1* and *M03D4.4* are clearly early embryonic muscle-specific transcription factors. We also identified transcription factors with more complex usage patterns from our fuzzy *k-*means clustering (Fig. 2). The 25 transcription factors in cluster 36 potentially regulate genes in the intestine and pharynx, as their gene expression is enriched in both of these tissues.

The temporal resolution of our data also provides information about the order in which transcription factors act within a tissue. For example, the different intestinal clusters reflect different temporal patterns of expression. The GATA factor *end-1* (Zhu et al. 1997; Boeck et al. 2011) is important for driving gene expression for a number of early intestinal genes, and it clusters with early expressing intestinal genes in our fuzzy *k*-means clustering (Figs. 2, 3). The intestinal-specific transcription factor *elt-2,* which is downstream from *end-1* in the transcriptional regulatory pathway (McGhee et al. 2007), clusters with genes with later expression. In contrast, the transcription factor *F55B11.4*, which has Zn finger (C2H2) domains and ELT-2 binding sites in its promoter region, clusters with genes sharing late-onset intestinal expression and is a transcription factor with almost exclusively intestinal gene expression beginning ∼400 min into development. This places *F55B11.4* downstream from both *end-1* and *elt-2* in the intestinal transcriptional regulatory pathway, with presently unknown regulatory targets. For genes in which the expression is tissue-specific, we can also use the whole-embryo gene expression data (Boeck et al. 2016), which have denser and wider sampling across time, to refine the order of expression. For example, the transcription factor *M03D4.4*, which has Zn-finger (C2H2) domains and an HLH-1 binding site in its promoter region, is enriched in muscle; in the whole-embryo data, it has a temporal profile almost identical to that of *unc-120*, which follows *hlh-1* and precedes *ceh-18*.

### Gene regulation—transcription factor gene regulation

**Figure 6.**
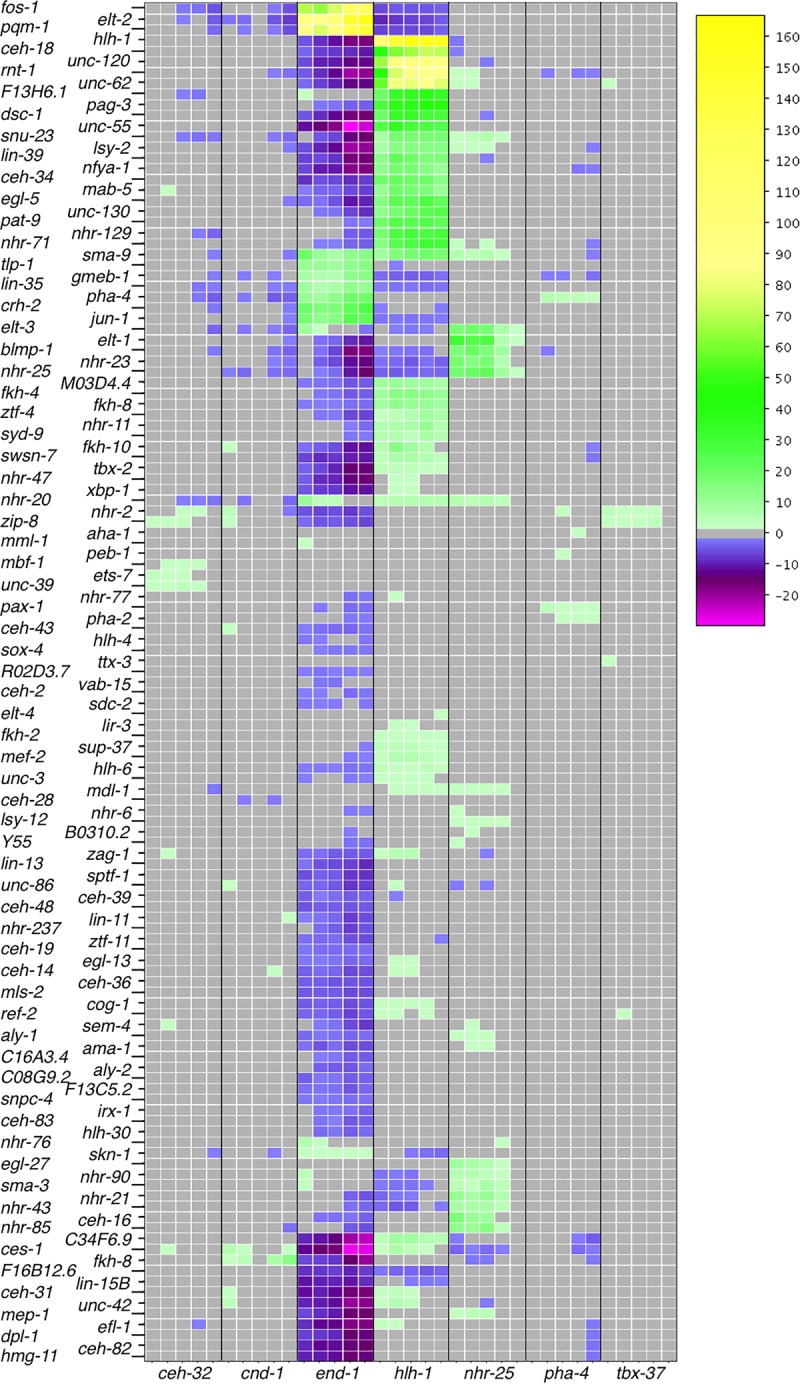
Regulation of DE genes by transcription factors. Transcription factors are both more highly associated with DE genes in tissues (yellow) or significantly less associated with DE genes in a tissue (magenta) than by a random sampling of ChIP-seq peaks. Tissue time points marked in gray indicate no significantly increased or decreased presence of transcription factor binding sites with a *P*-value cutoff of 0.01. This heatmap displays −log(*P*-values) for each transcription factor by tissue/time for transcription factors enriched in the set of DE genes. If a transcription factor is depleted in the set of DE genes, then log(*P*-value) is displayed, making the values less than zero. Any transcription factor that did not achieve significance in any tissue/time (*P*-values all >0.01), either enriched or depleted, was not placed into the heatmap. *Y55A3AM.14* is abbreviated as “*Y55*.”

Growing numbers of *C. elegans* transcription factors have binding site information as a result of ChIP-seq experiments performed as part of the model organism Encyclopedia of Regulatory Networks (modERN) project (Kudron et al. 2018; data are available at http://epic.gs.washington.edu/modERN/). We analyzed the binding sites for these transcription factors to determine which transcription factors showed a bias toward binding in the regulatory region of DE genes. For each transcription factor, we compared the percentage of DE genes in a given tissue that had a ChIP-seq peak to the percentage of non-DE genes with a ChIP-seq peak, and calculated the significance of the difference (Methods). Out of 183 transcription factors, 127 showed either a significantly enhanced presence in front of DE genes or a significantly reduced presence in front of DE genes in any given time point of a tissue (Fig. 6).

Transcription factors with established roles in specific tissues, such as *hlh-1* and *unc-120* in muscle (Chen et al. 1994; Baugh et al. 2005), *elt-2* in intestine (McGhee et al. 2007), and *elt-1* (Spieth et al. 1991) and *elt-3* (Gilleard et al. 1999) in hypodermis, have a substantially increased presence in front of DE genes in the expected tissue. We also see genes that do not have a previously defined role, such as *M03D4.4* in muscle, also differentially expressed in muscle. Together, the differential expression of transcription factors and the differential association of genes enriched in a tissue suggest a direct regulatory relationship.

## Discussion

We have dissected gene expression with lineage and tissue/organ resolution by producing deeply sequenced RNA-seq libraries from early in embryonic development until cuticle formation. Sequence reads were obtained across whole transcripts, providing information into the spatial and temporal usage of alternative splice sites, including novel introns and exons, and different start sites, including those defined by splice leader sequences. We have shown the utility of this information by focusing on the integrin adhesome, providing insights into how integrin-based adhesions may differ between cell types. The scientific community can similarly apply our data set to their specific research area and gain insight into the transcriptional regulation and dynamics taking place during embryonic development.

The diversity of alternative exon usage by tissue and time is apparent in many cases, especially in genes with complex splice patterns. In the examples of *tln-1* and *fln-2*, with the combination of spatial and temporal resolution, we were able to attribute expression of individual isoforms to specific cell lineages and tissues. Although these genes have fairly broad expression overall, specific isoforms clearly have differential expression in both time and space. Looking at differences in overall gene expression only reveals part of the complexity.

Our data set also provides insights into transcriptional regulation based on both time- and tissue-based differential gene expression. We have identified new tissue-specific transcription factors as well as those shared between different tissues. The temporal information suggests the order in which transcription factors act in potential regulatory cascades. By combining our data with the ChIP-seq data from the modERN project (Kudron et al. 2018), we have uncovered intriguing regulatory relationships. The power of our approach is limited by the number of genes enriched for expression in a given tissue and by the number of ChIP-seq peaks associated with any transcription factor. Nonetheless, our analysis reveals roles for various transcription factors known to be involved in tissue differentiation such as HLH-1, UNC-120, END-1, ELT-2, etc. It also revealed activities for factors used in just a subset of cells, such as MAB-5, EGL-5, and CEH-34. With more detailed expression information and improved ChIP-seq data, the approach should reveal still further relationships.

The integrin adhesome genes provide an example of some ways our data set can inform the study of larger complexes. The essentiality of some of the genes in the complex for muscle function has inhibited the genetic analysis of these genes in other tissues. These other roles include cell migration mediated by focal adhesion-like structures, distal tip cell migration, axon extension, pharyngeal muscle cell adhesions, neuronal point contacts found in growth cones, and anchor cell invasion. By examining their expression across the various tissues, we are able to see distinct groups of genes with substantially different expression patterns (Supplemental Fig. S12), including genes expressed exclusively or mainly in muscle, some primarily in other tissues, and some broadly expressed across tissues and times. We also saw that alternative splicing produces very different forms of the genes *fln-2* and *tln-1* in different tissues, allowing for further specialization of adhesome function.

Our time series data sets for seven different sets of cells during critical development stages quantify the gene expression dynamics and differences that occur between different cell types during *C. elegans* embryonic development, as well as the changes within tissues over time. Extending the analysis further into embryogenesis and to postembryonic stages is a logical next step. Recent advances in single-cell RNA-seq technologies should help dissect gene expression to more refined series of cell types, even individual cells (Cao et al. 2017). But because at present these methods fail to assay the full length of the transcript, additional studies will be required to reveal the full complexity of gene expression. By continuing to tease apart global gene expression in more comprehensive and sensitive ways, scientists will better understand how both the major and the more subtle gene expression differences between cell types shape development.

## Methods

### Embryo synchronization and isolation

Strains were developmentally synchronized using rounds of KOH/sodium hypochlorite treatment and plating of freshly hatched L2 animals onto 150-mm peptone-rich NGM plates seeded with NA22 bacteria. To degrade the eggshell, 1 U/mL Chitinase (C6137 Sigma-Aldrich) was added to the embryos at a ratio of 1 mL Chitinase to 0.5 mL embryo suspension, and the embryos were transferred to 30-mm petri dishes to incubate before embryo dissociation. For details, see Supplemental Methods.

### Embryo dissociation and cell sorting

To dissociate the embryos into single cells, 100 µL of 15 mg/mL Pronase (P6911 Sigma-Aldrich) was added to ∼1.5 mL of embryo/egg buffer suspension. The cell suspension was drawn from the dish using a 3 cc syringe and used to wash the dish to isolate all embryos. Embryos were repeatedly pulled through a 21-gauge needle 20×, in a 1.5-mL Eppendorf tube, and then incubated at room temperature for 5 min. Lastly, the cell suspension was drawn repeatedly through a 21-gauge needle until a suspension of single cells was confirmed via microscope. Cells were sorted using a FACS ARIA III to isolate a minimum of 150,000 cells per sample and chilled during sorting. For details, see Supplemental Methods.

### RNA isolation and library construction

Total RNA was isolated using TRIzol LS/chloroform extraction of total RNA, followed by EtOH precipitation and column-based cleanup using Direct-zol tubes (Zymo Research). Ribosomal RNA was reduced in each sample using Ribo-Zero (Illumina) and purified using SPRI beads (Agencourt RNAClean XP). Immediately following the RNA cleanup, first-strand cDNA synthesis was performed using SuperScript IV (Invitrogen), followed by second-strand synthesis using the second-strand synthesis module (NEB). Shearing of cDNA was performed using a Covaris LE220 and end repaired using the NEB end repair module. Y-Adapter ligation to A-tailed cDNA was followed by QPCR-based barcoding of samples.

### Sequencing and gene expression calculations

Sequencing was performed using Illumina HiSeq 2500 and NextSeq 500 instruments to obtain 50-bp paired end reads or 75-bp paired end reads, respectively. Data were demultiplexed by barcode into individual sample FASTQ files using bcl2FASTQ. Reads were aligned with STAR version 2.4.2a (Dobin et al. 2013) to the WS245 genome sequence from WormBase with a maximum intron size of 25,000 (Supplemental Table S20). Transcript-level expression was calculated using BitSeq (Glaus et al. 2012), and these values were used to calculate TPM for the transcripts (Supplemental Tables S21, S22). TPM values were averaged for the replicates of each tissue's time point to obtain a TPM for each tissue. To test saturation of sequencing, we used the program subSeq (Supplemental Fig. S19; Robinson and Storey 2014). For details, see Supplemental Methods.

### Differential gene expression calculations

By using DESeq2 differential expression analysis, we identified genes expressed predominantly in a single tissue within each time point using TPM values as input. For each gene, we report the ratio of the largest TPM expression value to the second largest TPM expression value (excluding related tissues as shown in Supplemental Table S1) along with the DESeq2 adjusted *P*-value (Supplemental Table S2). We also used a chi-square test to assess whether our calculation of a gene being stably expressed was independent of the gene's presence in an operon.

### Fuzzy *k*-means clustering and t-SNE

For clustering of gene expression, TPM values were normalized, and the replicates were averaged. TPM values were divided by the maximum TPM value across all the replicate averaged samples for each gene to give “max1 normalization” as it converts the maximum expression value for each gene across all samples to one. These max1 replicate averaged values were used as input to the t-SNE dimensional reduction. After multiple rounds of clustering, a total of 60 clusters was chosen as this created distinct groups of genes in terms of temporal and spatial gene expression with minimal internal variation.

### Transcription factor regulation analysis

ChIP-seq peaks were obtained from the modERN (Kudron et al. 2018) and modENCODE (Araya et al. 2014) projects using ChIP-seq experiments for embryonic and early larval (L1, L2, L3) stages for analysis. The probability that a target gene would have a transcription factor in its associated cluster was calculated for each transcription factor across the genome based on the overall number of ChIP-seq peaks for each transcription factor used in the analysis. We then looked at DE genes in each tissue and time to see if there was an over- or underrepresentation of any particular tissue/time samples proximal to ChIP-seq peaks for each transcription factor. For details, see Supplemental Methods.

### Data processing and analysis

All scripts used to process and analyze these data are found in the Supplemental Material. A summary file is provided with descriptions of the scripts, and an additional description is provided within scripts when clarification is needed. Java programs are also included along with a summary file describing the files used and programs required. The file Supplemental_Guide.xlsx in the Supplemental Material catalogs all supplemental material and provides a description of each file.

## Data access

All sequencing data from this study have been submitted to the NCBI BioProject database (https://www.ncbi.nlm.nih.gov/bioproject) under accession number PRJNA477006. Expression profiles across tissues and time points for individual genes are available at http://genome.sfu.ca/gexplore (see Supplemental Fig. S20 for an example).

## Supplementary Material

Supplemental Material
