# Supplementary material for: The C. elegans embryonic transcriptome with tissue, time, and alternative splicing resolution
Source: Genome Res. 2019 Jun;29(6):1036–45. doi: 10.1101/gr.243394.118 (PMC6581053; doi:10.1101/gr.243394.118)
Supplement: Supplemental Material [file supp_29_6_1036__index.html]

The C. elegans embryonic transcriptome with tissue, time, and alternative splicing resolution — Supplemental Material 

# The *C. elegans* embryonic transcriptome with tissue, time, and alternative splicing resolution

## Supplemental Material

- Supplemental\_Code.zip
- Supplemental\_File\_S1.zip
- Supplemental\_File\_S2.zip
- Supplemental\_File\_S3.bed
- Supplemental\_File\_S4.txt
- Supplemental\_File\_S5.xlsx
- Supplemental\_Guide.xlsx
- Supplemental\_Methods.docx
- Supplemental\_Table\_S2a.txt
- Supplemental\_Table\_S2b.txt
- Supplemental\_Table\_S3.doc
- Supplemental\_Table\_S4.txt
- Supplemental\_Table\_S5.txt
- Supplemental\_Table\_S6.txt
- Supplemental\_Table\_S7.docx
- Supplemental\_Table\_S9a.txt
- Supplemental\_Table\_S9b.txt
- Supplemental\_Table\_S9c.txt
- Supplemental\_Table\_S9d.txt
- Supplemental\_Table\_S10a.txt
- Supplemental\_Table\_S10b.txt
- Supplemental\_Table\_S11.txt
- Supplemental\_Table\_S12.txt
- Supplemental\_Table\_S13.txt
- Supplemental\_Table\_S16.doc
- Supplemental\_Table\_S17.xlsx
- Supplemental\_Table\_S18.doc
- Supplemental\_Table\_S19.doc
- Supplemental\_Table\_S20.xlsx
- Supplemental\_Table\_S21.txt
- Supplemental\_Table\_S22.txt
- Supplemental\_Material.docx
