# Supplementary material for: The C. elegans embryonic transcriptome with tissue, time, and alternative splicing resolution
Source: Genome Res. 2019 Jun;29(6):1036–45. doi: 10.1101/gr.243394.118 (PMC6581053; doi:10.1101/gr.243394.118)
Supplement: Supplemental Material [file supp_gr.243394.118_Supplemental_File_S1.zip › biological_process.end1.pdf]

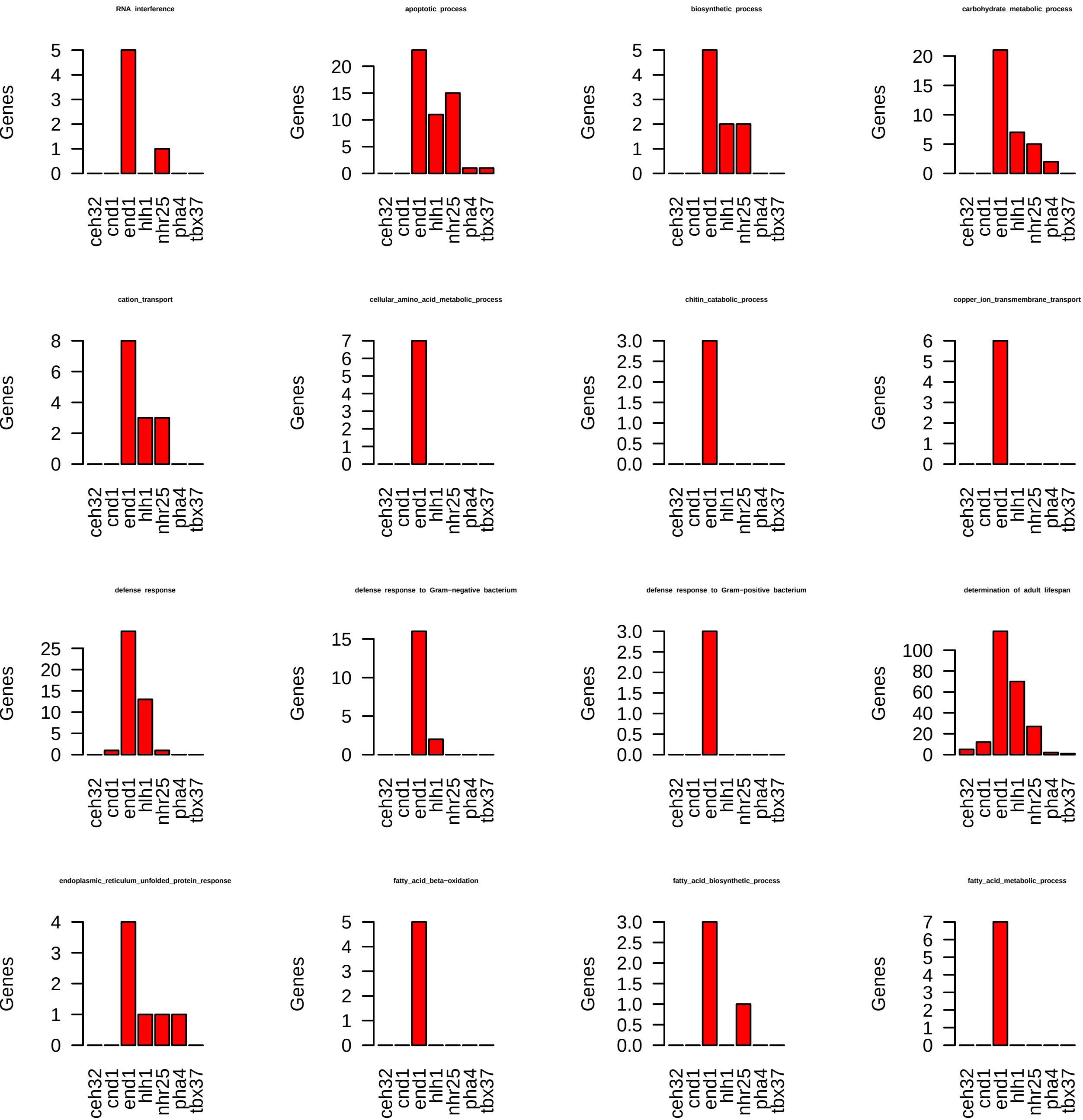
RNA_interference
apoptotic_process
biosynthetic_process
carbohydrate_metabolic_process
cation_transport
cellular_amino_acid_metabolic_process
chitin_catabolic_process
copper_ion_transmembrane_transport
defense_response
defense_response_to_Gram−negative_bacterium
defense_response_to_Gram−positive_bacterium
determination_of_adult_lifespan
endoplasmic_reticulum_unfolded_protein_response
fatty_acid_beta−oxidation
fatty_acid_biosynthetic_process
fatty_acid_metabolic_process
Genes
ceh32
cnd1
end1
hlh1
nhr25
pha4
tbx37

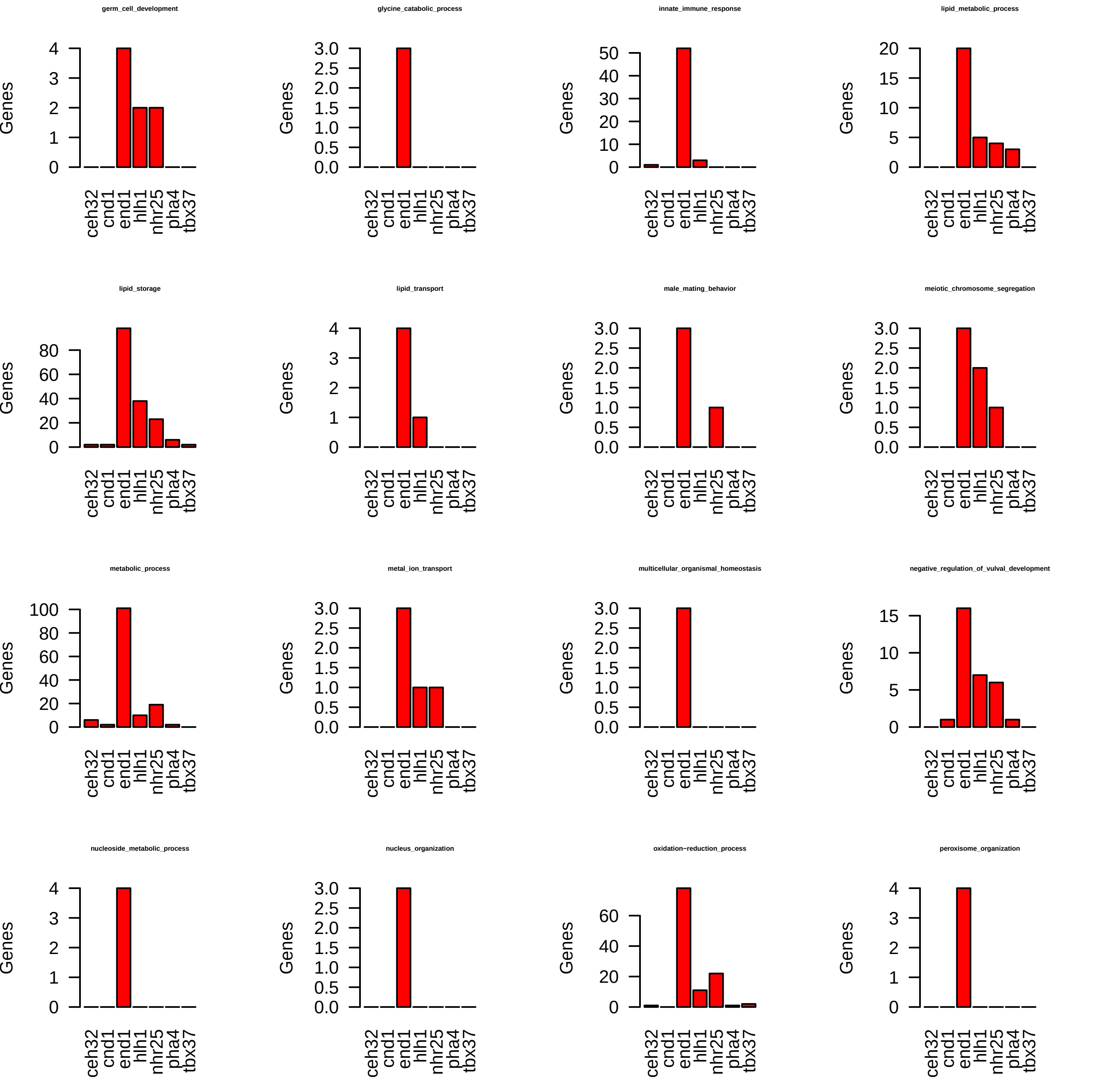

germ_cell_development
Genes
0 1 2 3 4
ceh32 cnd1 end1 hlh1 nhr25 pha4 tbx37
glycine_catabolic_process
Genes
0.0 0.5 1.0 1.5 2.0 2.5 3.0
ceh32 cnd1 end1 hlh1 nhr25 pha4 tbx37
innate_immune_response
Genes
0 10 20 30 40 50
ceh32 cnd1 end1 hlh1 nhr25 pha4 tbx37
lipid_metabolic_process
Genes
0 5 10 15 20
ceh32 cnd1 end1 hlh1 nhr25 pha4 tbx37
lipid_storage
Genes
0 20 40 60 80
ceh32 cnd1 end1 hlh1 nhr25 pha4 tbx37
lipid_transport
Genes
0 1 2 3 4
ceh32 cnd1 end1 hlh1 nhr25 pha4 tbx37
male_mating_behavior
Genes
0.0 0.5 1.0 1.5 2.0 2.5 3.0
ceh32 cnd1 end1 hlh1 nhr25 pha4 tbx37
meiotic_chromosome_segregation
Genes
0.0 0.5 1.0 1.5 2.0 2.5 3.0
ceh32 cnd1 end1 hlh1 nhr25 pha4 tbx37
metabolic_process
Genes
0 20 40 60 80 100
ceh32 cnd1 end1 hlh1 nhr25 pha4 tbx37
metal_ion_transport
Genes
0.0 0.5 1.0 1.5 2.0 2.5 3.0
ceh32 cnd1 end1 hlh1 nhr25 pha4 tbx37
multicellular_organismal_homeostasis
Genes
0.0 0.5 1.0 1.5 2.0 2.5 3.0
ceh32 cnd1 end1 hlh1 nhr25 pha4 tbx37
negative_regulation_of_vulval_development
Genes
0 5 10 15
ceh32 cnd1 end1 hlh1 nhr25 pha4 tbx37
nucleoside_metabolic_process
Genes
0 1 2 3 4
ceh32 cnd1 end1 hlh1 nhr25 pha4 tbx37
nucleus_organization
Genes
0.0 0.5 1.0 1.5 2.0 2.5 3.0
ceh32 cnd1 end1 hlh1 nhr25 pha4 tbx37
oxidation−reduction_process
Genes
0 20 40 60
ceh32 cnd1 end1 hlh1 nhr25 pha4 tbx37
peroxisome_organization
Genes
0 1 2 3 4
ceh32 cnd1 end1 hlh1 nhr25 pha4 tbx37

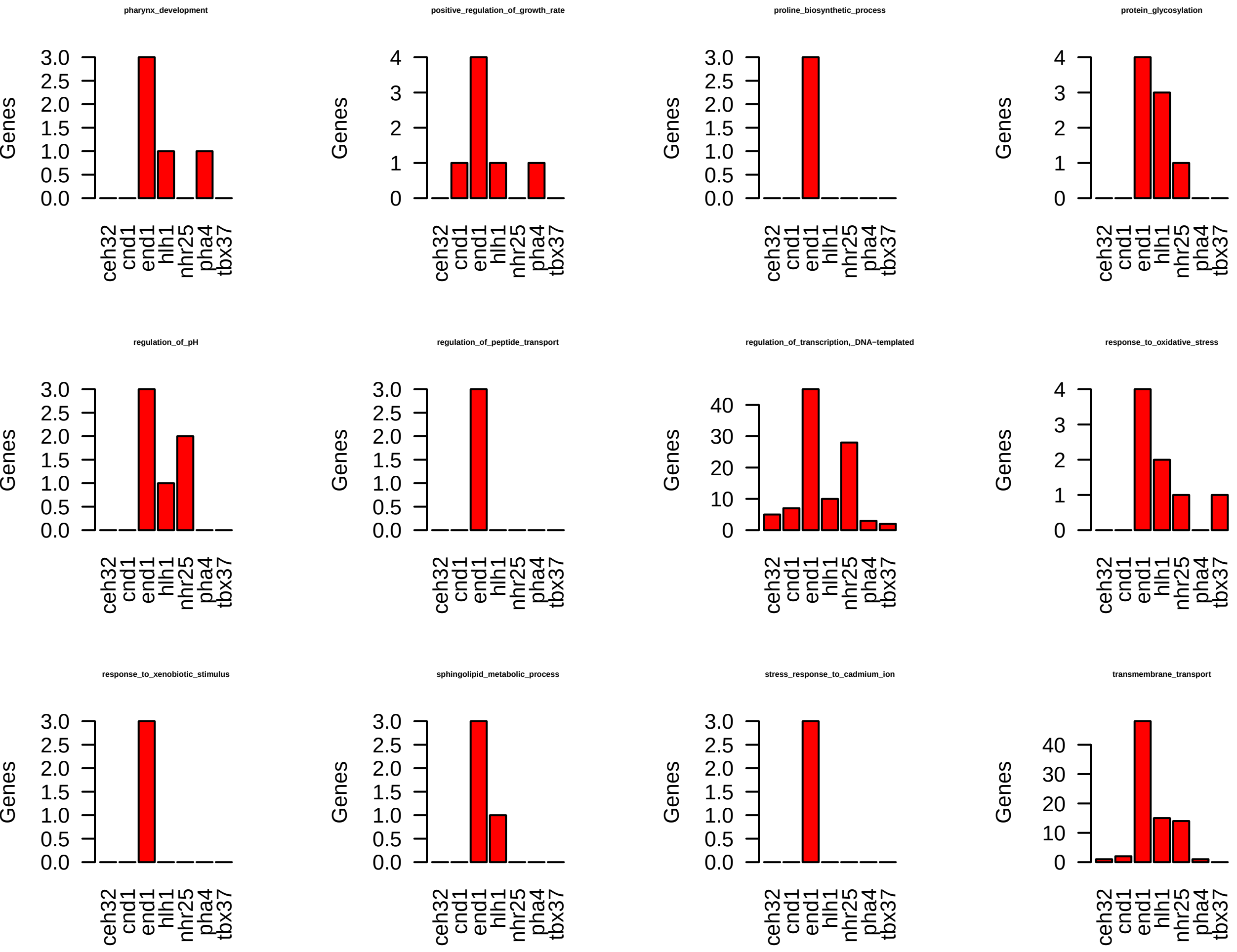

pharynx_development
positive_regulation_of_growth_rate
proline_biosynthetic_process
protein_glycosylation
regulation_of_pH
regulation_of_peptide_transport
regulation_of_transcription,_DNA−templated
response_to_oxidative_stress
response_to_xenobiotic_stimulus
sphingolipid_metabolic_process
stress_response_to_cadmium_ion
transmembrane_transport
Genes
ceh32
cnd1
end1
hlh1
nhr25
pha4
tbx37
