# Supplementary material for: The C. elegans embryonic transcriptome with tissue, time, and alternative splicing resolution
Source: Genome Res. 2019 Jun;29(6):1036–45. doi: 10.1101/gr.243394.118 (PMC6581053; doi:10.1101/gr.243394.118)
Supplement: Supplemental Material [file supp_gr.243394.118_Supplemental_File_S1.zip › biological_process.end1_hlh1.pdf]

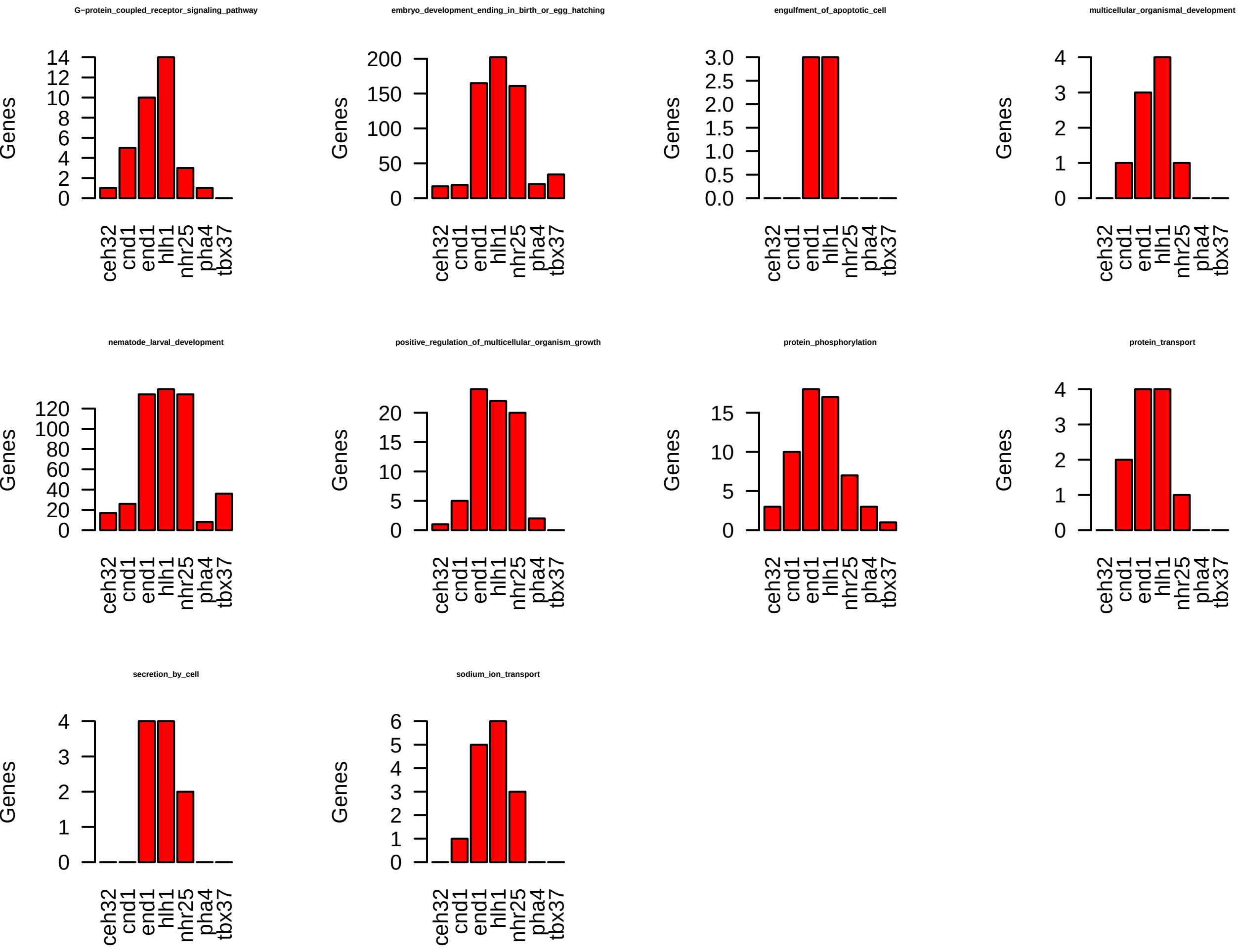

G−protein_coupled_receptor_signaling_pathway
embryo_development_ending_in_birth_or_egg_hatching
engulfment_of_apoptotic_cell
multicellular_organismal_development
nematode_larval_development
positive_regulation_of_multicellular_organism_growth
protein_phosphorylation
protein_transport
secretion_by_cell
sodium_ion_transport
Genes
ceh32
cnd1
end1
hlh1
nhr25
pha4
tbx37
