# Supplementary material for: The C. elegans embryonic transcriptome with tissue, time, and alternative splicing resolution
Source: Genome Res. 2019 Jun;29(6):1036–45. doi: 10.1101/gr.243394.118 (PMC6581053; doi:10.1101/gr.243394.118)
Supplement: Supplemental Material [file supp_gr.243394.118_Supplemental_File_S1.zip › biological_process.hlh1.pdf]

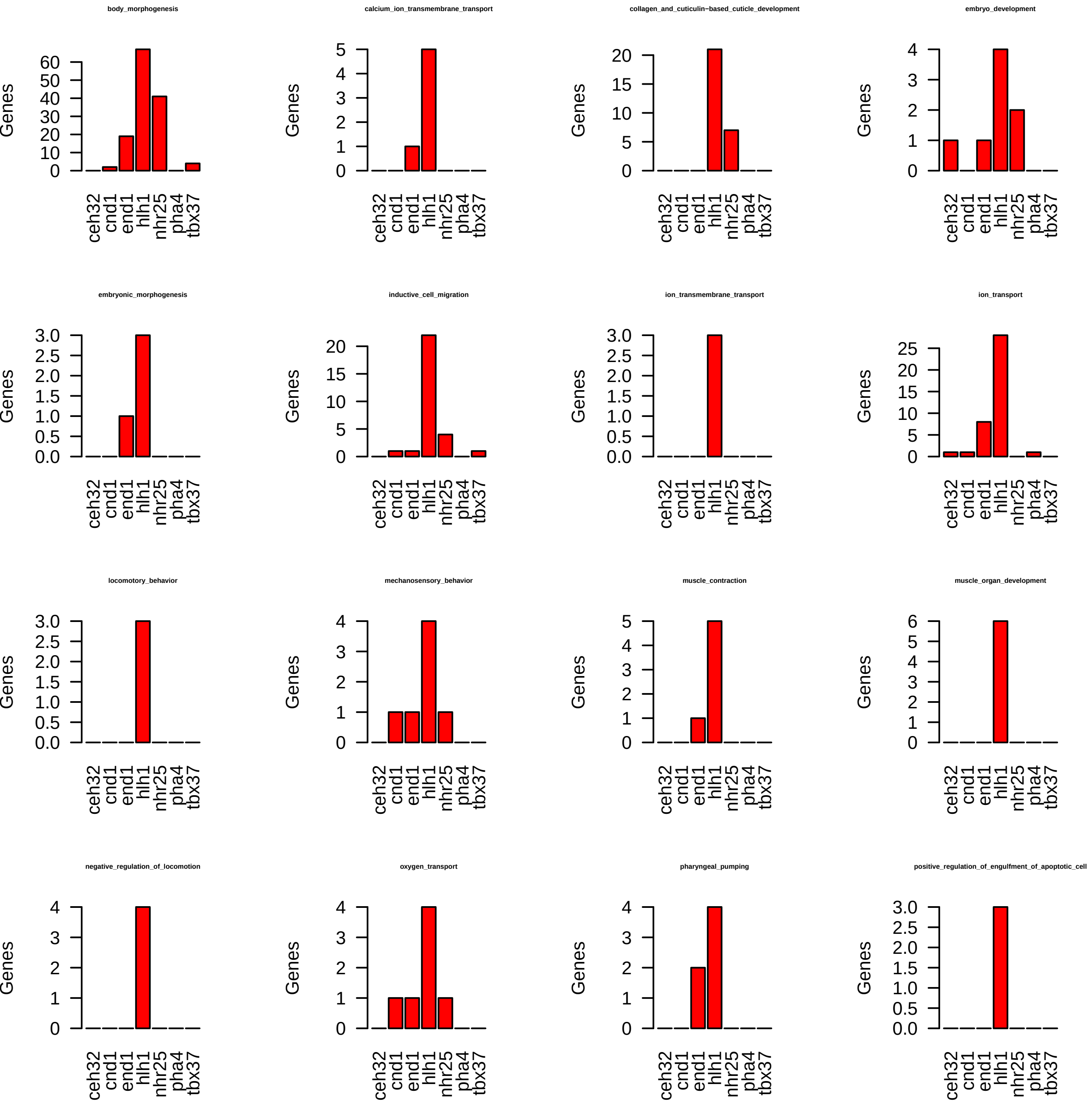
body_morphogenesis
calcium_ion_transmembrane_transport
collagen_and_cuticulin−based_cuticle_development
embryo_development
embryonic_morphogenesis
inductive_cell_migration
ion_transmembrane_transport
ion_transport
locomotory_behavior
mechanosensory_behavior
muscle_contraction
muscle_organ_development
negative_regulation_of_locomotion
oxygen_transport
pharyngeal_pumping
positive_regulation_of_engulfment_of_apoptotic_cell
Genes
ceh32
cnd1
end1
hlh1
nhr25
pha4
tbx37

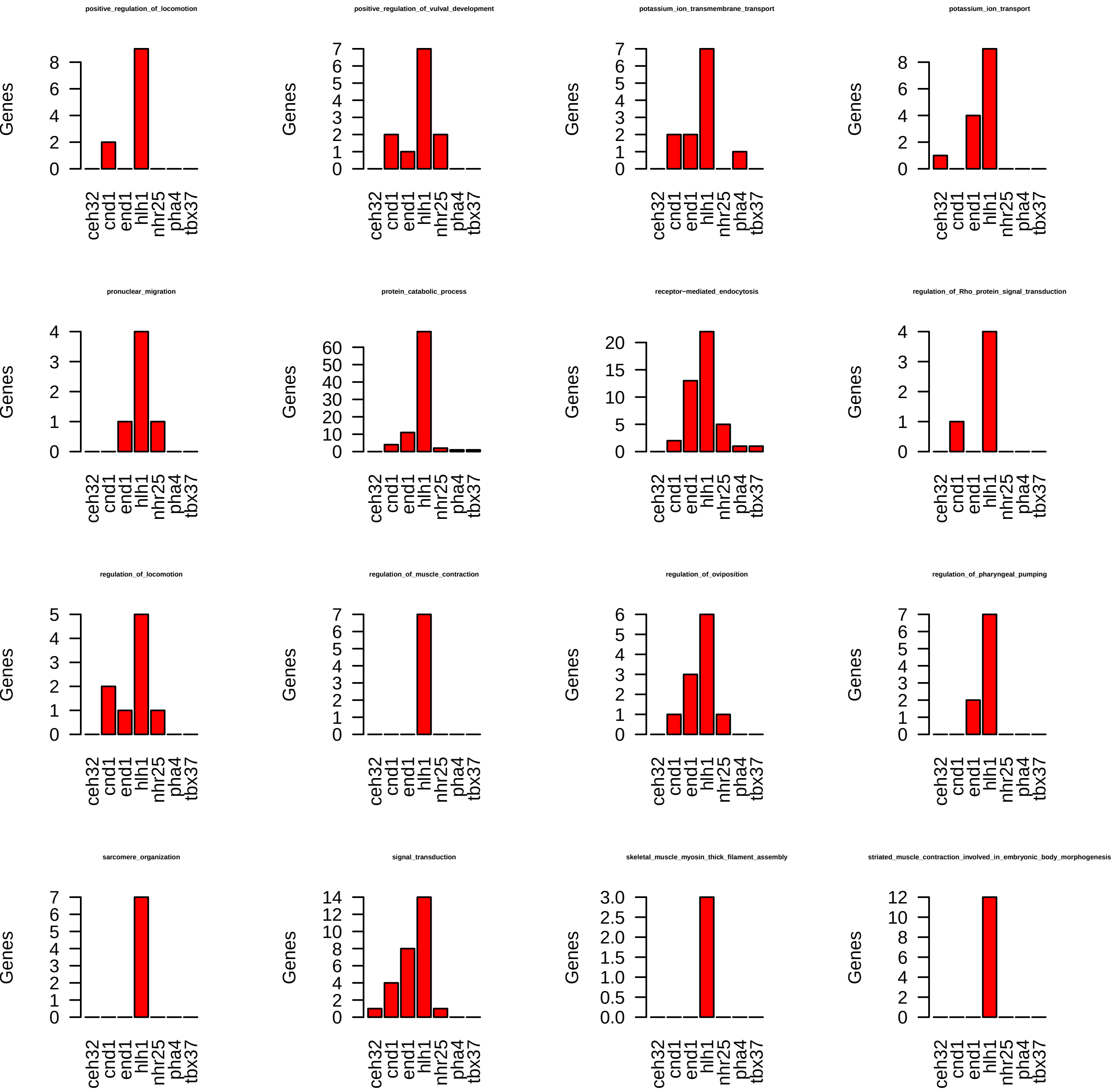
positive_regulation_of_locomotion
positive_regulation_of_vulval_development
potassium_ion_transmembrane_transport
potassium_ion_transport
pronuclear_migration
protein_catabolic_process
receptor−mediated_endocytosis
regulation_of_Rho_protein_signal_transduction
regulation_of_locomotion
regulation_of_muscle_contraction
regulation_of_oviposition
regulation_of_pharyngeal_pumping
sarcomere_organization
signal_transduction
skeletal_muscle_myosin_thick_filament_assembly
striated_muscle_contraction_involved_in_embryonic_body_morphogenesis
Genes
ceh32
cnd1
end1
hlh1
nhr25
pha4
tbx37

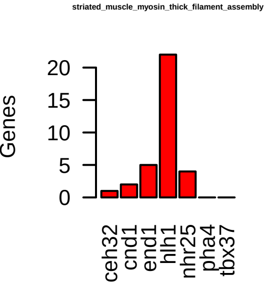
striated_muscle_myosin_thick_filament_assembly
Genes
0
5
10
15
20
ceh32
cnd1
end1
hlh1
nhr25
pha4
tbx37

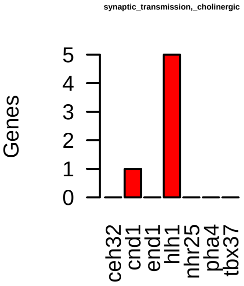
synaptic_transmission,_cholinergic
Genes
0
1
2
3
4
5
ceh32
cnd1
end1
hlh1
nhr25
pha4
tbx37

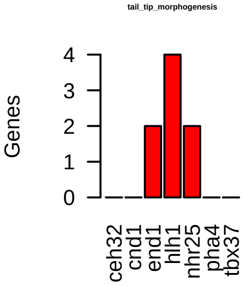
tail_tip_morphogenesis
Genes
0
1
2
3
4
ceh32
cnd1
end1
hlh1
nhr25
pha4
tbx37
