# Supplementary material for: The C. elegans embryonic transcriptome with tissue, time, and alternative splicing resolution
Source: Genome Res. 2019 Jun;29(6):1036–45. doi: 10.1101/gr.243394.118 (PMC6581053; doi:10.1101/gr.243394.118)
Supplement: Supplemental Material [file supp_gr.243394.118_Supplemental_File_S1.zip › biological_process.hlh1_nhr25_pha4.pdf]

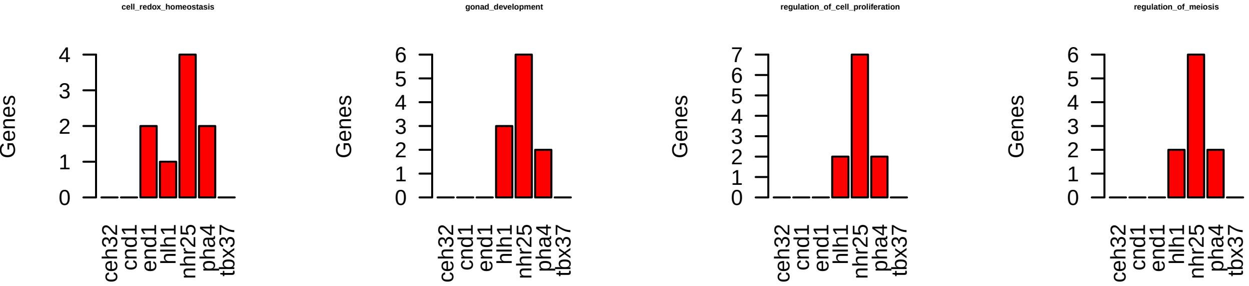
cell_redox_homeostasis
gonad_development
regulation_of_cell_proliferation
regulation_of_meiosis
Genes
0
1
2
3
4
5
6
7
ceh32
cnd1
end1
hlh1
nhr25
pha4
tbx37
