# Supplementary material for: The C. elegans embryonic transcriptome with tissue, time, and alternative splicing resolution
Source: Genome Res. 2019 Jun;29(6):1036–45. doi: 10.1101/gr.243394.118 (PMC6581053; doi:10.1101/gr.243394.118)
Supplement: Supplemental Material [file supp_gr.243394.118_Supplemental_File_S1.zip › biological_process.mixed.pdf]

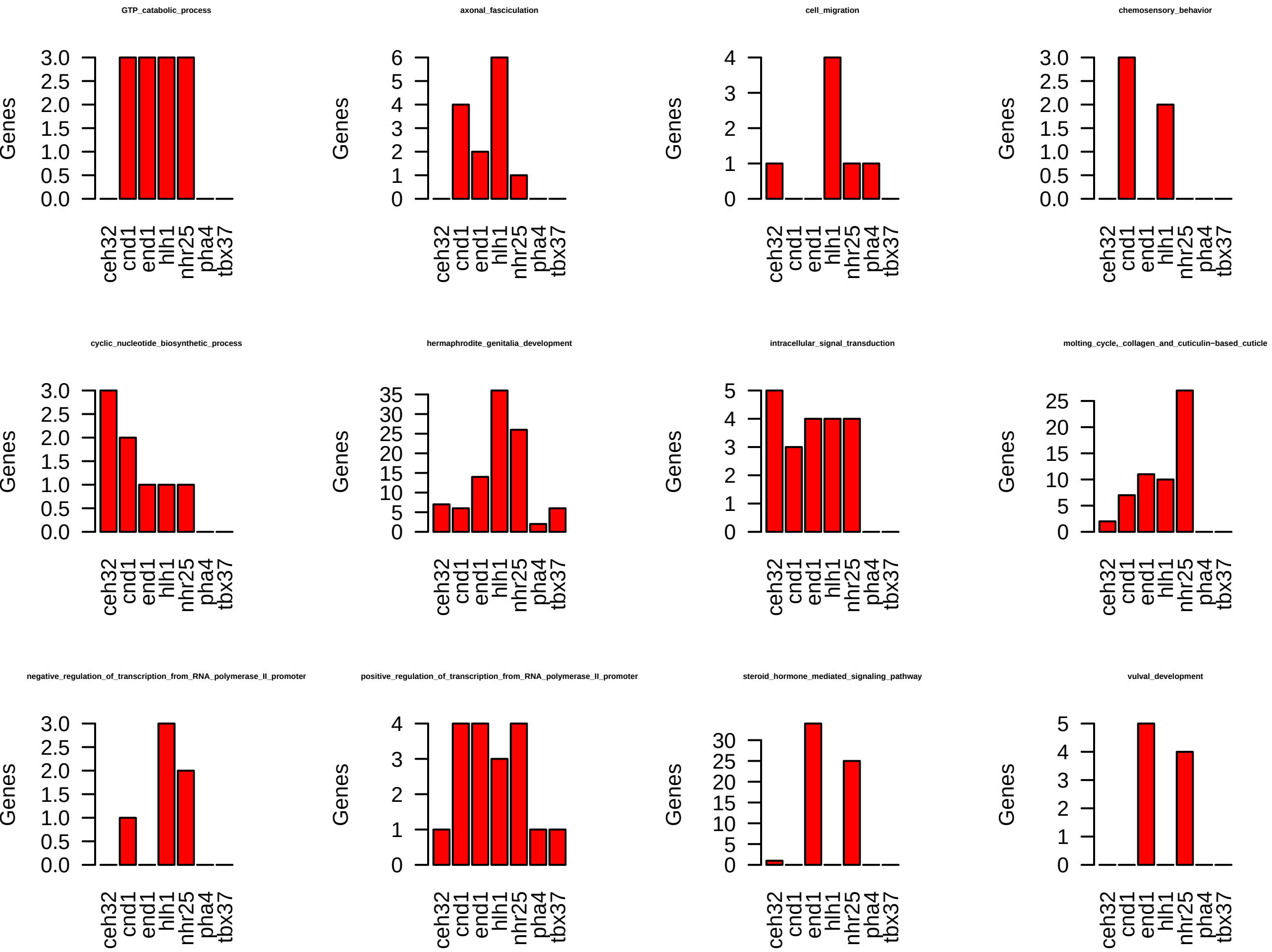
GTP_catabolic_process
axonal_fasciculation
cell_migration
chemosensory_behavior
cyclic_nucleotide_biosynthetic_process
hermaphrodite_genitalia_development
intracellular_signal_transduction
molting_cycle,_collagen_and_cuticulin−based_cuticle
negative_regulation_of_transcription_from_RNA_polymerase_II_promoter
positive_regulation_of_transcription_from_RNA_polymerase_II_promoter
steroid_hormone_mediated_signaling_pathway
vulval_development
Genes
ceh32
cnd1
end1
hlh1
nhr25
pha4
tbx37
