# Supplementary material for: The C. elegans embryonic transcriptome with tissue, time, and alternative splicing resolution
Source: Genome Res. 2019 Jun;29(6):1036–45. doi: 10.1101/gr.243394.118 (PMC6581053; doi:10.1101/gr.243394.118)
Supplement: Supplemental Material [file supp_gr.243394.118_Supplemental_File_S1.zip › cellular_component.end1.pdf]

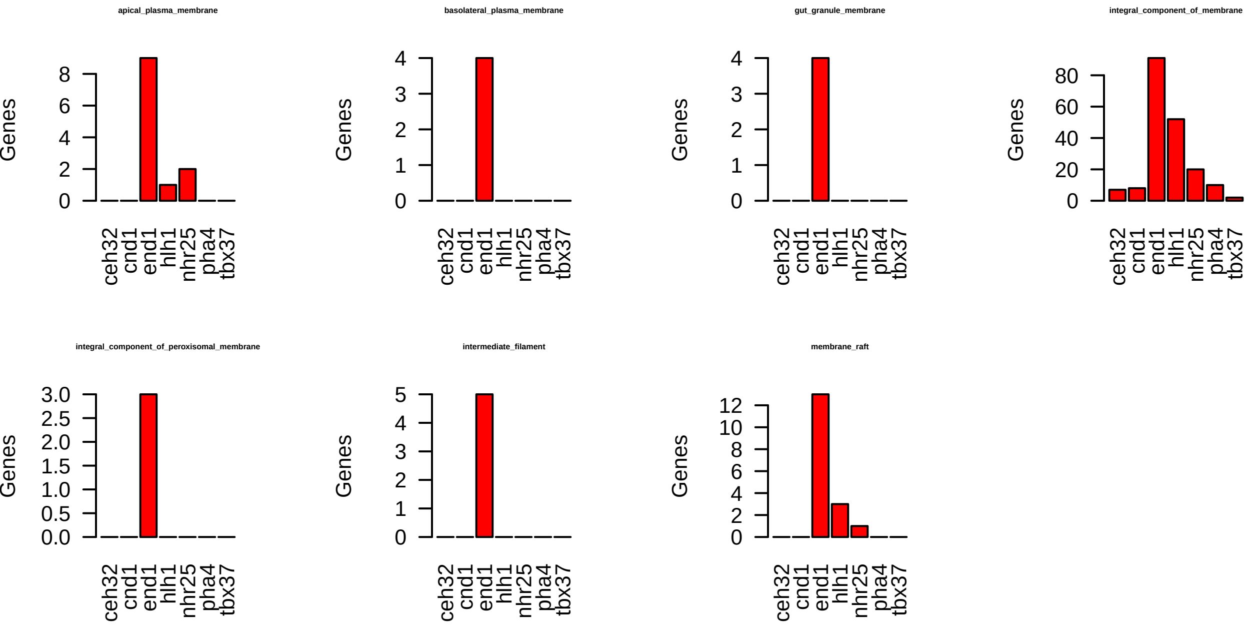
apical_plasma_membrane
basolateral_plasma_membrane
gut_granule_membrane
integral_component_of_membrane
integral_component_of_peroxisomal_membrane
intermediate_filament
membrane_raft
Genes
ceh32
cnd1
end1
hlh1
nhr25
pha4
tbx37
0
2
4
6
8
1
3
20
40
60
80
0.0
0.5
1.0
1.5
2.0
2.5
3.0
5
10
12
