# Supplementary material for: The C. elegans embryonic transcriptome with tissue, time, and alternative splicing resolution
Source: Genome Res. 2019 Jun;29(6):1036–45. doi: 10.1101/gr.243394.118 (PMC6581053; doi:10.1101/gr.243394.118)
Supplement: Supplemental Material [file supp_gr.243394.118_Supplemental_File_S1.zip › cellular_component.hlh1.pdf]

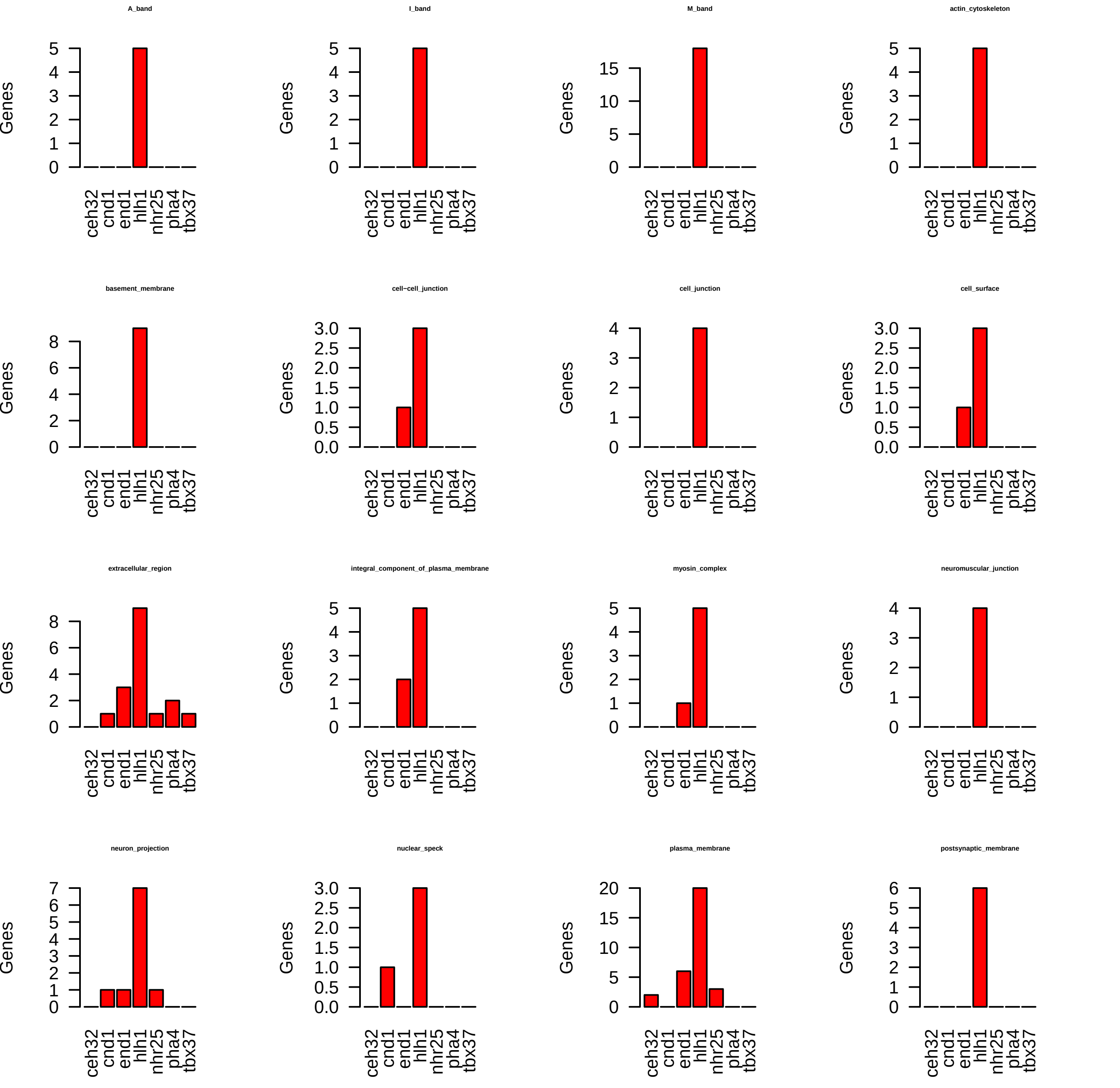
A_band
I_band
M_band
actin_cytoskeleton
basement_membrane
cell−cell_junction
cell_junction
cell_surface
extracellular_region
integral_component_of_plasma_membrane
myosin_complex
neuromuscular_junction
neuron_projection
nuclear_speck
plasma_membrane
postsynaptic_membrane
Genes
ceh32
cnd1
end1
hlh1
nhr25
pha4
tbx37

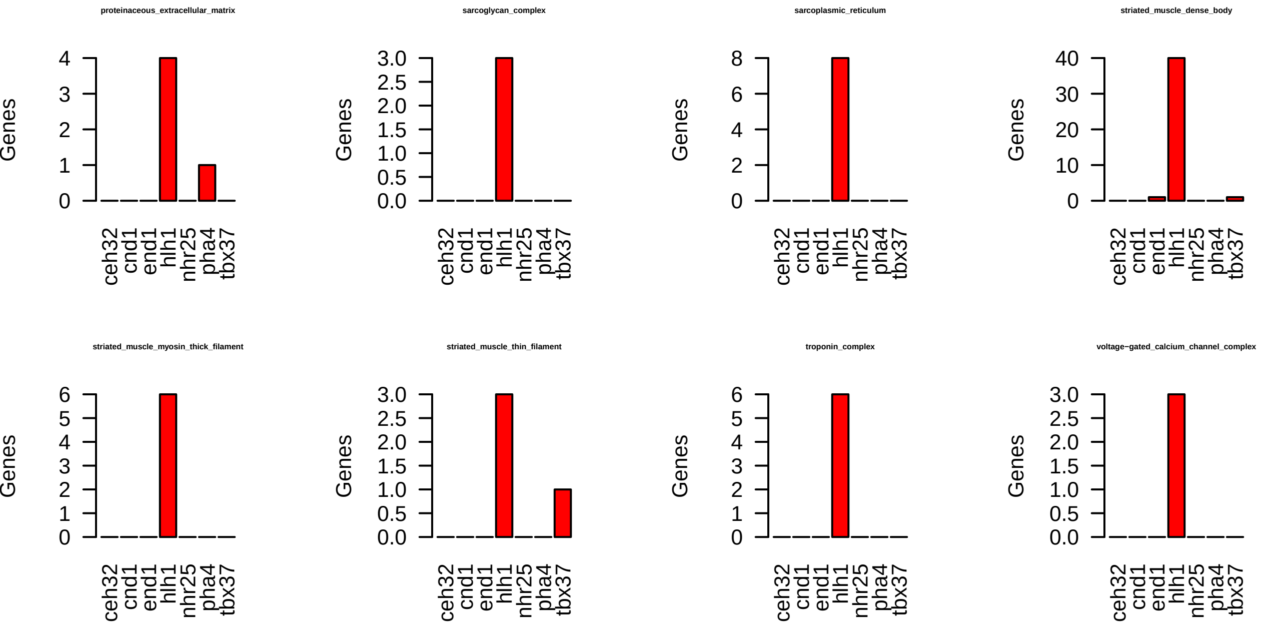
proteinaceous_extracellular_matrix
sarcoglycan_complex
sarcoplasmic_reticulum
striated_muscle_dense_body
striated_muscle_myosin_thick_filament
striated_muscle_thin_filament
troponin_complex
voltage−gated_calcium_channel_complex
Genes
ceh32
cnd1
end1
hlh1
nhr25
pha4
tbx37
