# Supplementary material for: The C. elegans embryonic transcriptome with tissue, time, and alternative splicing resolution
Source: Genome Res. 2019 Jun;29(6):1036–45. doi: 10.1101/gr.243394.118 (PMC6581053; doi:10.1101/gr.243394.118)
Supplement: Supplemental Material [file supp_gr.243394.118_Supplemental_File_S1.zip › molecular_function.end1_nhr25.pdf]

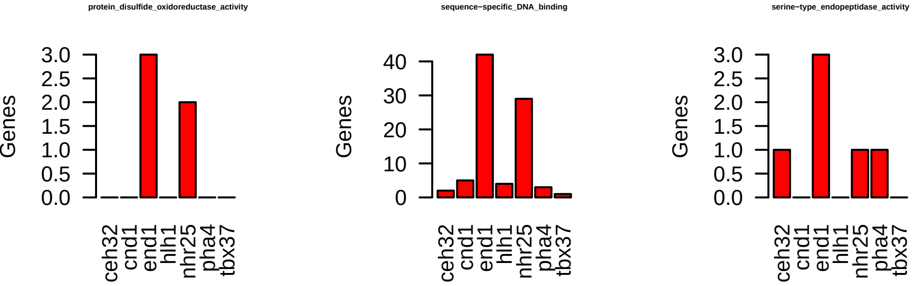
protein_disulfide_oxidoreductase_activity
sequence−specific_DNA_binding
serine−type_endopeptidase_activity
Genes
0.0
0.5
1.0
1.5
2.0
2.5
3.0
0
10
20
30
40
ceh32
cnd1
end1
hlh1
nhr25
pha4
tbx37
