# Supplementary material for: The C. elegans embryonic transcriptome with tissue, time, and alternative splicing resolution
Source: Genome Res. 2019 Jun;29(6):1036–45. doi: 10.1101/gr.243394.118 (PMC6581053; doi:10.1101/gr.243394.118)
Supplement: Supplemental Material [file supp_gr.243394.118_Supplemental_File_S1.zip › molecular_function.hlh1.pdf]

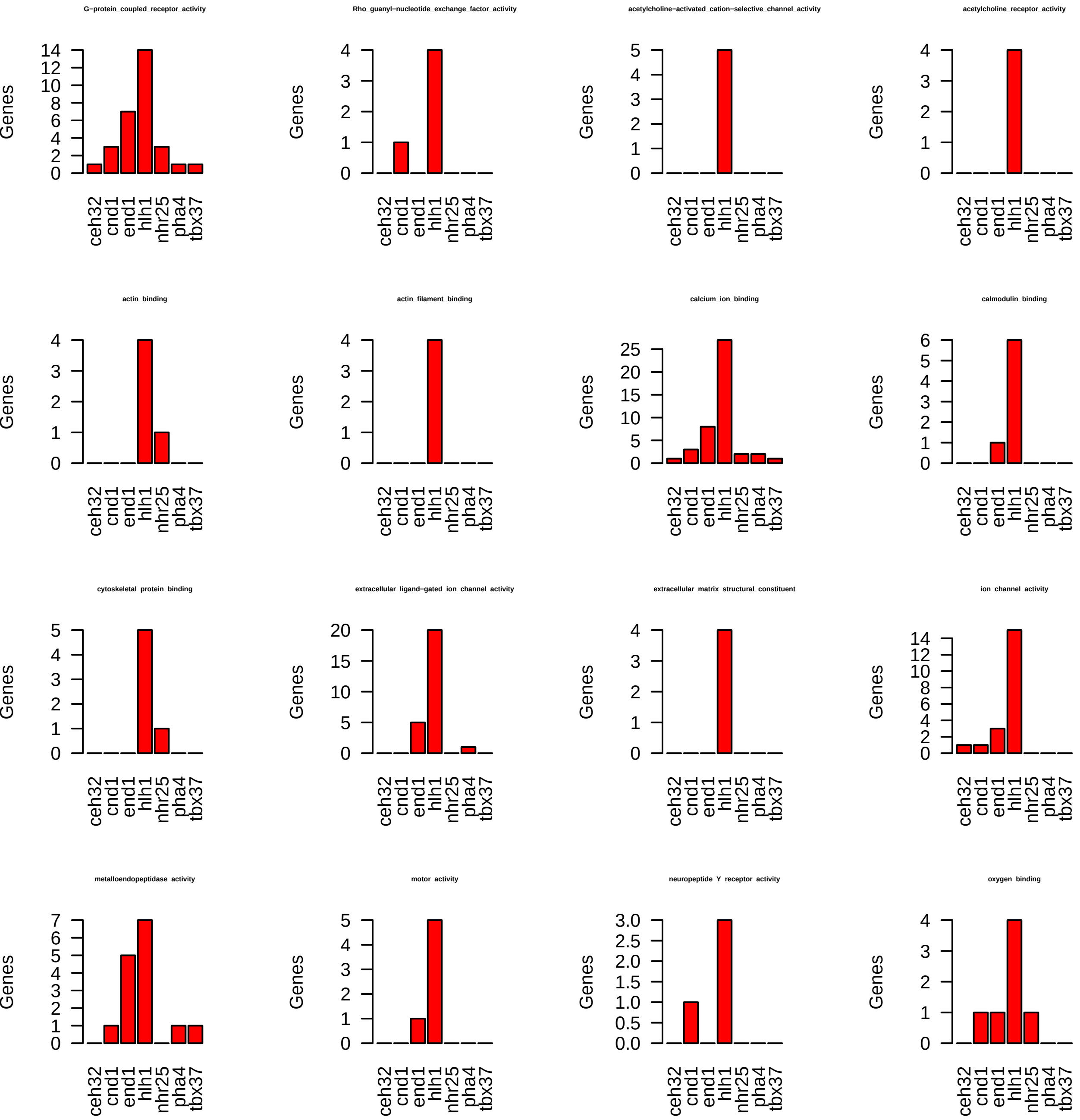
G−protein_coupled_receptor_activity
Rho_guanyl−nucleotide_exchange_factor_activity
acetylcholine−activated_cation−selective_channel_activity
acetylcholine_receptor_activity
actin_binding
actin_filament_binding
calcium_ion_binding
calmodulin_binding
cytoskeletal_protein_binding
extracellular_ligand−gated_ion_channel_activity
extracellular_matrix_structural_constituent
ion_channel_activity
metalloendopeptidase_activity
motor_activity
neuropeptide_Y_receptor_activity
oxygen_binding
Genes
ceh32
cnd1
end1
hlh1
nhr25
pha4
tbx37

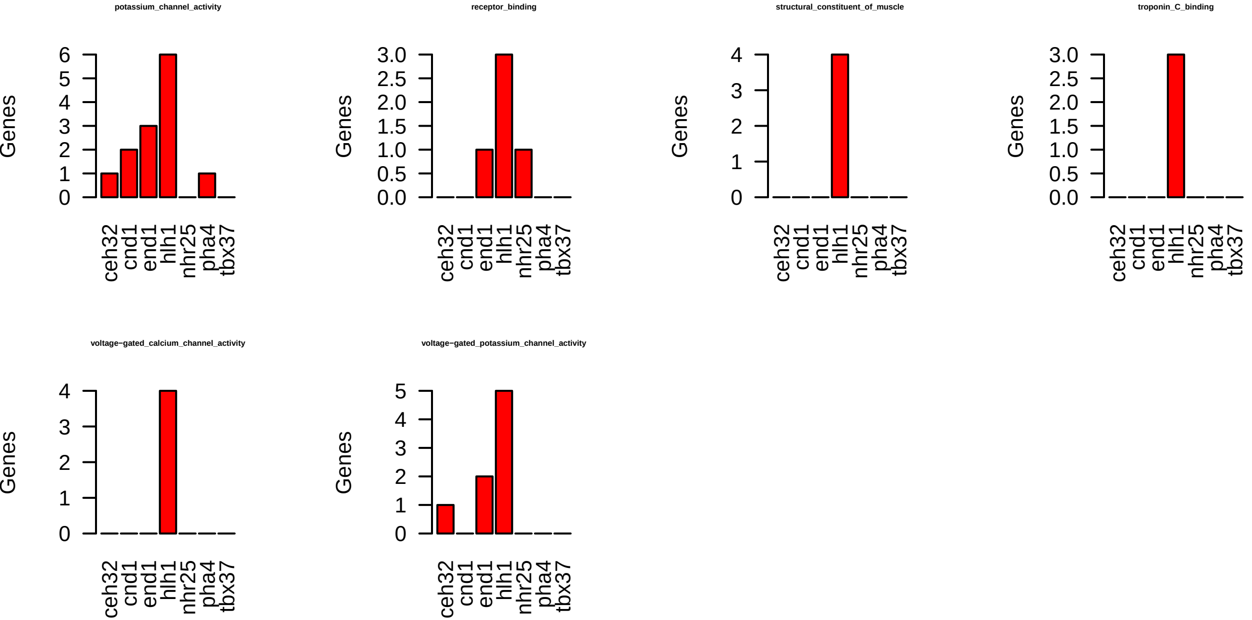
potassium_channel_activity
receptor_binding
structural_constituent_of_muscle
troponin_C_binding
voltage−gated_calcium_channel_activity
voltage−gated_potassium_channel_activity
Genes
ceh32
cnd1
end1
hlh1
nhr25
pha4
tbx37
