# Supplementary figures and images for: The C. elegans embryonic transcriptome with tissue, time, and alternative splicing resolution
Source: Genome Res. 2019 Jun;29(6):1036–45. doi: 10.1101/gr.243394.118 (PMC6581053; doi:10.1101/gr.243394.118)

axon\_guidance

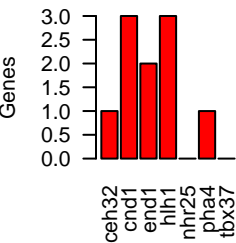

dauer\_larval\_development

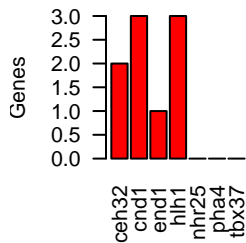

regulation\_of\_cell\_migration

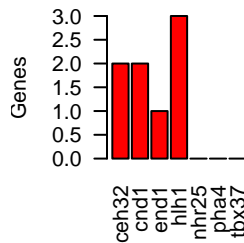

Supplement: Supplemental Material [file supp_gr.243394.118_Supplemental_File_S1.zip › biological_process.ceh32_cnd1_end1_hlh1.pdf]

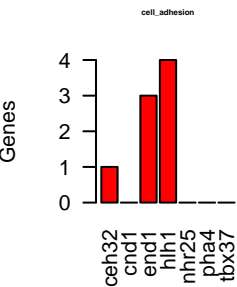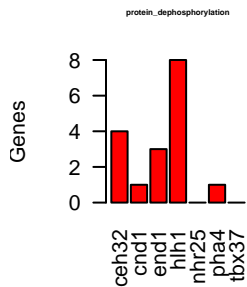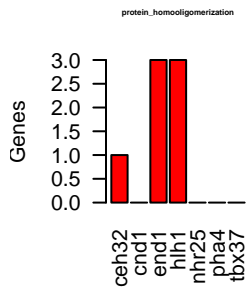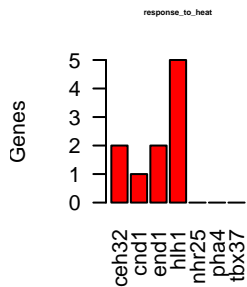

Supplement: Supplemental Material [file supp_gr.243394.118_Supplemental_File_S1.zip › biological_process.ceh32_end1_hlh1.pdf]

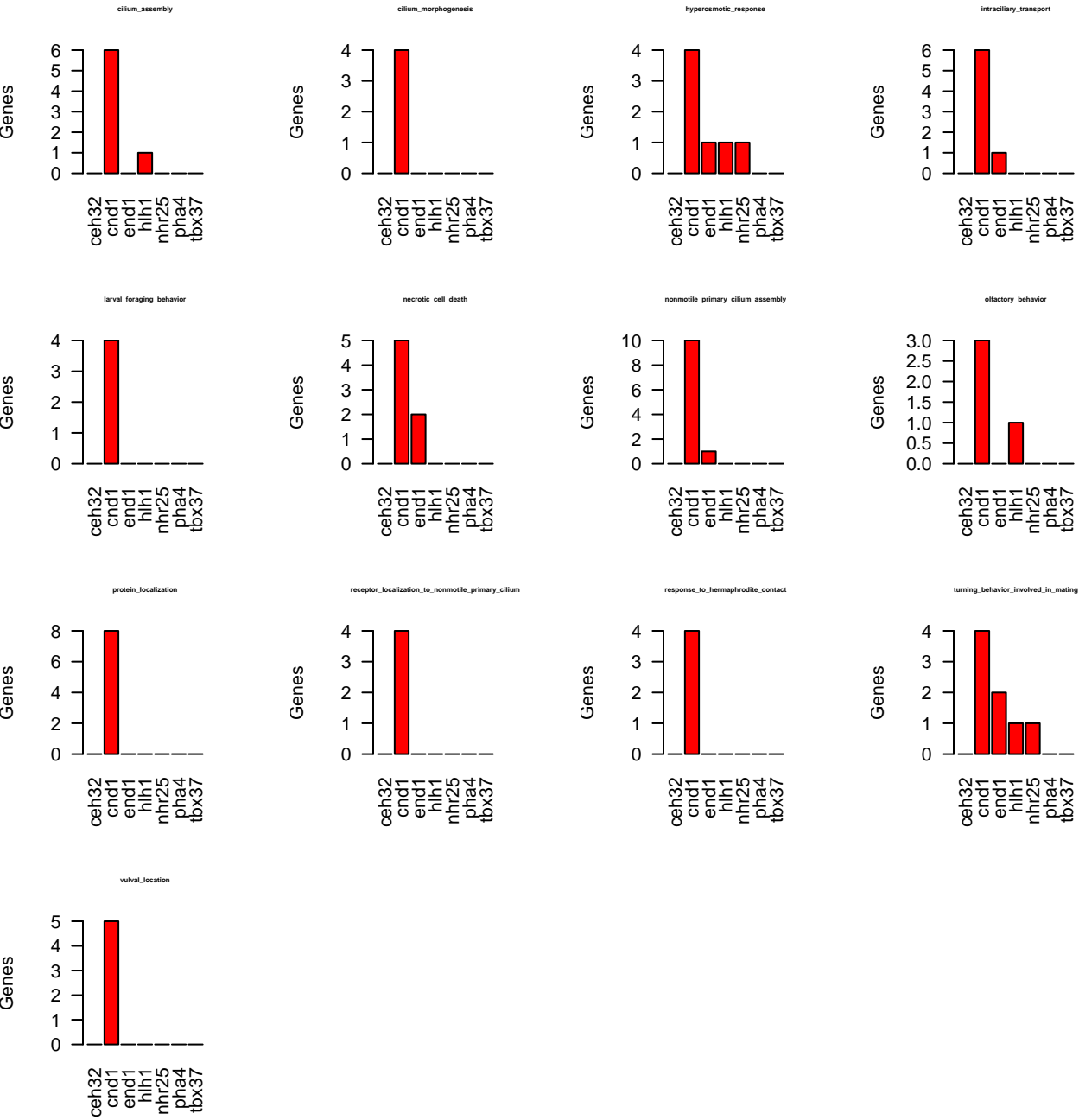

Supplement: Supplemental Material [file supp_gr.243394.118_Supplemental_File_S1.zip › biological_process.cnd1.pdf]

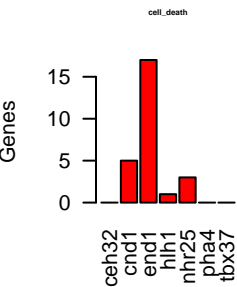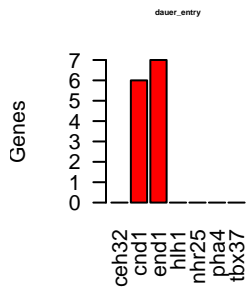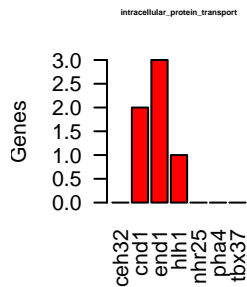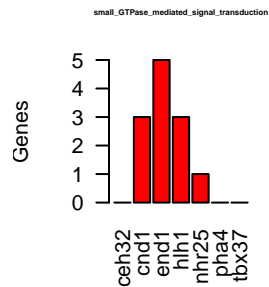

Supplement: Supplemental Material [file supp_gr.243394.118_Supplemental_File_S1.zip › biological_process.cnd1_end1.pdf]

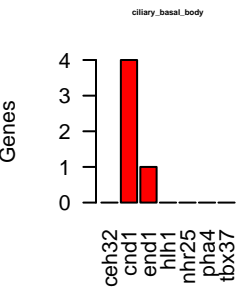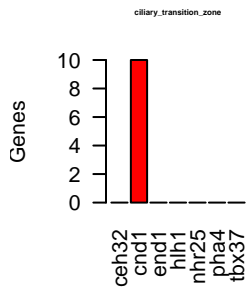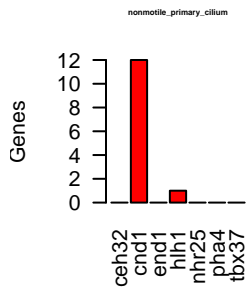

Supplement: Supplemental Material [file supp_gr.243394.118_Supplemental_File_S1.zip › cellular_component.cnd1.pdf]

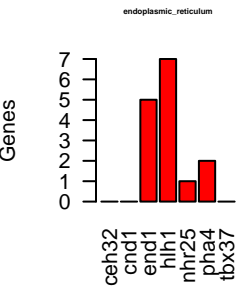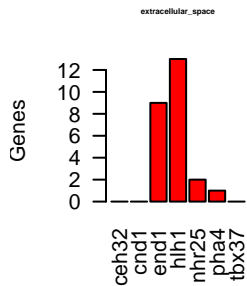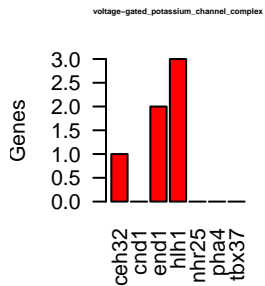

Supplement: Supplemental Material [file supp_gr.243394.118_Supplemental_File_S1.zip › cellular_component.end1_hlh1.pdf]

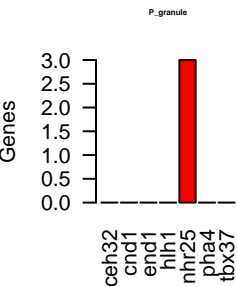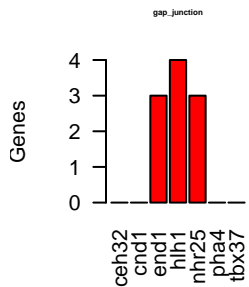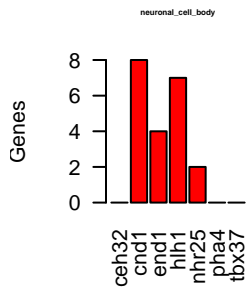

Supplement: Supplemental Material [file supp_gr.243394.118_Supplemental_File_S1.zip › cellular_component.nhr25.pdf]

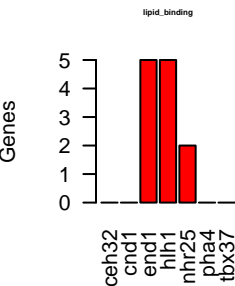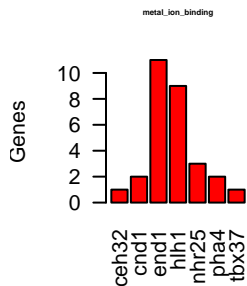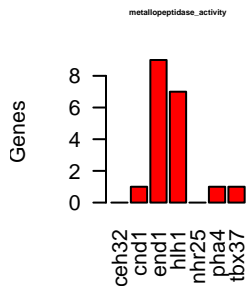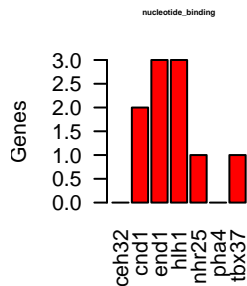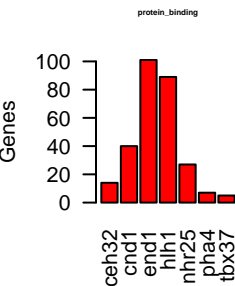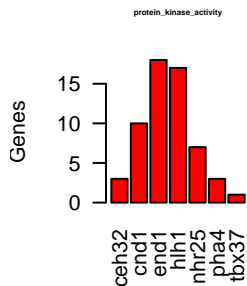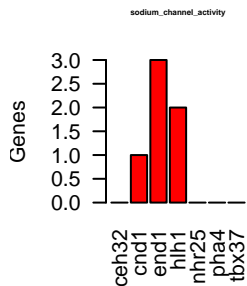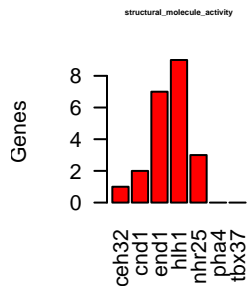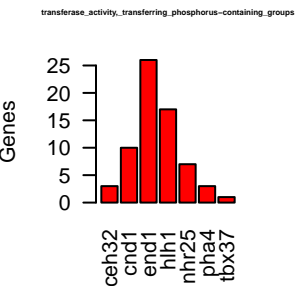

Supplement: Supplemental Material [file supp_gr.243394.118_Supplemental_File_S1.zip › molecular_function.cnd1_end1_hlh1.pdf]

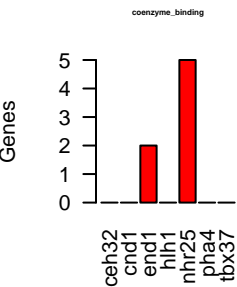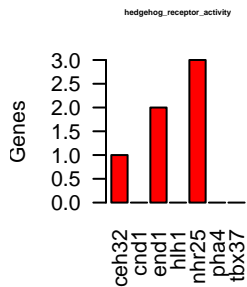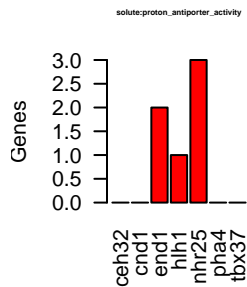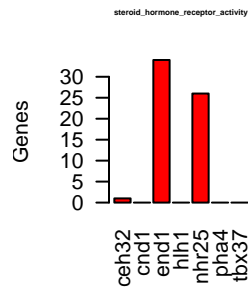

Supplement: Supplemental Material [file supp_gr.243394.118_Supplemental_File_S1.zip › molecular_function.cnd1_end1_nhr25.pdf]

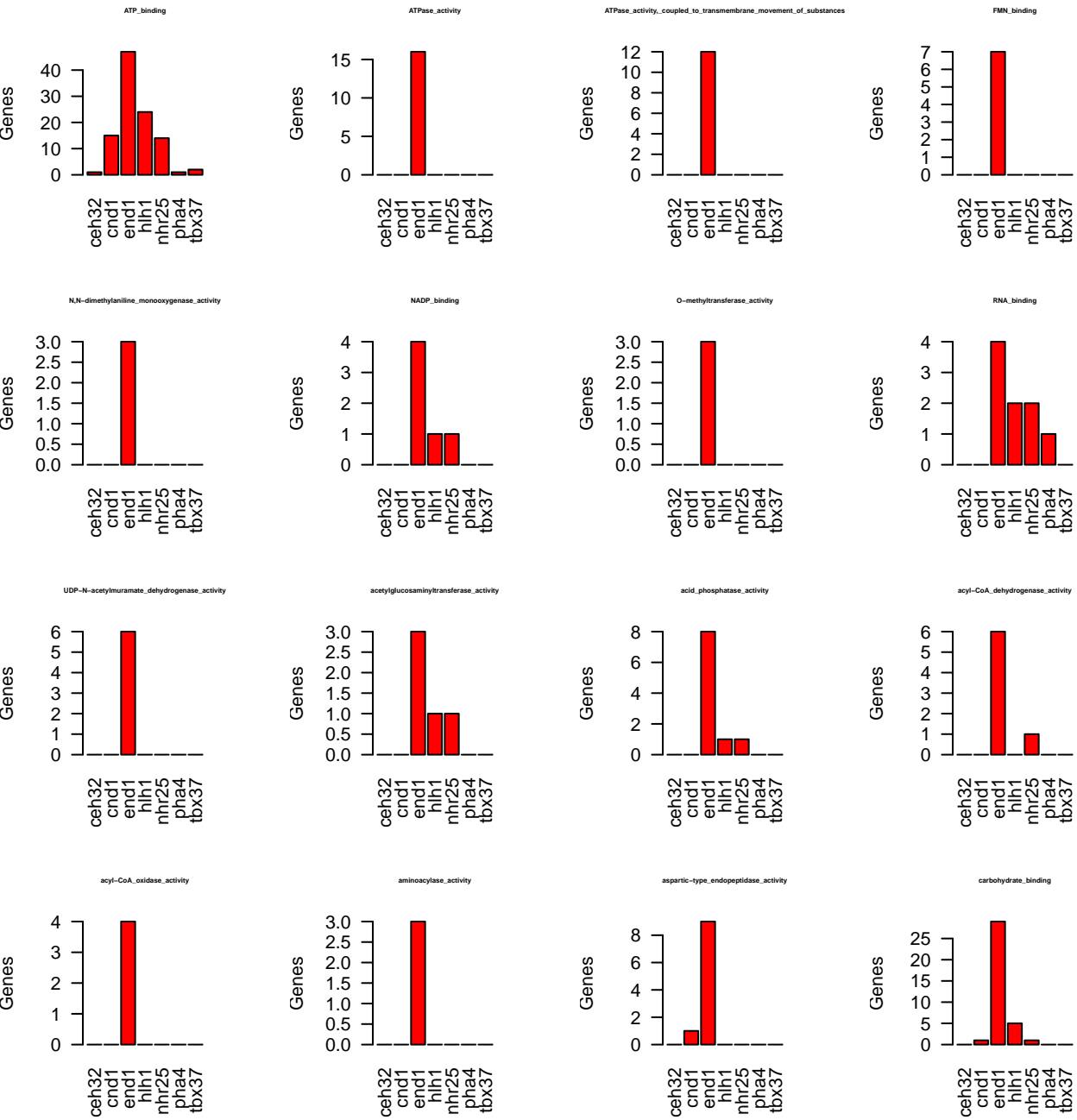

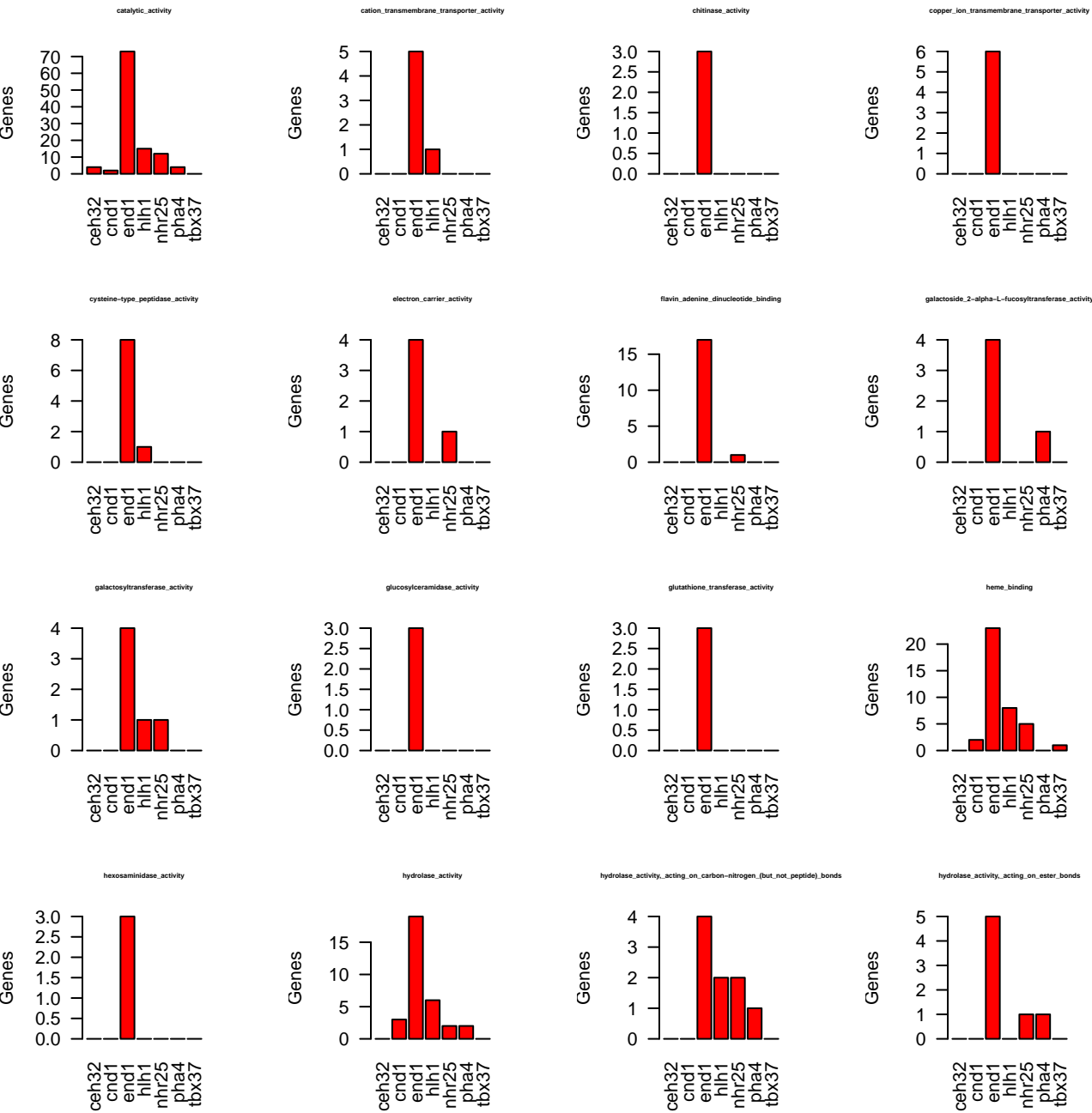

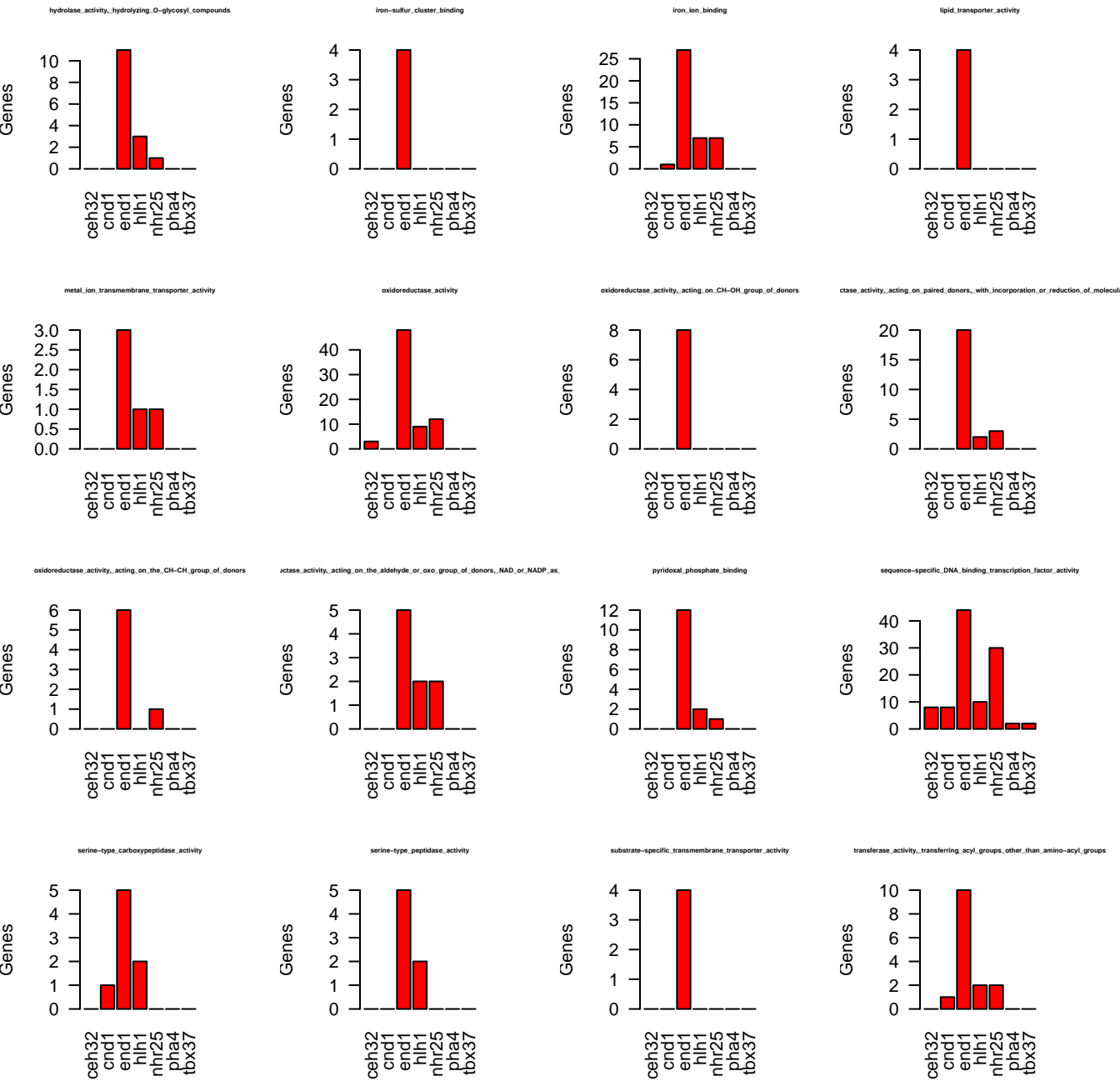

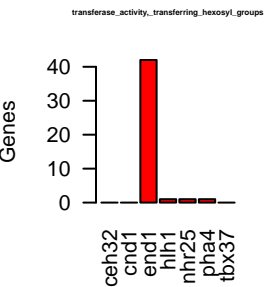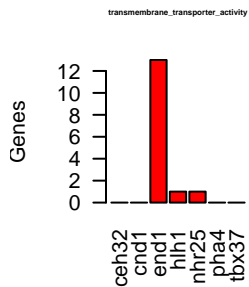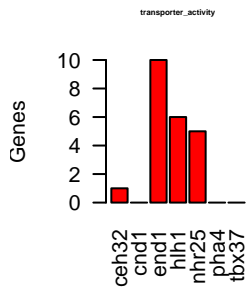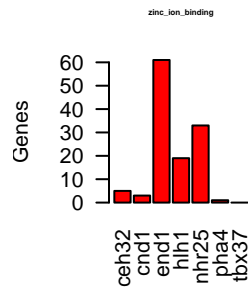

Supplement: Supplemental Material [file supp_gr.243394.118_Supplemental_File_S1.zip › molecular_function.end1.pdf]

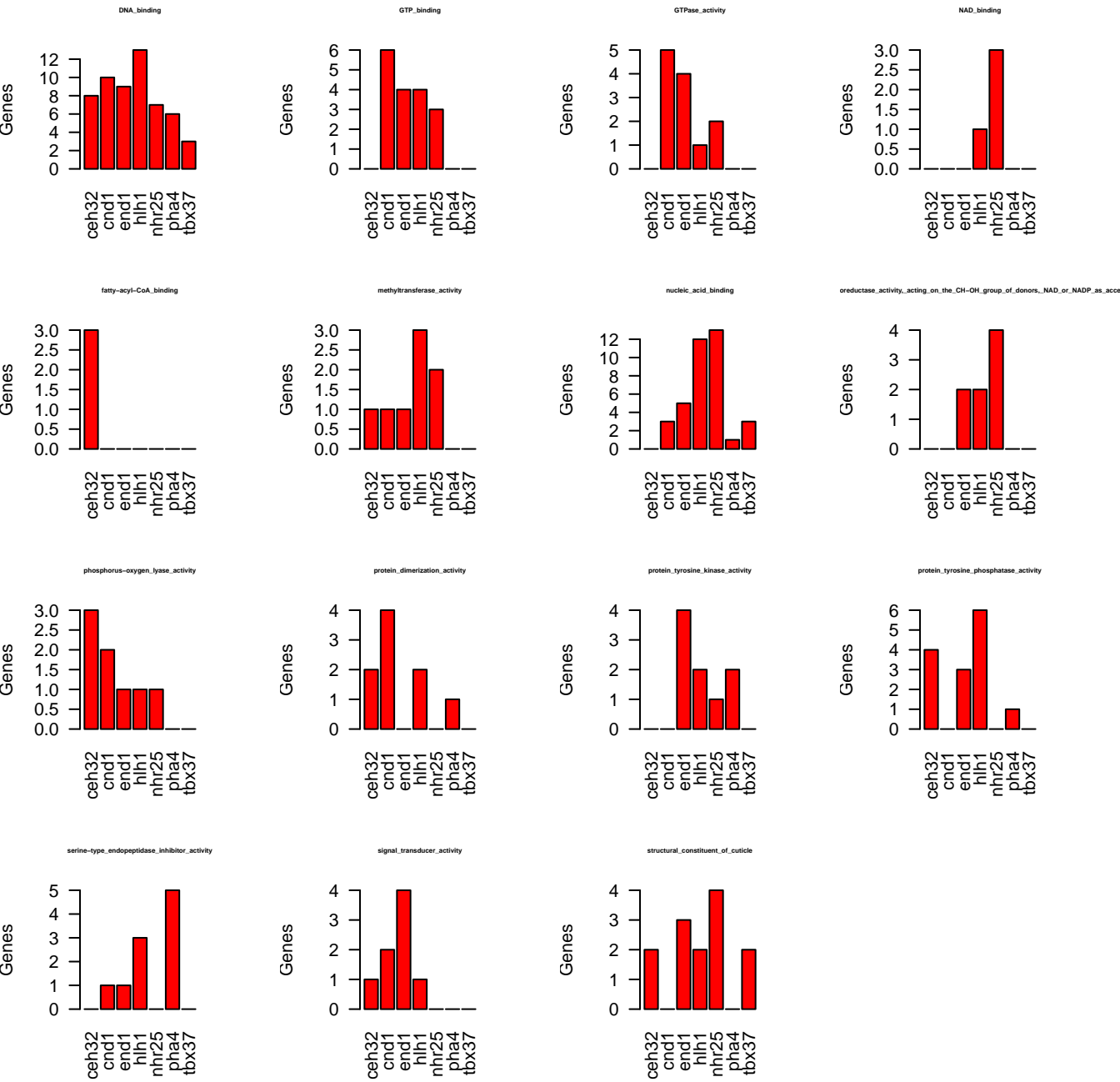

Supplement: Supplemental Material [file supp_gr.243394.118_Supplemental_File_S1.zip › molecular_function.mixed.pdf]
