# Supplementary material for: The C. elegans embryonic transcriptome with tissue, time, and alternative splicing resolution
Source: Genome Res. 2019 Jun;29(6):1036–45. doi: 10.1101/gr.243394.118 (PMC6581053; doi:10.1101/gr.243394.118)
Supplement: Supplemental Material [file supp_gr.243394.118_Supplemental_Methods_.docx]

**SUPPLEMENTAL METHODS**

**Strains used**

The strains used in this study are as follows: RW10461: stIs10461(*ceh-32p*::H1-Wcherry;*unc-119*(+));*unc-119*(*ed3*), RW10055: stIs10055(*cnd-1*::H1-Wcherry;*unc-119*(+)); *unc-119(ed3*), RW20408: stIs10220(*end-1*::H1-Wcherry;*unc-119*(+)); stIs10389(*pha-4*::GFP::3×FLAG;*unc-119*(+)); *unc-119(ed3),* RW12148: stIs10318(*nhr-25*::GFP::3×FLAG;*unc-119*(+)); stIs10220(*end-1*::mCherry);*unc-119*(+)); *unc-119(ed3),* RW12160: stIs10088(*hlh-1*::H1-Wcherry;*unc-119*(+)); *unc-119(ed3)*, and RW10499: stIs10499(*tbx-37*::H1-Wcherry;*unc-119*(+)); *unc-119(ed3).*

**Embryo synchronization and isolation**

The strains used were developmentally synchronized in order to obtain as tight an interval of developmental age as possible for each time point. First, worms were grown on eight 150 mm peptone rich NGM plates seeded with NA22 bacteria. When the mixed population reached saturation on the plates, the worms were bleached using the standard KOH/sodium hypochlorite treatment (50 mL total 40 mL water, 5 mL 12 % bleach, 5 mL 5M KOH). Isolated embryos were left to hatch in egg buffer (Edgar 1995), shaking for 16 hours at 20^o^C. Hatched L1 worms were then plated onto 16 150 mm peptone-rich NA22 plates at a density of 70,000 worms per plate. After ~54 hours at 20^o^C, the worms were egg prepped, and left shaking for 16 hours at 20^o^C. Hatched L1 worms were then plated onto 32 peptone rich NA22 plates (150 mm) at a density of 70,000 worms per plate. Lastly after ~54 hours at 20^o^C, the worms were egg prepped, including a sucrose float for embryo purification (5 ml of embryos/carcasses in egg buffer and 5 ml of 60% sucrose mixed in a 15 mL falcon tube and spun for 3 minutes at 200 g). The floating embryos were then removed from the sucrose via 1 mL pipette tip, and washed 3× with room temperature egg buffer and re-suspended in 2 mL egg buffer. To degrade the eggshell, 1 U/mL chitinase (C6137 Sigma) was added to the embryos at a ratio of 1 mL chitinase to 0.5 mL embryo suspension. The embryo chitinase suspension was then transferred to 30 mm petri dishes (1-2 dishes per time point), adding no more than 1 mL of embryo suspension to each. The degradation of the eggshell was monitored via dissecting microscope (~20 min. for the eggshell to degrade), and post degradation all dishes were topped up to a final volume of 1.5 mL with egg buffer. To dissociate the embryos into single cells, 100 μl of 15 mg/ml pronase (P6911 Sigma) was added to ~1.5 mL of embryo/egg buffer suspension. The cell suspension was drawn from the dish using a 3 cc syringe and used to wash the dish to isolate all embryos. Embryos were repeatedly pulled through a 21 gauge needle 20×, in a 1.5 mL Eppendorf tube, then incubated at room temperature for 5 minutes. Lastly, the cell suspension was drawn repeatedly through a 21 gauge needle until a suspension of single cells was confirmed via microscope.

**Embryo cell counts including fixation**

After isolation of embryos post-alkaline bleach treatment, 50 μl of embryo suspension from the initial time point of each time series was fixed using methanol/formaldehyde fixation and stored at -20^o^C. Embryos were sampled by viewing a small volume of the fixed embryo suspension using a Zeiss Axioplan microscope and counting the number of cells in each embryo. More than 100 embryos were scored for each time point analyzed to obtain a measure of embryo age and synchronization.

**Cell sorting**

Cells were transferred to a 5 mL FACS tube and kept chilled during sorting. FACS sorting was carried out on a FACS ARIA III using a 561 laser for mCherry detection and a 488 laser for GFP detection. Sustained events were kept to below 3000 sustained events per second to maximize accuracy and efficiency. In order to properly gate fluorescence, N2 embryos were dissociated into single cells and passed through the FACS to set minimum thresholds for fluorescence detection. 900 μl of TRIzol LS (ThermoFisher) was preloaded into a 1.5 mL Eppendorf tube and cells were sorted directly into the TRIzol LS. 150,000 cells were sorted into each tube that gave a sorted volume of 300 μl and maintained a ratio of 3:1 for TRIzol LS to sample. At regular intervals the sorting was paused and cells/TRIzol LS were vortexed together to avoid a layer of cell suspension forming on top of the TRIzol LS. Upon completion of cell sorting, the TRIzol LS and cell mixture was vortexed for 10 seconds, and flash frozen on dry ice. While both fluorescent and non-fluorescent cells were isolated, only the fluorescent (cells expressing the transcription factor) were used in most of our analysis as they contain the most diverse and specific sets of cell types. The RNA-seq data for non-fluorescent cells were used solely for identifying artifacts during identification of RNA editing changes.

**RNA isolation**

Total RNA was isolated by adding 240 μl of chloroform to the 900 μl TRIzol + 300 μl cell volume, and shaken vigorously for 15 seconds. The total volume was then added to heavy gel phase lock tubes (Prime 5), allowed to settle for five minutes, and then spun at 12,000 g for 15 minutes. After spinning, the aqueous layer was removed and added to an equivalent volume of 100 % EtOH and mixed via pipetting. This volume was then passed through a Direct-Zol RNA purification tube (Zymo Research) and purified using the standard protocol.

**rRNA depletion and cDNA production**

rRNA was reduced in each sample using Ribo-Zero (Illumina) using 2 μl of both the rRNA reaction buffer and rRNA removal solution (¼ recommended amounts) in 20 μl of reaction volume (½ the recommended reaction volume), and using 112 μl magnetic beads (½ of the recommended beads). After rRNA reduction, remaining RNA was purified using SPRI beads (Agencourt RNAClean XP). Immediately following the RNA cleanup, first strand cDNA synthesis was carried out using SuperScript IV (Invitrogen), followed by second strand synthesis using the second strand synthesis module (NEB). Qubit DNA HS reagents were used to quantify cDNA after synthesis.

**cDNA library preparation and sequencing**

In order to obtain DNA of the appropriate size for sequencing, shearing was carried out using a Covaris LE220. The entire cDNA reaction (~90 μl) was transferred to an assembled Covaris microtube and sheared using the following settings: duty factor 22%, peak incident power 450W, cycles/burst 200, treatment time 90 s, temperature 7°C, water level 6, and sample volume 90 μl. Sheared DNA was repaired using the NEB end repair module using standard amounts in 90 μl total volume, and A-tailed cDNA was produced in a 50 μl reaction containing 5 μl 10× PCR buffer II (no MgCl2), 5 μl MgCl2 (100 mM), 0.2 μl dATP (100 mM), and 0.5 μl Amplitaq (Applied Biosystems N8080161). Lastly, the following Y sequencing adapter was generated by a slow anneal (ACACTCTTTCCCTACACGACGCTCTTCCGATC*T, /5Phos/GATCGGAAGAGCGGTTCAGCAGGAATGCCGAG) and then ligated to the cDNA in a 50 μl reaction using 5 μl Ultrapure T4 ligase Mix (Enzymatics L603-HC-L), 5 μl Ligase buffer (10×), 0.5 μl Y-adapter (50 μM) at 25°C for 15 minutes. To barcode each sample, QPCR with Solexa barcodes was carried out using KAPA HiFi Hotstart PCR mix and an anneal temperature of 65°C. In total, 12-16 cycles of amplification were used to reach the log phase of PCR. To size select fragments for sequencing, samples were run on a 6% TBE polyacrylamide gel, and barcoded DNA between 350 bp and 650 bp was excised and purified using 0.2 μm gel filtration tubes (Nanosep, 29300-642) followed by Ampure XP bead cleanup. After pooling samples to 2 nM concentration, sequencing was carried out using Illumina HiSeq 2500 and NextSeq 500 instruments to obtain 50 bp paired end reads or 75 bp paired end reads respectively. Data was demultiplexed by barcode into individual sample FASTQ files using bcl2fastq.

**Sequence alignment and gene expression calculations**

Reads were aligned with STAR version 2.4.2a (Dobin et al. 2013) to the WS245 genome sequence from WormBase (<ftp://ftp.wormbase.org/pub/wormbase/species/c_elegans/sequence/genomic/c_elegans.PRJNA13758.WS245.genomic.fa.gz>)

with a maximum intron size of 25,000 (Supplemental_Table_S19.xlsx). Transcript-level expression was calculated using BitSeq (Glaus et al. 2012). BitSeq output is relative abundance of transcripts' fragments (RPKM), and these values were used to calculate TPM for the transcripts (Supplemental_Table_S20.txt, Supplemental_Table_S21.txt). The conversion from RPKM to TPM is straightforward (https://www.rna-seqblog.com/rpkm-fpkm-and-tpm-clearly-explained/).

Transcripts were defined by the WS245 annotation file from WormBase (<ftp://ftp.wormbase.org/pub/wormbase/species/c_elegans/gff/c_elegans.PRJNA13758.WS245.annotations.gff3.gz>). Histones, ncRNA and rRNA were not included in the set of transcripts for which expression was calculated. The sum of transcript expression for all transcripts defined for the gene was deemed gene level expression. The TPM values were averaged for the replicates of each point for each different tissue to obtain a TPM for each tissue. To determine the saturation of our sequencing depth, we used the program subSeq (Robinson and Storey, 2014). This program subsamples the gene count array and runs DESeq2 on the subsampled count arrays, comparing the results. We used subSeq to sample several pairs of series at several time points and obtained a consistent result suggesting that the depth of sequencing for each sample is approaching saturation but not over saturated (Supplemental_Fig_S19.pdf).

**PCR duplicate removal**

To identify PCR duplicated fragments from our read counts, alignments were read and those flagged by the alignment software as chimeric or alternate were skipped. Read starts were counted at each chromosome base after adjusting the alignment starts for clipping and insertion/deletion events. A chromosome base with one or more read starts was called *occupied*. Chromosome regions with relatively highly occupied base frequency were called *high coverage regions*. High coverage regions were found using the d-segment dynamic programming algorithm (http://bozeman.mbt.washington.edu/compbio/mbt599/Lecture5.pdf) to scan chromosomes for maximal scoring segments where the algorithm uses the sum of positive scores for occupied bases and negative scores for unoccupied bases. For the purpose of evaluating the high coverage regions, we find four sets of high coverage regions where the sets differ by the target minimum occupied base frequency. The target frequencies and corresponding d-segment parameters are 0.01, 0.1, 1.0 and 10.0 occupied bases per chromosome base, the occupied base scores are 500, 50, 5, and 2, respectively, the drop-off, D, values are -500, -50, -5, and -2, respectively, and the minimum ratios of occupied to total chromosome bases are .01, .1, 1.0, and 10.0, respectively. The unoccupied base score is -1 and the minimum segment length is 10. Regions identified by the d-segment algorithm as having >= 0.1 occupied bases per chromosome base were flagged as high coverage for these paired end read data. Regions not flagged as high coverage were considered to be low coverage regions. We assume that finding by chance two or more paired-end read pairs with the same starts and ends in low coverage regions is near zero so we consider such read pairs to be PCR duplicates. Incidentally, we found that estimating PCR duplicate rates from single-end read data using this method is unreliable because of substantially increased likelihood of seeing chance duplicates. We counted the number of reads and duplicate reads in all low coverage regions with length >= 200 bases and recorded the estimated PCR duplicate rate as the ratio of the two counts. We select for removal observed duplicates that reflect PCR duplication as follows. Treating each high coverage region independently, we counted the number of read starts. The estimated number of PCR duplicate reads is found using this count and the estimated PCR duplicate rate. The corresponding number of observed duplicate read pairs are chosen randomly for removal from the observed duplicates in the region, and all duplicate read pairs were removed in low coverage regions.

We sought to find good PCR duplicate rate estimates by choosing a threshold value for identifying genome regions with a high rate of occupied bases substantially below the one at which the duplicate rate estimate stabilized.

We checked the PCR duplicate removal accuracy by comparing gene read counts before and after PCR duplicate removal. We selected four replicates with estimated duplicate rates from 0.09 to 0.65 and made BAM alignment files before and after duplicate removal. We counted the reads in the annotated genes using HT-Seq count, and compared the before and after counts for each gene using the formula

$$rdiff=\left( c2-c1*\left( 1-r \right) \right)/c1*\left( 1-r \right)$$

where *c1* and c*2* are the read counts for a gene before and after duplicate removal, respectively, *r* is the estimated duplicate rate, and *rdiff* is the relative difference between the measured and calculated gene read counts after duplicate removal. We tabulated *rdiff* values of genes with at least five reads after duplicate removal and summarize the results in Supplemental_Methods_Table_S1. The relatively small fractions of genes with *rdiff* < -0.20 and *rdiff* > 0.20 shows that the duplicate removal has a minimal effect on the gene read count profiles, even with data sets that have large PCR duplicate rates. Full code for this application is available at https://github.com/gevirl/seldup.

**Assessment of density divergences among samples**

Any gene which did not have TPM expression above 14.0 in at least one of the 85 samples was excluded from this analysis and replicate samples were averaged. The density of genes vs the ln(TPM) was calculated using a Kernel Density Estimator with default parameters. This was done for each time of each series. The divergence of these density functions was estimated using the Kullback-Leibler Divergence. Within a series, time T0 was compared to all the other times. For each time (T0 to T4), all the series were compared pairwise. To get a baseline expectation of the range of the Kullback-Leibler divergence values, this divergence was calculated for all the possible pairings of the replicate samples (not averaged) and the distribution of those divergences was plotted.

**Conservation of differentially expressed exons in *C. briggsae***

Using the list of *C. briggsae/C. elegans* orthologs obtained from WormBase (c_briggsae.PRJNA10731.WS245.orthologs.txt.gz), 30,371 exons that were not differentially expressed were found to be a part of a gene that did not have an ortholog in *C. briggsae*. There were 90,174 exons that were not differentially expressed and were part of a gene that did have an ortholog in *C. briggsae*. There were 5,998 and 34,987 exons that were differentially expressed and were part of a gene that did not or did have an ortholog in *C. briggsae*, respectively. To test whether the difference was significant, using a Z score for two population proportions, a value of p < 0.0001 was obtained with the result being significant at p<0.01 (both when using a one-tailed or using a two-tailed hypothesis).

**Supplemental_Methods_Table_S1:** rdiff values for genes post-PCR duplicate removal

| **Replicate** | **Estimated PCR duplicate rate** | **Fraction of genes with at least five counts after duplicate removal** | **Cumulative fraction of genes with *rdiff* < -0.20** | **Cumulative fraction of genes with *rdiff* < 0.20** |
| --- | --- | --- | --- | --- |
| hlh-1 T0 rep2 | 0.09 | 0.42 | 0.006 | 1.000 |
| hlh-1 T2 rep2 | 0.23 | 0.40 | 0.013 | 0.980 |
| hlh-1 T0 rep1 | 0.57 | 0.45 | 0.028 | 0.964 |
| hlh-1 T1 rep1 | 0.65 | 0.48 | 0.036 | 0.959 |

**SL identification**

Reads were aligned to the WS245 genome using STAR (version STAR_2.4.2a) (--alignIntronMax 25000 --outFilterMultimapNmax 100 --winAnchorMultimapNmax 100) and PCR duplicates were removed as described above. Only reads with the flag (column 2 in the SAM file) less than 256 and with at least 3 aligned reads across all samples were retained. Reads containing portions of untemplated (not aligned to the genome) splice leader sequence (SL1 through SL14) on the 5' end of the read were identified. If only 6 or 7 bases of the SL were identified, at least 3 reads were required to call the SL confirmed. If 8 to 10 bases of the SL were identified, only 2 reads were required. Only 1 read is required if there are 11 or more bases of the SL identified. Further, only SLs were kept that had at least three reads between all samples combined. For detecting differences in raw counts of SL1 versus SL2 at different positions or at the same positions, the Barnard test was used on 2×2 contingency tables with a one-sided P-value cutoff of 1e-05. Further, we required that the differential usage occur in at least two timepoints for the same tissue pair in order to consider it significant.

**Intron identification**

Reads were aligned to the WS245 genome using STAR (version STAR_2.4.2a) (--alignIntronMax 25000 --outFilterMultimapNmax 100 --winAnchorMultimapNmax 100) and PCR duplicates were removed as described above. Intron-spanning reads were retained if the flag column, column 2 in the SAM file, had a value less than 256 (meaning it is a primary alignment), the NH flag set to 1, and at least three aligned reads total (including all samples) confirming that intron. For introns that fell within annotated genes (WormBase WS245) or within 500 bases up or downstream of those genes, reads covering each intron had to exceed 1% of the average number of reads aligning to all of the annotated introns within that gene for the intron to be considered valid. Finally, novel introns (those not overlapping WormBase-annotated introns and not overlapping those from published embryonic data (Boeck et al. 2016) were clustered to identify clusters of errant splice junctions falling within 50 bases of one another. If more than 10 introns were located within 50 bases of one another, they were considered misalignments and removed from the set.

**Differential exon and splice junction usage analysis using JunctionSeq**

We used QoRTs and JunctionSeq software to look for evidence of alternative splicing. After PCR duplicate removal, the BAM files from the STAR aligner were filtered to remove reads aligned to introns. QoRTs version 1.1.8 was run to count alignments in the tissue sample BAM files using the command line parameters 'QC --runFunctions writeKnownSplices,writeNovelSplices,writeSpliceExon,writeDESeq'. and run again using the command line parameters 'mergeNovelSplices --minCount 6' to merge the novel splice junction annotations and counts. JunctionSeq was run on the resulting read count files using the functions runJunctionSeqAnalyses() with the parameters 'analysis.type="junctionsAndExons", method.dispFinal="shrink", optimizeFilteringForAlpha=0.1, use.multigene.aggregates=TRUE' and writeCompleteResults() with the parameter 'FDR.threshold=0.1'. An exon was defined as tissue-specific (Supplemental_Table_S9.txt, Supplemental_S10.txt) when it was significantly (padjust <0.1 and log_2_FC>=1.0) higher in all valid pairwise comparisons (see Supplementary_Table_S1.doc). The neuronal (*cnd-1)* T0 data, for example, were compared to T0 samples for intestine (*end-1),* hypodermis *(nhr-25),* body wall muscle *(hlh-1)* and pharynx (*pha-4)*.  When, in all four pairwise comparisons, an exon in the *cnd-1* sample was significantly (padjust <0.1 and log_2_FC>=1.0) “higher” than the other four tissues, it was counted as being specific to *cnd-1* T0.  Similarly, if an exon was significantly (padjust<0.1 and log_2_FC<=-1.0) “lower” in all valid pairwise, it would be counted in the “lower” exons (Supplemental_Table_S8.doc). Through examination of the coverage data, it was determined that the 5’ and 3’ ends of the *ceh-32* samples had uneven coverage. Therefore, *ceh-32* exons were not included in the selection of tissue-specific exons.

**RNA editing**

WormBase annotation (<http://www.wormbase.org> (Harris et al. 2014); c_elegans.PRJNA13758.WS245.annotations.gff2.gz) was used to identify all curated coding exons. The following bases were removed: a) any base that overlapped pseudogenes, UTRs, introns, or other ncRNA exons, b) runs of 5 or more of the same nucleotide, c) annotated repeats and transposons, d) sites where SNVs cannot be called from Thompson et al., (2013), and e) WormBase annotated SNPs.

Comparisons to the RNA editing data in Zhao et al. (2015) were performed by converting Zhao’s reported WS230 coordinates to WS245 and using mpileup (-q 20 (mapping quality), -Q 25 (base quality)) to count the number of A, C, G and Ts at each position.

To identify putative RNA edits across all data sets for this project, samtools mpileup was used (Li et al. 2009) (minimum mapping quality 20, minimum base quality 25) to process the alignment files of uniquely aligned reads with PCR duplicates removed. After mpileup, vcftutils (Li et al. 2009) was run set to require a maximum read depth of 500, a minimum read depth of 10, and at least 3 reads confirming the alternate base. Another filter required the percentage of reads with the alternate call to be at least 5%. Any possible edit was also required to be in at least two time points (although those time points could be in different samples). Because many of the differences could be related to strain differences, possible RNA edits were called using the same parameters in the “non” samples. Any RNA edits appearing in any of the “non” samples were removed from consideration. Further, because there were not “non” samples for the *pha-4* and *end-1* samples, and because they were from the same strain, for any RNA edit occurring in both the *pha-4* and *end-1*, those were also removed from consideration.

**Gene coregulatory region analysis**

The 11,408 genes used for gene expression cluster analysis were used in this analysis as well. Adjacent pairs of genes in the set were identified and classified as head-to-head, head-to-tail, or tail-to-tail, and a set of 10,000 pairs of random genes on the same chromosome was selected as a comparison group. If both genes of the pair were in the same operon, they were excluded from the analysis. TPM expression for the each gene was normalized across samples to a maximum of 1, and the distance between the two genes of each pair was calculated as the expression distance of the normalized TPM. A Student’s *t*-test was used to assess the significance in the difference of the mean of the expression distances between each classification of gene pairs.

**Transcription factor regulation analysis**

ChIP-seq peaks were obtained from the modERN (Kudron et al. 2018) and modENCODE (Araya et al. 2014) projects using ChIP-seq experiments for embryonic and early larval (L1, L2, L3) stages for analysis. Peaks were clustered by grouping adjacent peaks (if they were not separated by more than 60 bases), and the target gene of each cluster was selected using the following procedure. The peak locations of all the peaks in the cluster were averaged to give a single peak location for the cluster, with intragenic peak locations assigned to the gene the peak is within. Peaks between two adjacent mRNA genes on the same strand were assigned to the most 5 prime end adjacent gene, and peaks falling between two genes on opposite strands were assigned to the most proximal gene.
The probability that a target gene would have a transcription factor in its associated cluster was calculated for each transcription factor across the genome. This probability was simply the ratio of the number of target genes that had the transcription factor in its associated ChIP-seq peak cluster divided by the total number of target genes. This ratio is used to compare to the same ratio for sets of differentially expressed genes in each tissue and time. The null hypothesis is that there was no difference between the ratios. A binomial distribution is used to determine a p-value for rejecting the null hypothesis. The probability of success (p) for the binomial is the probability calculated for all the target genes in the genome. The number of trials (n) is the number of genes in the DE set. The number of successes (k) for the binomial is the number of genes in the DE set that contain the TF. If k < np the pVal is the cdf(k) , if k > np then the p value is 1.0 - cdf(k) (cdf is the cumulative distribution function).

**Supplemental Methods References**

Edgar LG. 1995. Chapter 13: blastomere culture and analysis. In *Caenorhabditis elegans*: modern biological analysis of an organism (ed. Epstein HF, Shakes DC), Vol. 48, pp. 303–321. Academic Press. Cambridge, Massachusetts. doi:10 .1016/s0091-679x(08)61393-x

Harris TW, Baran J, Bieri T, Cabunoc A, Chan J, Chen WJ, Davis P, Done J, Grove C, Howe K, et al. 2014. WormBase 2014: new views of curated biology. Nucleic Acids Res **42**: D789–D793. doi:10.1093/nar/gkt1063

Li H, Handsaker B, Wysoker A, Fennell T, Ruan J, Homer N, Marth G, Abecasis G, Durbin R; 1000 Genome Project Data Processing Subgroup. 2009. The Sequence Alignment/Map format and SAMtools. Bioinformatics **25**: 2078–2079. doi:10.1093/bioinformatics/btp352

Thompson OA, Snoek LB, Nijveen H, Sterken MG, Volkers RJ, Brenchley R, Van’t Hof A, Bevers RP, Cossins AR, Yanai I, et al. 2015. Remarkably diver- gent regions punctuate the genome assembly of the Caenorhabditis elegans Hawaiian strain CB4856. Genetics **200**: 975–989. doi:10.1534/ genetics.115.175950
