# Supplementary material for: The C. elegans embryonic transcriptome with tissue, time, and alternative splicing resolution
Source: Genome Res. 2019 Jun;29(6):1036–45. doi: 10.1101/gr.243394.118 (PMC6581053; doi:10.1101/gr.243394.118)
Supplement: Supplemental Material [file supp_gr.243394.118_Supplemental_Table_S3.doc]

Supplemental_Table_S3: Differential gene expression changing tissue specificity over time

| Gene | Timepoint | TPM ratio: highest/second highest | DESeq2 padjust | Highest Sample | Second Highest Sample | Highest TPM | Second Highest TPM | DESeq2 baseMean expression | DESeq2 log2FC |
| --- | --- | --- | --- | --- | --- | --- | --- | --- | --- |
| WBGene00000174_C32C4.2_aqp-6 | T0 | 8.76 | 0 | cnd1_T0 | pha4_T0 | 63.18 | 7.21 | 76.62 | 18.95 |
| WBGene00000174_C32C4.2_aqp-6 | T1 | 11.01 | 0 | ceh32_T1 | nhr25_T1 | 71.46 | 6.49 | 76.62 | 7.12 |
|  |  |  |  |  |  |  |  |  |  |
| WBGene00000661_Y81G3A.5_col-86 | T0 | 7.64 | 0 | tbx37_T0 | hlh1_T0 | 20.54 | 2.69 | 21.46 | 8.73 |
| WBGene00000661_Y81G3A.5_col-86 | T1 | 7.73 | 0 | tbx37_T1 | end1_T1 | 18.21 | 2.36 | 21.46 | 8.61 |
| WBGene00000661_Y81G3A.5_col-86 | T2 | 8.41 | 0.01 | ceh32_T2 | pha4_T2 | 16.75 | 1.99 | 21.46 | 11.32 |
| WBGene00000661_Y81G3A.5_col-86 | T3 | 10.58 | 0.02 | tbx37_T3 | nhr25_T3 | 11.87 | 1.12 | 21.46 | 8.39 |
|  |  |  |  |  |  |  |  |  |  |
| WBGene00000998_K07E12.1_dig-1 | T0 | 17.47 | 0 | end1_T0 | hlh1_T0 | 22.37 | 1.28 | 4250.33 | 17.47 |
| WBGene00000998_K07E12.1_dig-1 | T4 | 15.8 | 0 | hlh1_T4 | end1_T4 | 174.9 | 11.07 | 4250.33 | 18.2 |
|  |  |  |  |  |  |  |  |  |  |
| WBGene00001960_DY3.3_hlh-16 | T0 | 88.45 | 0 | cnd1_T0 | hlh1_T0 | 2203.15 | 24.91 | 669.07 | 62.17 |
| WBGene00001960_DY3.3_hlh-16 | T1 | 108.52 | 0 | cnd1_T1 | end1_T1 | 2377.97 | 21.91 | 669.07 | 61.19 |
| WBGene00001960_DY3.3_hlh-16 | T2 | 58.57 | 0 | cnd1_T2 | end1_T2 | 1712 | 29.23 | 669.07 | 32.27 |
| WBGene00001960_DY3.3_hlh-16 | T3 | 33.23 | 0 | ceh32_T3 | nhr25_T3 | 784.02 | 23.6 | 669.07 | 16.24 |
| WBGene00001960_DY3.3_hlh-16 | T4 | 16.04 | 0.06 | ceh32_T4 | nhr25_T4 | 712.75 | 44.45 | 669.07 | 9.15 |
|  |  |  |  |  |  |  |  |  |  |
| WBGene00002085_ZK75.2_ins-2 | T3 | 16.54 | 0 | cnd1_T3 | pha4_T3 | 473.86 | 28.65 | 336.02 | 13.28 |
| WBGene00002085_ZK75.2_ins-2 | T4 | 8.07 | 0.09 | ceh32_T4 | pha4_T4 | 121.86 | 15.11 | 336.02 | 7.69 |
|  |  |  |  |  |  |  |  |  |  |
| WBGene00002086_ZK75.3_ins-3 | T0 | 12.94 | 0.01 | cnd1_T0 | hlh1_T0 | 14.65 | 1.13 | 15.95 | 11.45 |
| WBGene00002086_ZK75.3_ins-3 | T1 | 11.62 | 0.02 | cnd1_T1 | pha4_T1 | 26.39 | 2.27 | 15.95 | 14.41 |
| WBGene00002086_ZK75.3_ins-3 | T2 | 20.66 | 0 | cnd1_T2 | pha4_T2 | 31.71 | 1.54 | 15.95 | 37.36 |
| WBGene00002086_ZK75.3_ins-3 | T3 | 13.84 | 0.01 | cnd1_T3 | pha4_T3 | 23.86 | 1.72 | 15.95 | 16.93 |
| WBGene00002086_ZK75.3_ins-3 | T4 | 11.44 | 0.04 | ceh32_T4 | pha4_T4 | 13.01 | 1.14 | 15.95 | 14.44 |
|  |  |  |  |  |  |  |  |  |  |
| WBGene00003377_C39E6.4_mls-2 | T0 | 11.58 | 0.05 | tbx37_T0 | nhr25_T0 | 148.86 | 12.86 | 506.7 | 8.87 |
| WBGene00003377_C39E6.4_mls-2 | T2 | 10.14 | 0.04 | ceh32_T2 | pha4_T2 | 220.52 | 21.75 | 506.7 | 10.18 |
|  |  |  |  |  |  |  |  |  |  |
| WBGene00003909_F45B8.4_pag-3 | T0 | 14.45 | 0.03 | cnd1_T0 | hlh1_T0 | 11.65 | 0.81 | 160.29 | 14.83 |
| WBGene00003909_F45B8.4_pag-3 | T1 | 24.32 | 0 | ceh32_T1 | hlh1_T1 | 29.28 | 1.2 | 160.29 | 40.26 |
| WBGene00003909_F45B8.4_pag-3 | T2 | 9.99 | 0.02 | ceh32_T2 | hlh1_T2 | 43.43 | 4.35 | 160.29 | 13.42 |
| WBGene00003909_F45B8.4_pag-3 | T3 | 11.56 | 0.09 | tbx37_T3 | hlh1_T3 | 48.58 | 4.2 | 160.29 | 8.19 |
|  |  |  |  |  |  |  |  |  |  |
| WBGene00004353_F45B8.2_rgs-10 | T2 | 6.92 | 0.1 | ceh32_T2 | pha4_T2 | 6.45 | 0.93 | 8.74 | 9.92 |
| WBGene00004353_F45B8.2_rgs-10 | T3 | 8.12 | 0.1 | tbx37_T3 | end1_T3 | 3.28 | 0.4 | 8.74 | 7.24 |
|  |  |  |  |  |  |  |  |  |  |
| WBGene00004949_K08A8.2_sox-2 | T0 | 14.68 | 0.04 | cnd1_T0 | pha4_T0 | 473.92 | 32.28 | 1732.99 | 11.46 |
| WBGene00004949_K08A8.2_sox-2 | T1 | 8.61 | 0.1 | ceh32_T1 | pha4_T1 | 578.8 | 67.23 | 1732.99 | 9.6 |
|  |  |  |  |  |  |  |  |  |  |
| WBGene00004963_C17D12.6_spe-9 | T1 | 7.04 | 0.05 | cnd1_T1 | pha4_T1 | 3.1 | 0.44 | 10.14 | 17.56 |
| WBGene00004963_C17D12.6_spe-9 | T2 | 8.57 | 0.07 | ceh32_T2 | end1_T2 | 4.12 | 0.48 | 10.14 | 9.62 |
| WBGene00004963_C17D12.6_spe-9 | T3 | 9.88 | 0.09 | cnd1_T3 | hlh1_T3 | 4.31 | 0.44 | 10.14 | 6.46 |
|  |  |  |  |  |  |  |  |  |  |
| WBGene00006308_K09C4.8_sul-1 | T0 | 6.79 | 0 | tbx37_T0 | hlh1_T0 | 36.25 | 5.34 | 183.13 | 7.03 |
| WBGene00006308_K09C4.8_sul-1 | T4 | 4.04 | 0 | hlh1_T4 | cnd1_T4 | 25.87 | 6.4 | 183.13 | 7.22 |
|  |  |  |  |  |  |  |  |  |  |
| WBGene00006408_T14G12.3_tag-18 | T0 | 14.33 | 0.01 | tbx37_T0 | end1_T0 | 22.72 | 1.59 | 35.71 | 15.77 |
| WBGene00006408_T14G12.3_tag-18 | T1 | 9.44 | 0.04 | tbx37_T1 | end1_T1 | 18.67 | 1.98 | 35.71 | 11.76 |
| WBGene00006408_T14G12.3_tag-18 | T2 | 10.83 | 0.04 | tbx37_T2 | hlh1_T2 | 12.84 | 1.19 | 35.71 | 9.59 |
| WBGene00006408_T14G12.3_tag-18 | T4 | 26.6 | 0 | hlh1_T4 | pha4_T4 | 46.6 | 1.75 | 35.71 | 27.48 |
|  |  |  |  |  |  |  |  |  |  |
| WBGene00006778_F58E6.10_unc-42 | T0 | 10.76 | 0.01 | cnd1_T0 | hlh1_T0 | 74.57 | 6.93 | 318.22 | 20.06 |
| WBGene00006778_F58E6.10_unc-42 | T1 | 27.21 | 0.01 | cnd1_T1 | hlh1_T1 | 234.13 | 8.6 | 318.22 | 18.7 |
| WBGene00006778_F58E6.10_unc-42 | T4 | 24.15 | 0.02 | ceh32_T4 | hlh1_T4 | 656.49 | 27.19 | 318.22 | 14.98 |
|  |  |  |  |  |  |  |  |  |  |
| WBGene00007271_C03C10.5_C03C10.5 | T0 | 18.28 | 0 | cnd1_T0 | hlh1_T0 | 109.96 | 6.01 | 579.14 | 136.62 |
| WBGene00007271_C03C10.5_C03C10.5 | T1 | 95.12 | 0 | ceh32_T1 | pha4_T1 | 495.46 | 5.21 | 579.14 | 46.1 |
| WBGene00007271_C03C10.5_C03C10.5 | T2 | 18 | 0.08 | cnd1_T2 | end1_T2 | 494.7 | 27.48 | 579.14 | 10.39 |
| WBGene00007271_C03C10.5_C03C10.5 | T3 | 5 | 0 | cnd1_T3 | hlh1_T3 | 433.45 | 86.74 | 579.14 | 35.81 |
| WBGene00007271_C03C10.5_C03C10.5 | T4 | 19.35 | 0 | cnd1_T4 | hlh1_T4 | 473.83 | 24.49 | 579.14 | 60.68 |
|  |  |  |  |  |  |  |  |  |  |
| WBGene00007325_C05C9.1_C05C9.1 | T0 | 10.45 | 0.01 | hlh1_T0 | tbx37_T0 | 4.41 | 0.42 | 64.94 | 8.31 |
| WBGene00007325_C05C9.1_C05C9.1 | T1 | 4.43 | 0.08 | hlh1_T1 | pha4_T1 | 3.47 | 0.78 | 64.94 | 6.75 |
| WBGene00007325_C05C9.1_C05C9.1 | T3 | 9.05 | 0.03 | pha4_T3 | hlh1_T3 | 34.24 | 3.78 | 64.94 | 5.75 |
| WBGene00007325_C05C9.1_C05C9.1 | T4 | 11.71 | 0 | pha4_T4 | ceh32_T4 | 67.89 | 5.8 | 64.94 | 10.17 |
|  |  |  |  |  |  |  |  |  |  |
| WBGene00007863_C32C4.3_C32C4.3 | T0 | 10.56 | 0.01 | tbx37_T0 | nhr25_T0 | 35.84 | 3.39 | 101.51 | 7.54 |
| WBGene00007863_C32C4.3_C32C4.3 | T1 | 9.6 | 0.05 | ceh32_T1 | nhr25_T1 | 42.84 | 4.46 | 101.51 | 6.04 |
|  |  |  |  |  |  |  |  |  |  |
| WBGene00007938_C34F6.1_C34F6.1 | T0 | 12.23 | 0.02 | hlh1_T0 | pha4_T0 | 1.7 | 0.14 | 35.4 | 12.96 |
| WBGene00007938_C34F6.1_C34F6.1 | T4 | 18.28 | 0 | pha4_T4 | end1_T4 | 21.69 | 1.19 | 35.4 | 14.79 |
|  |  |  |  |  |  |  |  |  |  |
| WBGene00008066_C43D7.2_fbxb-65 | T2 | 9.5 | 0 | tbx37_T2 | end1_T2 | 59.62 | 6.27 | 104.53 | 9.44 |
| WBGene00008066_C43D7.2_fbxb-65 | T3 | 22 | 0 | cnd1_T3 | pha4_T3 | 28.99 | 1.32 | 104.53 | 18.82 |
|  |  |  |  |  |  |  |  |  |  |
| WBGene00008522_F02D8.5_F02D8.5 | T0 | 5.41 | 0.1 | tbx37_T0 | hlh1_T0 | 6.71 | 1.24 | 8.58 | 9.2 |
| WBGene00008522_F02D8.5_F02D8.5 | T1 | 3.18 | 0.06 | tbx37_T1 | hlh1_T1 | 5.15 | 1.62 | 8.58 | 9.85 |
| WBGene00008522_F02D8.5_F02D8.5 | T4 | 10.98 | 0.02 | cnd1_T4 | nhr25_T4 | 36.6 | 3.33 | 8.58 | 11.23 |
|  |  |  |  |  |  |  |  |  |  |
| WBGene00009014_F21D5.9_F21D5.9 | T0 | 51.72 | 0 | cnd1_T0 | hlh1_T0 | 245.7 | 4.75 | 211.32 | 44.32 |
| WBGene00009014_F21D5.9_F21D5.9 | T1 | 27.12 | 0 | ceh32_T1 | nhr25_T1 | 176.44 | 6.51 | 211.32 | 18.32 |
| WBGene00009014_F21D5.9_F21D5.9 | T2 | 20.86 | 0.01 | ceh32_T2 | nhr25_T2 | 197.2 | 9.46 | 211.32 | 12.86 |
|  |  |  |  |  |  |  |  |  |  |
| WBGene00010488_K02A11.3_K02A11.3 | T0 | 3.31 | 0.03 | nhr25_T0 | hlh1_T0 | 21.49 | 6.49 | 92.08 | 4.33 |
| WBGene00010488_K02A11.3_K02A11.3 | T4 | 3.43 | 0.02 | hlh1_T4 | nhr25_T4 | 59.09 | 17.23 | 92.08 | 4.31 |
|  |  |  |  |  |  |  |  |  |  |
| WBGene00011004_R04B5.6_R04B5.6 | T0 | 10.76 | 0 | end1_T0 | hlh1_T0 | 10.59 | 0.98 | 88.19 | 13.2 |
| WBGene00011004_R04B5.6_R04B5.6 | T2 | 6.82 | 0 | hlh1_T2 | end1_T2 | 14.57 | 2.14 | 88.19 | 8.77 |
| WBGene00011004_R04B5.6_R04B5.6 | T3 | 17.93 | 0 | hlh1_T3 | nhr25_T3 | 34.08 | 1.9 | 88.19 | 13.3 |
| WBGene00011004_R04B5.6_R04B5.6 | T4 | 15.36 | 0 | hlh1_T4 | ceh32_T4 | 61.15 | 3.98 | 88.19 | 23.56 |
|  |  |  |  |  |  |  |  |  |  |
| WBGene00011141_R08B4.5_R08B4.5 | T0 | 16.3 | 0 | cnd1_T0 | nhr25_T0 | 33.27 | 2.04 | 22.34 | 15.61 |
| WBGene00011141_R08B4.5_R08B4.5 | T4 | 6.3 | 0.06 | ceh32_T4 | nhr25_T4 | 99.59 | 15.81 | 22.34 | 7.28 |
|  |  |  |  |  |  |  |  |  |  |
| WBGene00011175_R09E10.5_R09E10.5 | T0 | 11.79 | 0.02 | tbx37_T0 | hlh1_T0 | 40.51 | 3.44 | 5298.43 | 10.74 |
| WBGene00011175_R09E10.5_R09E10.5 | T1 | 28.39 | 0.01 | pha4_T1 | cnd1_T1 | 252.41 | 8.89 | 5298.43 | 22.23 |
| WBGene00011175_R09E10.5_R09E10.5 | T2 | 21.16 | 0.08 | pha4_T2 | ceh32_T2 | 563.09 | 26.61 | 5298.43 | 13.74 |
| WBGene00011175_R09E10.5_R09E10.5 | T4 | 15.61 | 0.09 | pha4_T4 | ceh32_T4 | 1844.85 | 118.21 | 5298.43 | 11.61 |
|  |  |  |  |  |  |  |  |  |  |
| WBGene00011422_T04B2.3_T04B2.3 | T0 | 7.3 | 0.07 | nhr25_T0 | cnd1_T0 | 33.12 | 4.54 | 140.77 | 7.94 |
| WBGene00011422_T04B2.3_T04B2.3 | T4 | 3.93 | 0.04 | hlh1_T4 | nhr25_T4 | 97.82 | 24.9 | 140.77 | 6.08 |
|  |  |  |  |  |  |  |  |  |  |
| WBGene00013118_Y51H4A.24_Y51H4A.24 | T0 | 8.54 | 0.02 | nhr25_T0 | end1_T0 | 3.92 | 0.46 | 48.89 | 12.33 |
| WBGene00013118_Y51H4A.24_Y51H4A.24 | T1 | 5.53 | 0.04 | nhr25_T1 | end1_T1 | 4.24 | 0.77 | 48.89 | 9.22 |
| WBGene00013118_Y51H4A.24_Y51H4A.24 | T4 | 17.1 | 0 | end1_T4 | nhr25_T4 | 49.76 | 2.91 | 48.89 | 19.48 |
|  |  |  |  |  |  |  |  |  |  |
| WBGene00013147_Y53C12C.1_eyg-1 | T2 | 15.39 | 0.01 | tbx37_T2 | hlh1_T2 | 155.6 | 10.11 | 224.18 | 10.6 |
| WBGene00013147_Y53C12C.1_eyg-1 | T3 | 44.2 | 0 | tbx37_T3 | hlh1_T3 | 175.34 | 3.97 | 224.18 | 28.47 |
| WBGene00013147_Y53C12C.1_eyg-1 | T4 | 11.01 | 0.05 | pha4_T4 | cnd1_T4 | 96.41 | 8.76 | 224.18 | 10.75 |
|  |  |  |  |  |  |  |  |  |  |
| WBGene00013328_Y57G11C.39_Y57G11C.39 | T0 | 6.45 | 0.03 | tbx37_T0 | hlh1_T0 | 2.41 | 0.37 | 64.54 | 17.03 |
| WBGene00013328_Y57G11C.39_Y57G11C.39 | T3 | 7.97 | 0.09 | pha4_T3 | cnd1_T3 | 91.74 | 11.52 | 64.54 | 8.35 |
| WBGene00013328_Y57G11C.39_Y57G11C.39 | T4 | 15.61 | 0 | pha4_T4 | cnd1_T4 | 297.37 | 19.05 | 64.54 | 13.97 |
|  |  |  |  |  |  |  |  |  |  |
| WBGene00014255_ZK1320.5_ZK1320.5 | T3 | 9.8 | 0.09 | ceh32_T3 | hlh1_T3 | 4.3 | 0.44 | 8.99 | 7.63 |
| WBGene00014255_ZK1320.5_ZK1320.5 | T4 | 14.58 | 0.04 | cnd1_T4 | nhr25_T4 | 11.43 | 0.78 | 8.99 | 9.79 |
|  |  |  |  |  |  |  |  |  |  |
| WBGene00014262_ZK1321.4_ZK1321.4 | T0 | 12.89 | 0.04 | end1_T0 | nhr25_T0 | 10.82 | 0.84 | 26.52 | 11.55 |
| WBGene00014262_ZK1321.4_ZK1321.4 | T4 | 11 | 0.01 | hlh1_T4 | end1_T4 | 16 | 1.46 | 26.52 | 10.78 |
|  |  |  |  |  |  |  |  |  |  |
| WBGene00015980_C18G1.1_C18G1.1 | T0 | 8.05 | 0.04 | end1_T0 | ceh32_T0 | 7.6 | 0.94 | 24.97 | 23.73 |
| WBGene00015980_C18G1.1_C18G1.1 | T3 | 7.08 | 0.03 | hlh1_T3 | end1_T3 | 21.61 | 3.05 | 24.97 | 9.14 |
| WBGene00015980_C18G1.1_C18G1.1 | T4 | 14.93 | 0 | hlh1_T4 | end1_T4 | 34.23 | 2.29 | 24.97 | 20.68 |
|  |  |  |  |  |  |  |  |  |  |
| WBGene00016089_C25B8.5_aexr-1 | T0 | 13.31 | 0.01 | tbx37_T0 | hlh1_T0 | 4.09 | 0.31 | 131.81 | 18.23 |
| WBGene00016089_C25B8.5_aexr-1 | T1 | 28.44 | 0.03 | ceh32_T1 | nhr25_T1 | 22.92 | 0.81 | 131.81 | 17.79 |
| WBGene00016089_C25B8.5_aexr-1 | T2 | 22.45 | 0.01 | ceh32_T2 | end1_T2 | 48.84 | 2.17 | 131.81 | 20.29 |
|  |  |  |  |  |  |  |  |  |  |
| WBGene00016838_C50F2.4_C50F2.4 | T0 | 9.34 | 0.01 | end1_T0 | cnd1_T0 | 453.01 | 48.48 | 280.94 | 8.35 |
| WBGene00016838_C50F2.4_C50F2.4 | T1 | 6.61 | 0.09 | end1_T1 | hlh1_T1 | 381.42 | 57.69 | 280.94 | 4.97 |
| WBGene00016838_C50F2.4_C50F2.4 | T4 | 6.16 | 0 | hlh1_T4 | end1_T4 | 253.56 | 41.15 | 280.94 | 9.15 |
|  |  |  |  |  |  |  |  |  |  |
| WBGene00016904_C53D5.3_C53D5.3 | T0 | 24.41 | 0 | hlh1_T0 | end1_T0 | 69.56 | 2.85 | 13.56 | 23.7 |
| WBGene00016904_C53D5.3_C53D5.3 | T4 | 4.08 | 0.04 | cnd1_T4 | hlh1_T4 | 170.51 | 41.77 | 13.56 | 5.4 |
|  |  |  |  |  |  |  |  |  |  |
| WBGene00017097_E02C12.12_E02C12.12 | T1 | 3.61 | 0.05 | cnd1_T1 | pha4_T1 | 3.1 | 0.86 | 4.85 | 29.75 |
| WBGene00017097_E02C12.12_E02C12.12 | T2 | 3.21 | 0.09 | ceh32_T2 | pha4_T2 | 2.97 | 0.92 | 4.85 | 16.61 |
|  |  |  |  |  |  |  |  |  |  |
| WBGene00017150_EGAP5.1_EGAP5.1 | T0 | 5.19 | 0 | end1_T0 | hlh1_T0 | 26.16 | 5.04 | 6.41 | 58.44 |
| WBGene00017150_EGAP5.1_EGAP5.1 | T4 | 12.26 | 0.07 | ceh32_T4 | end1_T4 | 50.88 | 4.15 | 6.41 | 14.77 |
|  |  |  |  |  |  |  |  |  |  |
| WBGene00017326_F10C1.5_dmd-5 | T0 | 16.39 | 0.09 | pha4_T0 | ceh32_T0 | 274.47 | 16.75 | 364.5 | 13.45 |
| WBGene00017326_F10C1.5_dmd-5 | T1 | 31.34 | 0 | tbx37_T1 | end1_T1 | 204.67 | 6.53 | 364.5 | 31.02 |
|  |  |  |  |  |  |  |  |  |  |
| WBGene00017900_F28E10.1_F28E10.1 | T0 | 5.69 | 0 | hlh1_T0 | end1_T0 | 121.04 | 21.27 | 502.18 | 5.81 |
| WBGene00017900_F28E10.1_F28E10.1 | T1 | 5.22 | 0 | hlh1_T1 | cnd1_T1 | 156.48 | 30 | 502.18 | 8.03 |
| WBGene00017900_F28E10.1_F28E10.1 | T4 | 4.52 | 0 | cnd1_T4 | pha4_T4 | 773.74 | 171.06 | 502.18 | 7.29 |
|  |  |  |  |  |  |  |  |  |  |
| WBGene00018373_F43B10.1_F43B10.1 | T0 | 17.36 | 0 | cnd1_T0 | hlh1_T0 | 13.08 | 0.75 | 77.65 | 22.66 |
| WBGene00018373_F43B10.1_F43B10.1 | T1 | 32.54 | 0 | ceh32_T1 | pha4_T1 | 41.38 | 1.27 | 77.65 | 31.26 |
| WBGene00018373_F43B10.1_F43B10.1 | T2 | 19.39 | 0 | ceh32_T2 | end1_T2 | 67.01 | 3.46 | 77.65 | 16.34 |
| WBGene00018373_F43B10.1_F43B10.1 | T3 | 16.71 | 0.01 | ceh32_T3 | nhr25_T3 | 53.33 | 3.19 | 77.65 | 11.52 |
| WBGene00018373_F43B10.1_F43B10.1 | T4 | 13.92 | 0 | ceh32_T4 | pha4_T4 | 61.68 | 4.43 | 77.65 | 12.57 |
|  |  |  |  |  |  |  |  |  |  |
| WBGene00018542_F47C10.4_nhr-187 | T0 | 5.58 | 0.01 | nhr25_T0 | cnd1_T0 | 7.1 | 1.27 | 13.85 | 9.11 |
| WBGene00018542_F47C10.4_nhr-187 | T1 | 6.38 | 0 | nhr25_T1 | cnd1_T1 | 5.74 | 0.9 | 13.85 | 14.47 |
| WBGene00018542_F47C10.4_nhr-187 | T2 | 4.26 | 0.04 | nhr25_T2 | ceh32_T2 | 5.39 | 1.26 | 13.85 | 8.86 |
| WBGene00018542_F47C10.4_nhr-187 | T4 | 15.18 | 0 | ceh32_T4 | nhr25_T4 | 12.91 | 0.85 | 13.85 | 11.58 |
|  |  |  |  |  |  |  |  |  |  |
| WBGene00018752_F53C3.8_F53C3.8 | T0 | 9.35 | 0.09 | hlh1_T0 | ceh32_T0 | 5.27 | 0.56 | 8.3 | 15.21 |
| WBGene00018752_F53C3.8_F53C3.8 | T4 | 9.65 | 0.07 | nhr25_T4 | ceh32_T4 | 14.5 | 1.5 | 8.3 | 15.01 |
|  |  |  |  |  |  |  |  |  |  |
| WBGene00020011_R11G1.2_R11G1.2 | T0 | 4.91 | 0.09 | cnd1_T0 | hlh1_T0 | 62.2 | 12.68 | 460.22 | 4.77 |
| WBGene00020011_R11G1.2_R11G1.2 | T1 | 6.16 | 0 | ceh32_T1 | pha4_T1 | 133.33 | 21.64 | 460.22 | 9.38 |
| WBGene00020011_R11G1.2_R11G1.2 | T2 | 7.74 | 0 | ceh32_T2 | pha4_T2 | 179.26 | 23.15 | 460.22 | 10.79 |
| WBGene00020011_R11G1.2_R11G1.2 | T3 | 5.02 | 0.04 | ceh32_T3 | pha4_T3 | 149.7 | 29.84 | 460.22 | 6.19 |
|  |  |  |  |  |  |  |  |  |  |
| WBGene00020205_T04B8.3_arrd-1 | T3 | 10.63 | 0.01 | cnd1_T3 | hlh1_T3 | 144.56 | 13.6 | 863.28 | 6.49 |
| WBGene00020205_T04B8.3_arrd-1 | T4 | 6.23 | 0.08 | ceh32_T4 | end1_T4 | 63.36 | 10.17 | 863.28 | 5.38 |
|  |  |  |  |  |  |  |  |  |  |
| WBGene00020219_T05A7.1_T05A7.1 | T0 | 11.93 | 0 | end1_T0 | ceh32_T0 | 107.08 | 8.97 | 64.56 | 12.24 |
| WBGene00020219_T05A7.1_T05A7.1 | T1 | 10.17 | 0 | end1_T1 | hlh1_T1 | 70.41 | 6.93 | 64.56 | 8.03 |
| WBGene00020219_T05A7.1_T05A7.1 | T4 | 3.2 | 0.08 | hlh1_T4 | end1_T4 | 34.47 | 10.77 | 64.56 | 4.76 |
|  |  |  |  |  |  |  |  |  |  |
| WBGene00020581_T19D12.6_T19D12.6 | T0 | 13.06 | 0 | ceh32_T0 | hlh1_T0 | 13.12 | 1 | 427.58 | 15.64 |
| WBGene00020581_T19D12.6_T19D12.6 | T1 | 7.09 | 0.02 | ceh32_T1 | hlh1_T1 | 27.69 | 3.9 | 427.58 | 8.28 |
| WBGene00020581_T19D12.6_T19D12.6 | T3 | 13.24 | 0.07 | cnd1_T3 | hlh1_T3 | 121.27 | 9.16 | 427.58 | 6.65 |
|  |  |  |  |  |  |  |  |  |  |
| WBGene00021050_W05H9.3_W05H9.3 | T0 | 7.3 | 0 | cnd1_T0 | hlh1_T0 | 2974.93 | 407.58 | 3469.87 | 5.27 |
| WBGene00021050_W05H9.3_W05H9.3 | T1 | 11.82 | 0 | ceh32_T1 | pha4_T1 | 5425.2 | 458.94 | 3469.87 | 7.75 |
| WBGene00021050_W05H9.3_W05H9.3 | T2 | 9.47 | 0 | ceh32_T2 | pha4_T2 | 4150.68 | 438.47 | 3469.87 | 5.9 |
| WBGene00021050_W05H9.3_W05H9.3 | T3 | 7.55 | 0.01 | ceh32_T3 | pha4_T3 | 1664.89 | 220.56 | 3469.87 | 5.44 |
|  |  |  |  |  |  |  |  |  |  |
| WBGene00021272_Y23H5B.1_Y23H5B.1 | T0 | 16.25 | 0.04 | ceh32_T0 | end1_T0 | 326.22 | 20.08 | 138.69 | 11.68 |
| WBGene00021272_Y23H5B.1_Y23H5B.1 | T2 | 17.66 | 0 | tbx37_T2 | end1_T2 | 125.4 | 7.1 | 138.69 | 16.25 |
| WBGene00021272_Y23H5B.1_Y23H5B.1 | T3 | 24.95 | 0.01 | cnd1_T3 | hlh1_T3 | 65.49 | 2.63 | 138.69 | 11.98 |
| WBGene00021272_Y23H5B.1_Y23H5B.1 | T4 | 30.94 | 0 | cnd1_T4 | end1_T4 | 100.97 | 3.26 | 138.69 | 19.24 |
|  |  |  |  |  |  |  |  |  |  |
| WBGene00021788_Y51H7C.10_Y51H7C.10 | T1 | 33.88 | 0 | pha4_T1 | ceh32_T1 | 120.44 | 3.56 | 108.97 | 28.15 |
| WBGene00021788_Y51H7C.10_Y51H7C.10 | T2 | 28.25 | 0 | pha4_T2 | hlh1_T2 | 124.11 | 4.39 | 108.97 | 17.88 |
| WBGene00021788_Y51H7C.10_Y51H7C.10 | T3 | 23.82 | 0 | pha4_T3 | ceh32_T3 | 137.08 | 5.75 | 108.97 | 20 |
| WBGene00021788_Y51H7C.10_Y51H7C.10 | T4 | 30.66 | 0 | tbx37_T4 | nhr25_T4 | 101.33 | 3.31 | 108.97 | 20.07 |
|  |  |  |  |  |  |  |  |  |  |
| WBGene00022465_Y110A7A.20_ift-20 | T1 | 21.95 | 0.02 | cnd1_T1 | hlh1_T1 | 71.85 | 3.27 | 73.52 | 16.81 |
| WBGene00022465_Y110A7A.20_ift-20 | T2 | 19.33 | 0.03 | ceh32_T2 | end1_T2 | 400.34 | 20.71 | 73.52 | 15.8 |
| WBGene00022465_Y110A7A.20_ift-20 | T3 | 51.12 | 0 | ceh32_T3 | hlh1_T3 | 468.65 | 9.17 | 73.52 | 23.4 |
| WBGene00022465_Y110A7A.20_ift-20 | T4 | 37.12 | 0.01 | ceh32_T4 | nhr25_T4 | 852.75 | 22.98 | 73.52 | 30.46 |
|  |  |  |  |  |  |  |  |  |  |
| WBGene00044144_M153.4_M153.4 | T0 | 6.8 | 0 | end1_T0 | ceh32_T0 | 8.7 | 1.28 | 63.27 | 70.93 |
| WBGene00044144_M153.4_M153.4 | T1 | 6.57 | 0.01 | end1_T1 | ceh32_T1 | 10.67 | 1.63 | 63.27 | 12.52 |
| WBGene00044144_M153.4_M153.4 | T2 | 3.7 | 0 | end1_T2 | hlh1_T2 | 10.81 | 2.92 | 63.27 | 9.21 |
| WBGene00044144_M153.4_M153.4 | T4 | 9.56 | 0.01 | cnd1_T4 | hlh1_T4 | 73.27 | 7.67 | 63.27 | 7.76 |
|  |  |  |  |  |  |  |  |  |  |
| WBGene00044978_F58D2.3_F58D2.3 | T0 | 4.96 | 0.04 | tbx37_T0 | end1_T0 | 51.38 | 10.35 | 48.91 | 6.77 |
| WBGene00044978_F58D2.3_F58D2.3 | T3 | 5.78 | 0.07 | cnd1_T3 | pha4_T3 | 18.11 | 3.14 | 48.91 | 8.45 |
|  |  |  |  |  |  |  |  |  |  |
| WBGene00077714_R102.11_R102.11 | T0 | 6.3 | 0.06 | end1_T0 | ceh32_T0 | 28.49 | 4.52 | 7.72 | 13.8 |
| WBGene00077714_R102.11_R102.11 | T1 | 2.63 | 0.03 | end1_T1 | ceh32_T1 | 10.12 | 3.85 | 7.72 | 29.36 |
| WBGene00077714_R102.11_R102.11 | T4 | 13.47 | 0.05 | cnd1_T4 | end1_T4 | 39.16 | 2.91 | 7.72 | 11.47 |
|  |  |  |  |  |  |  |  |  |  |
| WBGene00194710_C30B5.9_mks-2 | T3 | 30.14 | 0.02 | ceh32_T3 | hlh1_T3 | 53.79 | 1.78 | 28.61 | 19.14 |
| WBGene00194710_C30B5.9_mks-2 | T4 | 35.26 | 0.03 | cnd1_T4 | end1_T4 | 137.14 | 3.89 | 28.61 | 18.23 |
