# Supplementary material for: The C. elegans embryonic transcriptome with tissue, time, and alternative splicing resolution
Source: Genome Res. 2019 Jun;29(6):1036–45. doi: 10.1101/gr.243394.118 (PMC6581053; doi:10.1101/gr.243394.118)
Supplement: Supplemental Material [file supp_gr.243394.118_Supplemental_Table_S7.docx]

Supplemental_Table_S7: Differential splice junction and exon usage for readily classifiable alternative junctions

|  | **alt 5' exon, no overlap with internal exon** | **%** | **alt 5' exon, overlaps internal exon, does not begin with splice** | **%** | ***alt 5' exon, overlaps internal exon, begins with splice** | **%** | **3' mutually exclusive exon** | **%** | **internal mutually exclusive exon** | **%** | **retained intron** | **%** | **alt 3' or 5' splice site** | **%** | **skipped exon** | **%** |
| --- | --- | --- | --- | --- | --- | --- | --- | --- | --- | --- | --- | --- | --- | --- | --- | --- |
| **no differential usage** | 2188 | 63.7 | 428 | 61.2 | 1504 | 67.5 | 122 | 67.4 | 41 | 33.3 | 184 | 66.2 | 2045 | 69.7 | 320 | 50.8 |
| **only exon differential usage** | 295 | 8.6 | 74 | 10.6 | 337 | 15.1 | 19 | 10.5 | 10 | 8.1 | 24 | 8.6 | 596 | 20.3 | 33 | 5.2 |
| **only intron differential usage** | 178 | 5.2 | 80 | 11.4 | 155 | 7.0 | 11 | 6.1 | 16 | 13.0 | 37 | 13,3 | 108 | 3.7 | 67 | 10.6 |
| **both intron and exon differential usage, but not for the same tissue pair** | 53 | 1.5 | 8 | 1.1 | 35 | 1.6 | 1 | 0.6 | 3 | 2.4 | 2 | 0.7 | 12 | 0.4 | 6 | 1.0 |
| **both intron and exon differential usage, same tissue pair** | 722 | 21.0 | 109 | 15.6 | 197 | 8.8 | 28 | 15.5 | 53 | 43.1 | 31 | 11.2 | 175 | 6.0 | 204 | 32.4 |
| **total exons** | 3436 |  | 699 |  | 2228 |  | 181 |  | 123 |  | 278 |  | 2936 |  | 630 |  |
| **total genes** | 2588 |  | 645 |  | 1781 |  | 85 |  | 48 |  | 275 |  | 1151 |  | 442 |  |

*some acceptor sites are used in both cis and trans splicing
