# Supplementary material for: The C. elegans embryonic transcriptome with tissue, time, and alternative splicing resolution
Source: Genome Res. 2019 Jun;29(6):1036–45. doi: 10.1101/gr.243394.118 (PMC6581053; doi:10.1101/gr.243394.118)
Supplement: Supplemental Material [file supp_gr.243394.118_Supplemental_Table_S16.doc]

Supplemental_Table_S16: RNA editing event descriptions

| **chr_pos_ref_alt** | **gene name** | **locus name** | **codon base** | **codon** | **amino acid** | **Grantham** | **Gerp** | **Phylop** | **Phastcons** |
| --- | --- | --- | --- | --- | --- | --- | --- | --- | --- |
| **I_3109473_G_A** | C45E1.1 | nhr-64 | 1 | GGA->GAA | G->E | 98 | 1.17 | 0.156 | 0.133 |
| **I_5117089_C_A** | Y110A7A.16 | elpc-1 | 0 | GTC->TTC | V->F | 50 | 2.47 | 1.626 | 0.994 |
| **II_10351402_G_A** | C07E3.9 | C07E3.9 | 2 | TGG->TGA | W->* | -1 | 2.47 | 1.626 | 0.994 |
| **II_3322004_A_G** | Y46D2A.2 | Y46D2A.2 | 2 | TTT->TTC | F->F | 0 | 2.47 | 1.626 | 0.994 |
| **II_961361_A_G** | C32B5.13 | C32B5.13 | 2 | TTT->TTC | F->F | 0 | 2.47 | 1.626 | 0.994 |
| **II_961362_A_G** | C32B5.13 | C32B5.13 | 1 | TTT->TCT | F->S | 155 | 2.47 | 1.626 | 0.994 |
| **II_961374_A_G** | C32B5.13 | C32B5.13 | 1 | GTA->GCA | V->A | 64 | 2.47 | 1.626 | 0.994 |
| **II_963980_A_G** | C32B5.7 | C32B5.7 | 0 | ATT->GTT | I->V | 29 | 2.47 | 1.626 | 0.994 |
| **III_1346526_G_A** | Y82E9BL.5 | Y82E9BL.5 | 1 | CCA->CTA | P->L | 98 | 2.47 | 1.626 | 0.994 |
| **III_13604566_T_G** | T25C8.2 | act-5 | 2 | CGA->CGC | R->R | 0 | 2.47 | 1.626 | 0.994 |
| **III_13604620_T_C** | T25C8.2 | act-5 | 2 | CAA->CAG | Q->Q | 0 | 2.47 | 1.626 | 0.994 |
| **III_13604638_G_C** | T25C8.2 | act-5 | 2 | TCC->TCG | S->S | 0 | 2.47 | 1.626 | 0.994 |
| **III_13604644_A_C** | T25C8.2 | act-5 | 2 | CTT->CTG | L->L | 0 | 2.47 | 1.626 | 0.994 |
| **III_13604647_A_G** | T25C8.2 | act-5 | 2 | ATT->ATC | I->I | 0 | 2.47 | 1.626 | 0.994 |
| **III_13605137_T_A** | T25C8.2 | act-5 | 0 | ACT->TCT | T->S | 58 | 2.47 | 1.626 | 0.994 |
| **III_13605153_G_C** | T25C8.2 | act-5 | 2 | CTC->CTG | L->L | 0 | 2.47 | 1.626 | 0.994 |
| **III_13605167_T_G** | T25C8.2 | act-5 | 0 | ATG->CTG | M->L | 15 | 2.47 | 1.626 | 0.994 |
| **III_13605841_C_T** | T25C8.2 | act-5 | 0 | GTC->ATC | V->I | 29 | 2.47 | 1.626 | 0.994 |
| **III_13605845_T_A** | T25C8.2 | act-5 | 2 | GGA->GGT | G->G | 0 | 2.47 | 1.626 | 0.994 |
| **III_13605857_T_G** | T25C8.2 | act-5 | 2 | CCA->CCC | P->P | 0 | 2.47 | 1.626 | 0.994 |
| **IV_11326977_C_T** | B0035.10 | his-45 | 2 | CGC->CGT | R->R | 0 | 2.47 | 1.626 | 0.994 |
| **V_11072530_G_C** | T04C12.4 | act-3 | 2 | CTC->CTG | L->L | 0 | 2.47 | 1.626 | 0.994 |
| **V_11072536_G_A** | T04C12.4 | act-3 | 2 | GTC->GTT | V->V | 0 | 2.47 | 1.626 | 0.994 |
| **V_11072539_G_A** | T04C12.4 | act-3 | 2 | TAC->TAT | Y->Y | 0 | 2.47 | 1.626 | 0.994 |
| **V_11072545_G_C** | T04C12.4 | act-3 | 2 | CTC->CTG | L->L | 0 | 2.47 | 1.626 | 0.994 |
| **V_11072547_G_A** | T04C12.4 | act-3 | 0 | CTC->TTC | L->F | 22 | 2.47 | 1.626 | 0.994 |
| **V_11072715_T_A** | T04C12.4 | act-3 | 0 | ACC->TCC | T->S | 58 | 2.47 | 1.626 | 0.994 |
| **V_11072725_T_A** | T04C12.4 | act-3 | 2 | GGA->GGT | G->G | 0 | 2.47 | 1.626 | 0.994 |
| **V_11072728_A_G** | T04C12.4 | act-3 | 2 | TCT->TCC | S->S | 0 | 2.47 | 1.626 | 0.994 |
| **V_11072737_G_A** | T04C12.4 | act-3 | 2 | GTC->GTT | V->V | 0 | 2.47 | 1.626 | 0.994 |
| **V_11072742_C_T** | T04C12.4 | act-3 | 0 | GTC->ATC | V->I | 29 | 2.47 | 1.626 | 0.994 |
| **V_11072743_T_A** | T04C12.4 | act-3 | 2 | GGA->GGT | G->G | 0 | 2.47 | 1.626 | 0.994 |
| **V_11072761_A_G** | T04C12.4 | act-3 | 2 | GCT->GCC | A->A | 0 | 2.47 | 1.626 | 0.994 |
| **V_11081561_A_T** | T04C12.6 | act-1 | 2 | GGA->GGT | G->G | 0 | 2.47 | 1.626 | 0.994 |
| **V_11081702_T_G** | T04C12.6 | act-1 | 2 | CTT->CTG | L->L | 0 | 2.47 | 1.626 | 0.994 |
| **V_11081771_C_G** | T04C12.6 | act-1 | 2 | CTC->CTG | L->L | 0 | 2.47 | 1.626 | 0.994 |
| **V_7814688_G_T** | ZK742.6 | ZK742.6 | 1 | ACA->AAA | T->K | 78 | 4.67 | 2.847 | 0.999 |
| **V_8536686_T_G** | F45F2.2 | his-39 | 2 | AAT->AAG | N->K | 94 | 2.16 | 0.748 | 0.997 |
| **X_16538685_A_G** | T27B1.2 | ztf-19 | 1 | CAG->CGG | Q->R | 43 | 2.16 | 0.748 | 0.997 |
| **X_4192107_T_C** | F20B6.2 | vha-12 | 2 | CCT->CCC | P->P | 0 | 3.76 | 1.4 | 0.999 |
| **X_4192110_A_T** | F20B6.2 | vha-12 | 2 | GGA->GGT | G->G | 0 | 4.67 | 1.908 | 1 |
| **X_4192122_T_C** | F20B6.2 | vha-12 | 2 | ACT->ACC | T->T | 0 | 4.67 | 2.145 | 1 |
| **X_4192242_T_C** | F20B6.2 | vha-12 | 2 | ACT->ACC | T->T | 0 | 3.42 | 0.917 | 0.998 |
| **X_4192260_A_C** | F20B6.2 | vha-12 | 2 | GGA->GGC | G->G | 0 | 4.67 | 1.908 | 1 |
| **X_6475973_C_A** | C15B12.8 | C15B12.8 | 0 | CAT->AAT | H->N | 68 | -9.35 | -0.799 | 0.251 |
| **X_9097455_G_A** | H08J11.2 | H08J11.2 | 2 | AAA->AAA | K->K | 0 | 4.47 | 2.712 | 1 |
| **X_9097456_T_A** | H08J11.2 | H08J11.2 | 0 | AGA->AGA | R->R | 0 | 4.47 | 2.036 | 1 |
| **X_9097457_G_A** | H08J11.2 | H08J11.2 | 1 | AGA->AAA | R->K | 26 | 3.24 | 0.701 | 0.996 |
