# Supplementary material for: The C. elegans embryonic transcriptome with tissue, time, and alternative splicing resolution
Source: Genome Res. 2019 Jun;29(6):1036–45. doi: 10.1101/gr.243394.118 (PMC6581053; doi:10.1101/gr.243394.118)
Supplement: Supplemental Material [file supp_gr.243394.118_Supplemental_Table_S18.doc]

Supplemental_Table_S17: List of transcription factors showing tissue-specificity based on DESeq2 analysis

| ***ceh-32* TFs** | ***cnd-1* TFs** | ***end-1* TFs** | ***hlh-1*TFs** | ***nhr-25* TFs** | ***pha-4* TFs** | ***tbx-37* TFs** |
| --- | --- | --- | --- | --- | --- | --- |
| *dac-1* | *unc-30* | *odd-1* | *hlh-1* | *nhr-148* | *ceh-53* | *ceh-36* |
| *ceh-5* | *madf-5* | *nhr-63* | *rnt-1* | *nhr-76* | *ceh-2* | *ref-2* |
| *fkh-10* | *ceh-57* | *nhr-28* | *lin-39* | *nhr-150* | *dmd-4* | *che-1* |
| *ceh-43* | *ceh-31* | *nhr-153* | *egl-5* | *nhr-152* | *ceh-22* | *ceh-27* |
| *ttx-3* | *cnd-1* | *elt-7* | *npax-4* | *nhr-94* | *pax-1* | *ceh-32* |
| *tab-1* | *ets-5* | *ceh-79* | *ceh-33* | *ceh-16* | *pha-2* | *tbx-7* |
| *nhr-188* | *bar-1* | *elt-2* | *unc-120* | *nhr-120* | *ztf-16* |  |
| *hmg-6* | *hlh-17* | *nhr-162* | *unc-98* | *nhr-43* | *hlh-6* |  |
| *unc-39* | *fkh-8* | *zip-5* | *sem-4* | *nhr-168* | *pes-1* |  |
| *ceh-28* | *ces-1* | *elt-4* | *ceh-49* | *nhr-169* |  |  |
| *hlh-34* | *hlh-19* | *lpd-2* | *let-381* | *nhr-171* |  |  |
| *dhhc-11* | *ceh-6* | *ets-9* | *ccch-1* | *nhr-116* |  |  |
| *ceh-10* | *aptf-1* | *nhr-121* | *ztf-26* | *nhr-175* |  |  |
| *ngn-1* | *egl-46* | *nhr-36* | *K02D7.2* | *nhr-25* |  |  |
| *ceh-8* | *pros-1* | *nhr-176* | *K05F1.5* | *nhr-178* |  |  |
| *grl-25* | *ceh-90* | *ets-4* | *M03D4.4* | *lin-26* |  |  |
| *hlh-10* | *ast-1* | *moe-3* | *somi-1* | *F21A9.2* |  |  |
|  | *hlh-32* | *nhr-8* | *vab-7* | *nhr-141* |  |  |
|  | *unc-3* | *nhr-108* | *vab-15* | *F26A10.2* |  |  |
|  | *Y17G7B.22* | *pqm-1* | *camt-1* | *nhr-4* |  |  |
|  | *ceh-44* | *nhr-37* | *pat-9* | *nhr-20* |  |  |
|  | *ceh-9* | *mgl-2* | *W04B5.2* | *ctbp-1* |  |  |
|  | *lin-11* | *lsy-27* | *hsf-1* | *elt-3* |  |  |
|  |  | *klf-3* | *mab-3* | *R05D3.3* |  |  |
|  |  | *F55B11.4* | *ceh-18* | *nhr-270* |  |  |
|  |  | *nhr-193* | *ZK337.2* | *tbx-8* |  |  |
|  |  | *end-1* | *syd-9* | *zim-3* |  |  |
|  |  | *nhr-80* | *sdz-38* | *zim-1* |  |  |
|  |  | *nhr-68* |  | *nhr-66* |  |  |
|  |  | *nhr-101* |  | *nhr-218* |  |  |
|  |  | *nhr-53* |  | *nhr-127* |  |  |
|  |  | *nhr-205* |  | *elt-1* |  |  |
|  |  | *nhr-209* |  | *nhr-145* |  |  |
|  |  | *nhr-104* |  | *Y41D4B.26* |  |  |
|  |  | *nhr-132* |  | *nhr-114* |  |  |
|  |  | *nhr-210* |  | *tra-1* |  |  |
|  |  | *nhr-131* |  | *ehn-3* |  |  |
|  |  | *nhr-211* |  | *nhr-256* |  |  |
|  |  | *nhr-212* |  |  |  |  |
|  |  | *gla-3* |  |  |  |  |
|  |  | *nhr-217* |  |  |  |  |
|  |  | *nhr-273* |  |  |  |  |
|  |  | *nhr-16* |  |  |  |  |
|  |  | *T18D3.7* |  |  |  |  |
|  |  | *nhr-220* |  |  |  |  |
|  |  | *sptf-2* |  |  |  |  |
|  |  | *gei-3* |  |  |  |  |
|  |  | *nhr-79* |  |  |  |  |
|  |  | *nhr-115* |  |  |  |  |
|  |  | *zip-10* |  |  |  |  |
|  |  | *nhr-135* |  |  |  |  |
|  |  | *zip-3* |  |  |  |  |
|  |  | *nhr-232* |  |  |  |  |
|  |  | *nhr-238* |  |  |  |  |
|  |  | *zip-12* |  |  |  |  |
|  |  | *nhr-246* |  |  |  |  |
|  |  | *dve-1* |  |  |  |  |
