# Supplementary material for: The C. elegans embryonic transcriptome with tissue, time, and alternative splicing resolution
Source: Genome Res. 2019 Jun;29(6):1036–45. doi: 10.1101/gr.243394.118 (PMC6581053; doi:10.1101/gr.243394.118)
Supplement: Supplemental Material [file supp_gr.243394.118_Supplemental_Table_S19.doc]

Supplemental_Table_S18: Transcription factors showing tissue-specificity by fuzzy *k*-means clustering

| **Neuronal TFs** | **Intestinal TFs** | **Muscle TFs** | **Hypodermis TFs** | **Pharyngeal TFs** |
| --- | --- | --- | --- | --- |
| *lim-7* | *dve-1* | *nurf-1* | *ceh-16* | *hlh-6* |
| *alr-1* | *B0310.2* | *camt-1* | *ahr-1* | *C08G9.2* |
| *ceh-88* | *dmd-7* | *ccch-1* | *blmp-1* | *ceh-19* |
| *daf-3* | *elt-2* | *ceh-18* | *ehn-3* | *ceh-2* |
| *unc-130* | *elt-4* | *ceh-33* | *elt-1* | *ceh-22* |
| *lir-3* | *elt-7* | *ces-2* | *elt-3* | *ceh-34* |
| *K12H6.12* | *end-1* | *egl-5* | *F13H6.1* | *ceh-53* |
| *Y37F4.6* | *ets-8* | *hlh-1* | *F21A9.2* | *ceh-60* |
| *nhr-47* | *ets-9* | *hsf-1* | *ham-2* | *cfi-1* |
| *nhr-100* | *F55B11.4* | *K02D7.2* | *lin-1* | *che-1* |
| *nhr-190* | *hnd-1* | *K05F1.5* | *lin-22* | *cky-1* |
| *nhr-95* | *klf-3* | *let-381* | *lin-26* | *dmd-4* |
| *hlh-34* | *mgl-2* | *lin-39* | *nhr-114* | *ets-4* |
| *ceh-5* | *nhr-90* | *M03D4.4* | *nhr-116* | *eyg-1* |
| *ceh-8* | *nhr-92* | *mab-5* | *nhr-119* | *gmeb-2* |
| *tag-68* | *nhr-101* | *nhr-19* | *nhr-120* | *jun-1* |
| *pag-3* | *nhr-102* | *nhr-87* | *nhr-127* | *klf-2* |
| *elt-6* | *nhr-108* | *pat-9* | *nhr-141* | *nfya-2* |
| *nhr-230* | *nhr-109* | *rnt-1* | *nhr-145* | *nhr-117* |
| *aptf-1* | *nhr-115* | *sdz-38* | *nhr-150* | *nhr-123* |
| *hlh-17* | *nhr-12* | *somi-1* | *nhr-152* | *nhr-129* |
| *hlh-13* | *nhr-121* | *syd-9* | *nhr-172* | *nhr-130* |
| *hlh-32* | *nhr-131* | *tlp-1* | *nhr-178* | *nhr-136* |
| *lfi-1* | *nhr-153* | *unc-120* | *nhr-202* | *nhr-143* |
| *ceh-57* | *nhr-16* | *unc-62* | *nhr-218* | *nhr-15* |
| *ceh-54* | *nhr-162* | *unc-98* | *nhr-221* | *nhr-156* |
| *ceh-24* | *nhr-170* | *vab-15* | *nhr-23* | *nhr-173* |
| *ceh-9* | *nhr-176* | *vab-7* | *nhr-25* | *nhr-182* |
| *unc-4* | *nhr-177* | *W04B5.2* | *nhr-270* | *nhr-183* |
| *unc-30* | *nhr-180* | *ZK337.2* | *nhr-31* | *nhr-206* |
| *sox-4* | *nhr-193* |  | *nhr-43* | *nhr-21* |
| *mbr-1* | *nhr-205* |  | *nhr-76* | *nhr-213* |
| *npax-2* | *nhr-208* |  | *nhr-85* | *nhr-225* |
| *mab-9* | *nhr-209* |  | *nhr-94* | *nhr-237* |
| *ets-5* | *nhr-226* |  | *nhr-97* | *nhr-44* |
| *C06E2.1* | *nhr-232* |  | *pax-3* | *nhr-56* |
| *Y17G7B.22* | *nhr-246* |  | *tbx-8* | *nhr-58* |
| *fezf-1* | *nhr-273* |  | *tbx-9* | *nhr-59* |
| *nhr-258* | *nhr-28* |  | *tra-1* | *nhr-62* |
| *nhr-26* | *nhr-53* |  | *Y41D4B.26* | *pax-1* |
| *cnd-1* | *nhr-57* |  | *ztf-29* | *peb-1* |
| *hlh-19* | *nhr-68* |  | *ztf-30* | *pha-2* |
| *hlh-3* | *nhr-69* |  |  | *pha-4* |
| *ceh-90* | *nhr-79* |  |  | *sox-3* |
| *ceh-48* | *nhr-8* |  |  | *sup-37* |
| *ceh-44* | *nhr-80* |  |  | *tbx-7* |
| *lim-6* | *odd-1* |  |  | *ztf-16* |
| *lin-11* | *odd-2* |  |  | *ztf-27* |
| *lim-4* | *pqm-1* |  |  |  |
| *ceh-31* | *sptf-2* |  |  |  |
| *cog-1* | *T18D3.7* |  |  |  |
| *unc-86* | *zip-10* |  |  |  |
| *ceh-6* | *zip-12* |  |  |  |
| *unc-42* | *zip-3* |  |  |  |
| *pros-1* | *zip-5* |  |  |  |
| *egl-13* |  |  |  |  |
| *unc-3* |  |  |  |  |
| *madf-5* |  |  |  |  |
| *bar-1* |  |  |  |  |
| *ast-1* |  |  |  |  |
| *fkh-8* |  |  |  |  |
| *daf-19* |  |  |  |  |
| *egl-46* |  |  |  |  |
| *ces-1* |  |  |  |  |
| *egl-43* |  |  |  |  |
| *zag-1* |  |  |  |  |
| *C34D10.2* |  |  |  |  |
| *atf-8* |  |  |  |  |
| *nhr-157* |  |  |  |  |
| *ctbp-1* |  |  |  |  |
| *mbl-1* |  |  |  |  |
| *dhhc-11* |  |  |  |  |
| *hlh-4* |  |  |  |  |
| *hlh-10* |  |  |  |  |
| *grh-1* |  |  |  |  |
| *ttx-3* |  |  |  |  |
| *ceh-17* |  |  |  |  |
| *dac-1* |  |  |  |  |
| *fkh-10* |  |  |  |  |
| *Y22D7AL.16* |  |  |  |  |
| *nhr-124* |  |  |  |  |
| *fax-1* |  |  |  |  |
| *nhr-51* |  |  |  |  |
| *nhr-52* |  |  |  |  |
