# Supplementary material for: The C. elegans embryonic transcriptome with tissue, time, and alternative splicing resolution
Source: Genome Res. 2019 Jun;29(6):1036–45. doi: 10.1101/gr.243394.118 (PMC6581053; doi:10.1101/gr.243394.118)
Supplement: Supplemental Material [file supp_gr.243394.118_Supplemental_Material.docx]

**
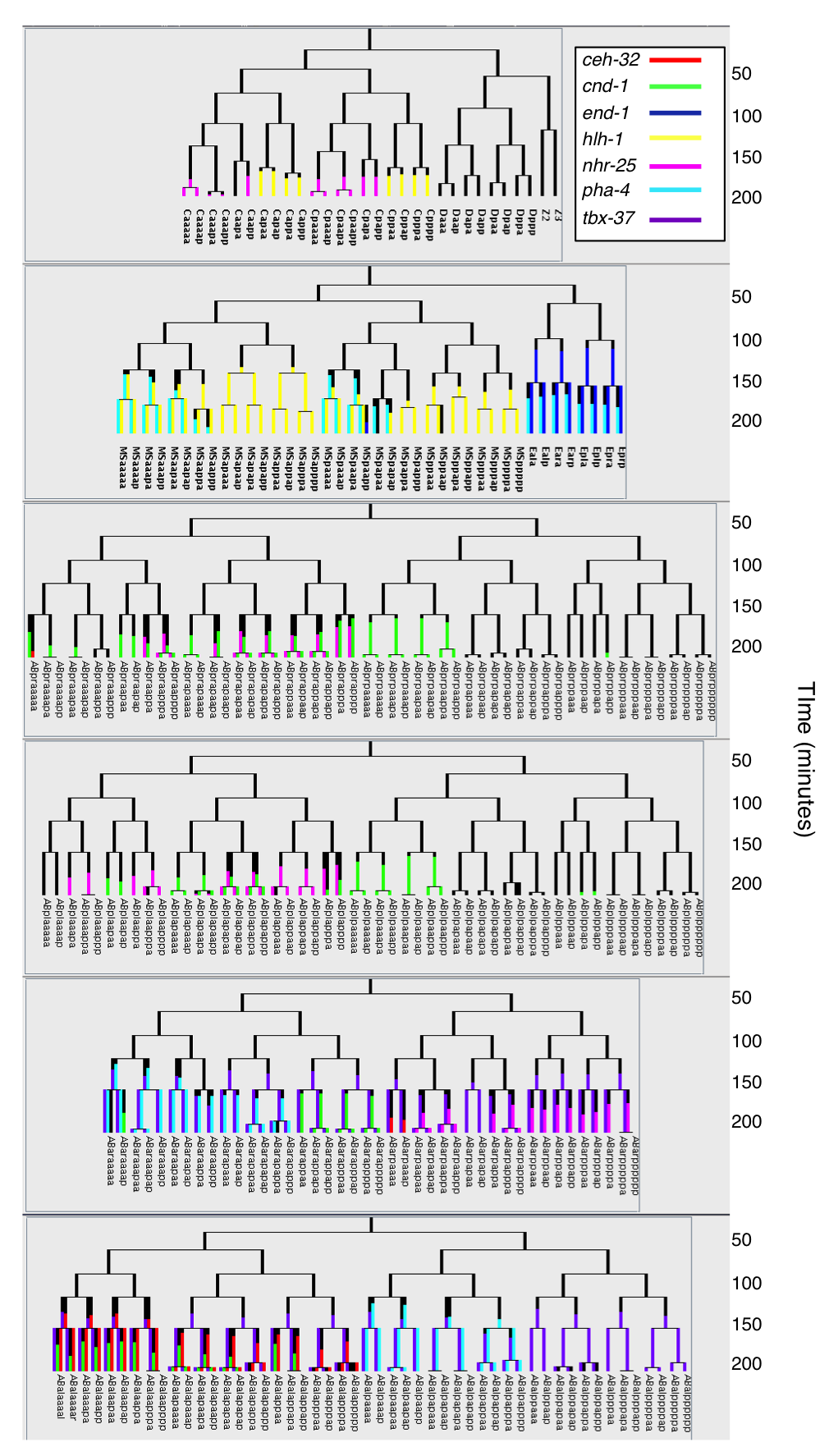
Supplemental_Fig_S1.** Cell lineage trees for each marker strain used in this study.

Each strain used for this study harbored a fluorescently tagged transcription factor marker that allowed us to measure the expression levels, and cellular expression location of the marker in question. Some strains have overlapping populations of cells expressing the markers used such as *tbx-37* which marks the entire ABa lineage and thus overlaps with cells from the *ceh-32*, *cnd-1*, *nhr-25*, and *pha-4* strains. Only the intestinal lineage of E cells expressing *end-1* were obtained in our *end-1* time series. While the *pha-4* strain used does express in the E lineage, our *end-1* marker was used to selectively isolate those cells separately from the pharynx *pha-4* labeled cells.


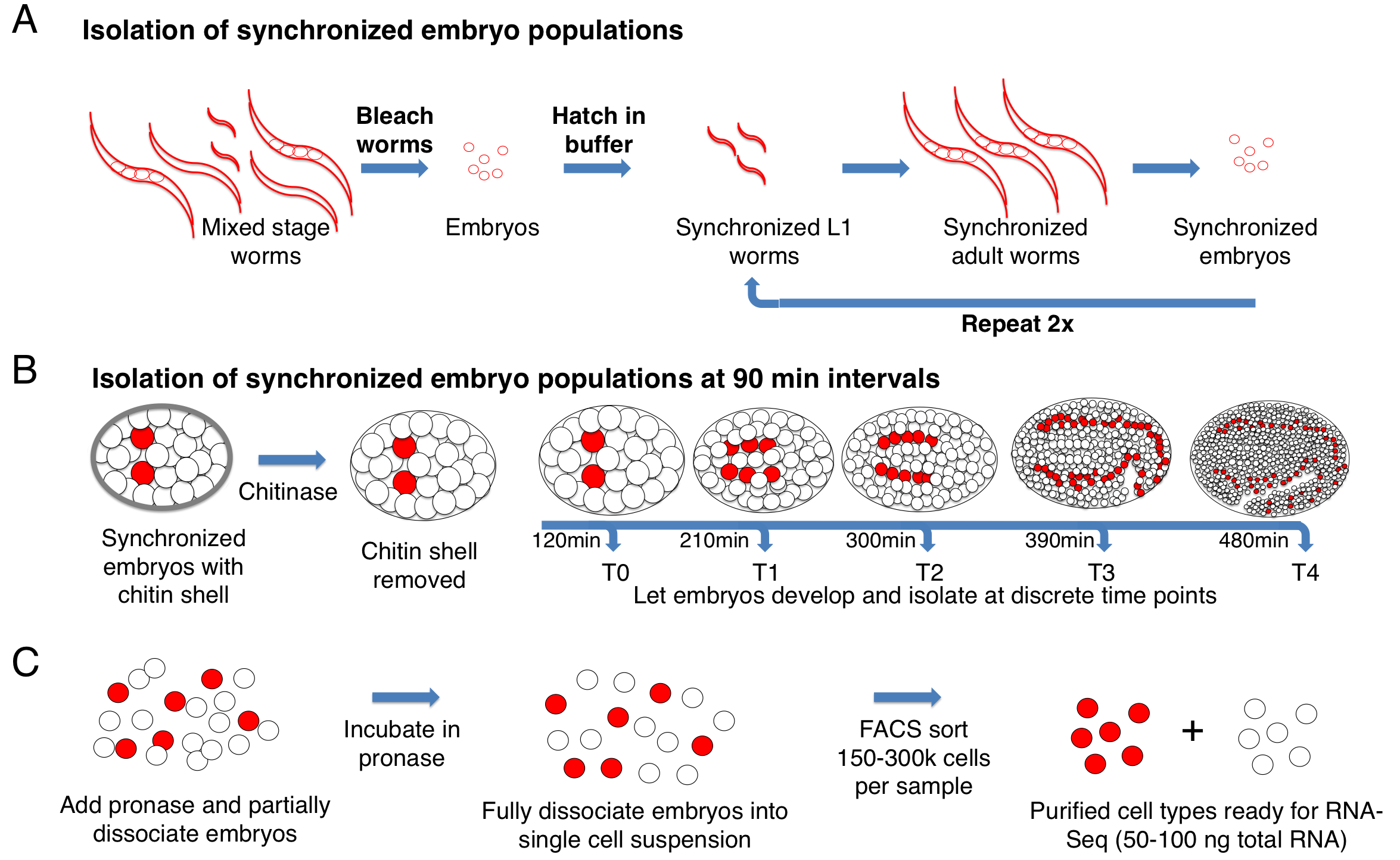


**Supplemental_Fig_S2.** Methodology for isolation of discrete cell types for RNA-seq.

Mixed stage worms were grown to maturity in multiple rounds of synchronization, and treated using standard alkaline bleach treatment to isolate a synchronized population of embryos (A). After embryos were treated with chitinase to release the embryos from their chitin shell, a portion of the growing embryo population was taken at each of five time points 90 minutes apart (B). After treatment with pronase and mechanical disruption (C), single cell suspensions were sorted using FACS to isolate pure fluorescent and non-fluorescent populations of cells.


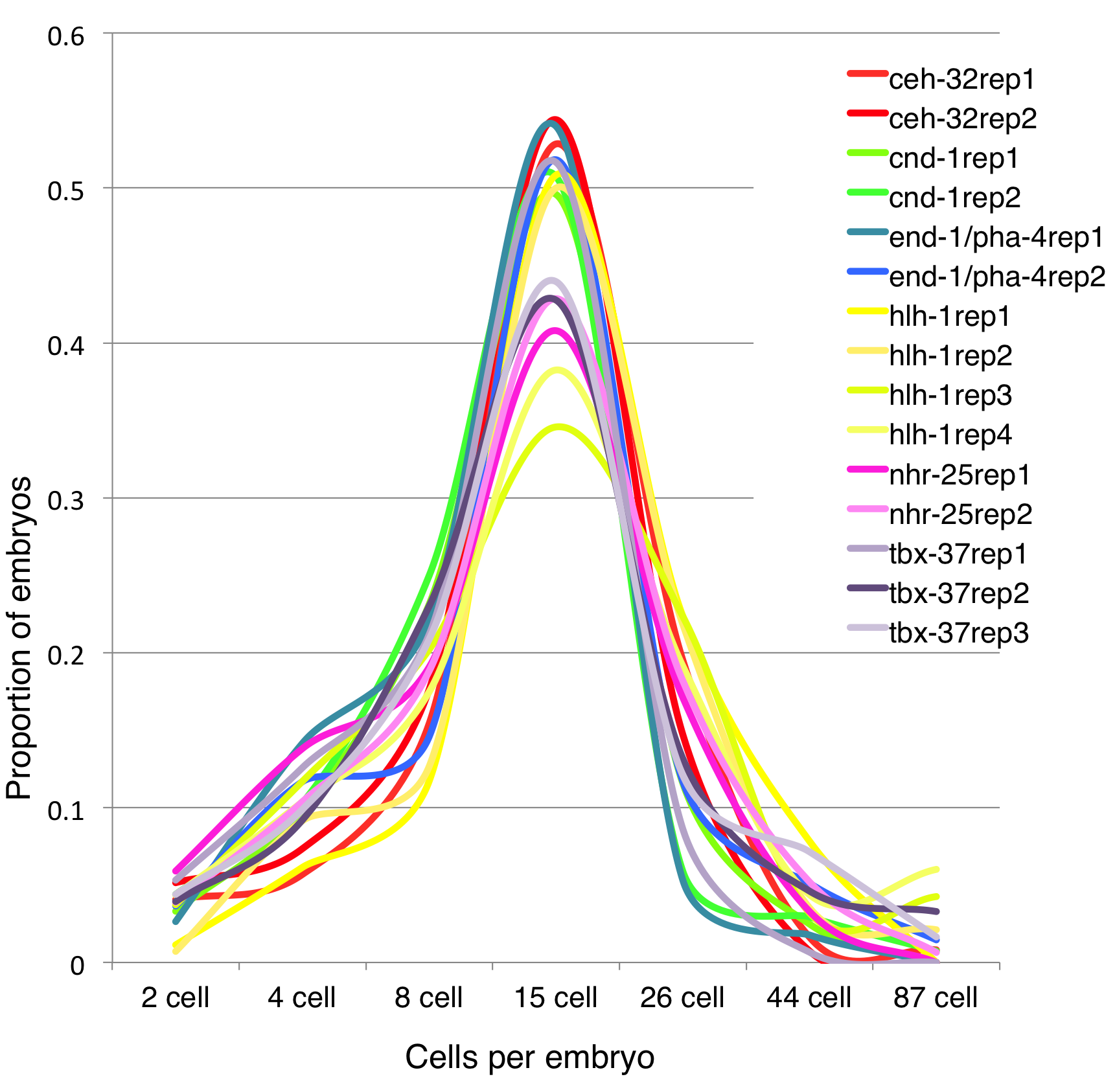


**Supplemental_Fig_S3.** Embryo synchronization leads to a tight window in the age of embryos.

For each time series, embryos were fixed immediately post alkaline bleach treatment to isolate a representative starting population of embryos. Over 100 embryos for each time series were examined by DIC microscopy to count the number of cells per embryo. In each embryo population isolated, the majority of embryos were at the 15-cell stage at the beginning of each experiment demonstrating a highly synchronized population, with only minor variations in younger and older embryos in each series. Time scale ranges from 18 minutes (2 cell) to 125 minutes (87 cell), with most embryos ranging in age by ~60 min (between 8 and 26 cells).


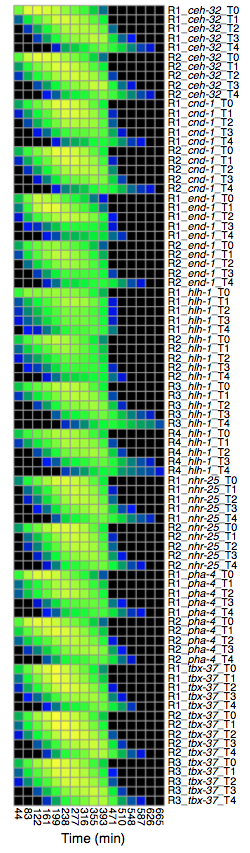


**Supplemental_Fig_S4**. Developmental timing of isolated cell populations.

When gene expression of rapidly changing genes is compared using Spearman’s correlation between isolated cell-type populations and previously published whole embryo populations (Boeck et al., 2016), developmental timing is mirrored. The development follows a linear path over time, and shows the highest correlations at time points beginning ~160 min for the first time points, and ~393 min for the last time points. Early time points correlate better than later time points with whole embryos, as the cells have become more differentiated and are less like whole embryos in terms of gene expression.

**Supplemental_Fig_S5**. Spearman’s correlation values for all samples

Spearman’s correlation values were calculated for each pairwise combination of samples across all sequenced libraries including all replicates. All genes with TPM values equal to or above 15 were used for the analysis. Samples correlate highly with similar time-points within each tissue, and correlate less with those in more divergent time-points and tissues. Scale ranges from 0.65 (black) to 1 (yellow).

**Supplemental_Fig_S6**. PCA plots for replicate samples within each tissue time series

PCA plots were produced for each tissue that was analyzed in this study. Within each tissue, each time point has a different color: T0 (red), T1 (mustard green), T2 (green), T3 (blue), T4 (purple). In general, replicates at each time point group closely, and this persists across time-points. PC1 and PC2 variance scales vary between plots and are marked as indicated.


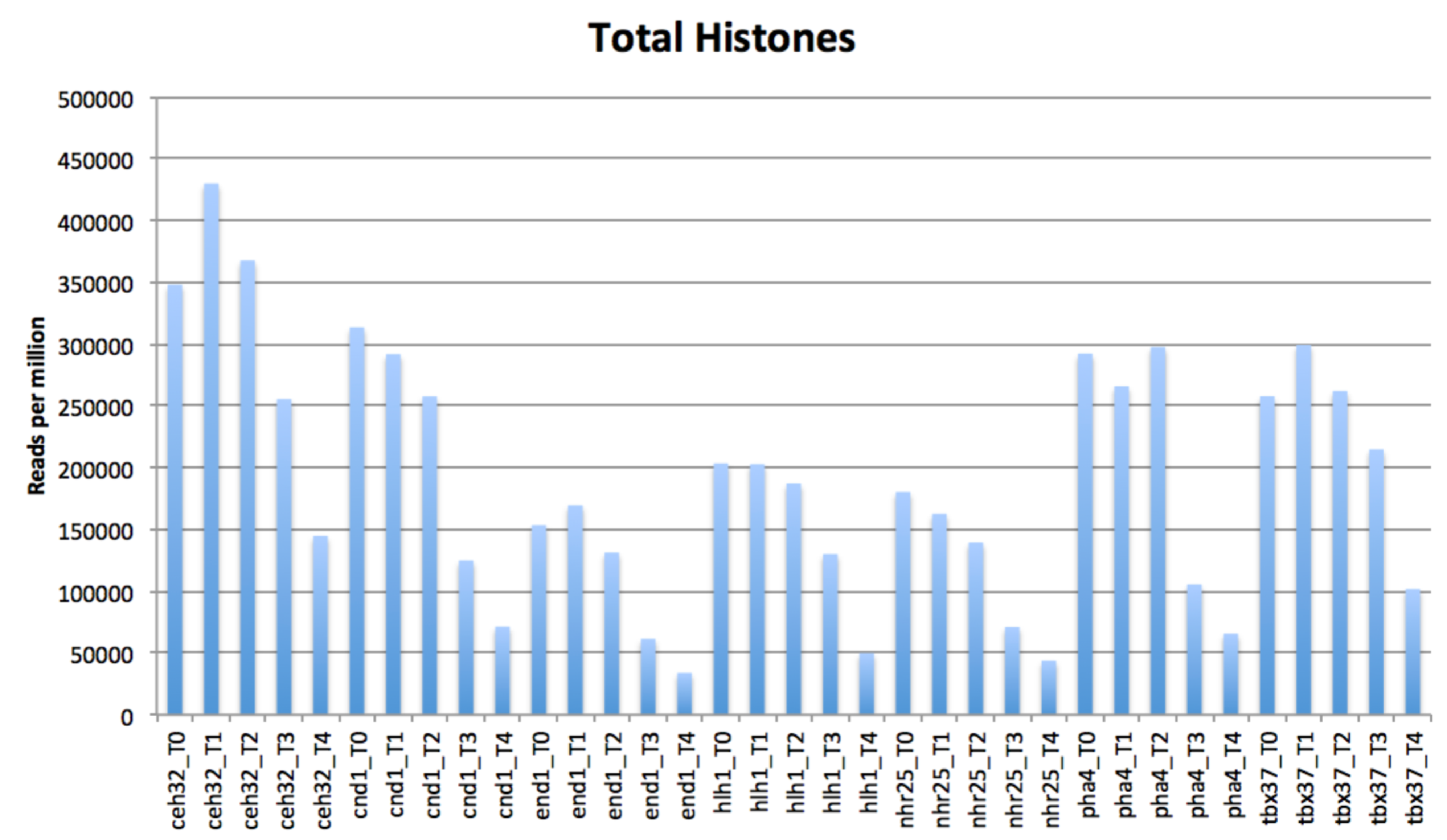


**Supplemental_Fig_S7.** Reads per million accounted for by histones. Reads derived from non-polyadenylated histone messages dominated the early samples.


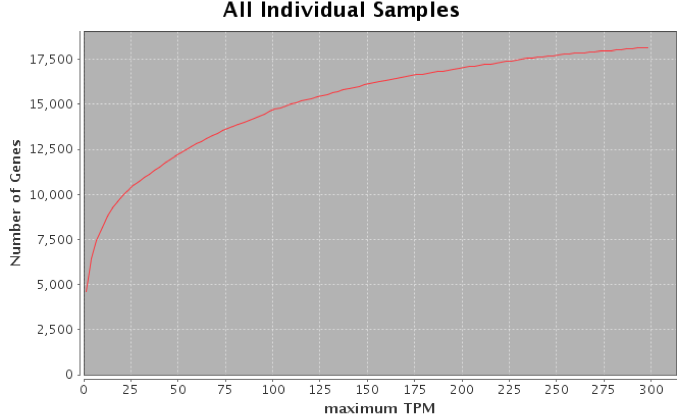


**Supplemental_Fig_S8.** Gene expression above TPM 15 is robust.

As gene expression rises to TPM 15, the number of genes that meets this threshold continues to rise, but begins to level off. Genes below this level tend to have more variance between samples, making downstream analysis less reliable.


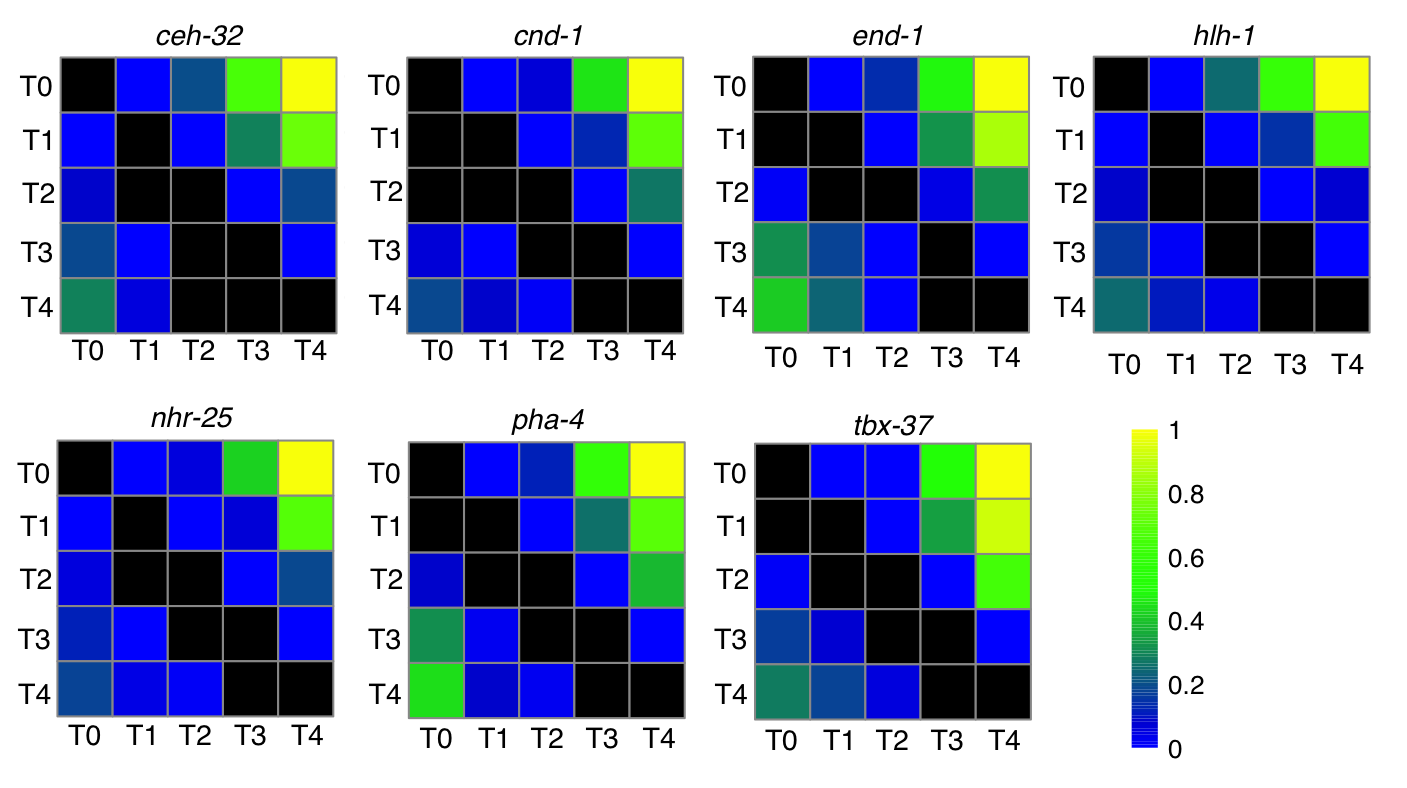


**Supplemental_Fig_S9.** Differentially expressed genes within tissues over time.

Total numbers of differentially expressed genes were scaled to a max of 1 to compare the numbers of differentially expressed genes within a given tissue type over time. Looking within a column, the value is the scaled number of differentially expressed genes higher in the sample on the x-axis, compared to the sample on the y-axis. For example, the top right square in the *ceh-32* heatmap displays the scaled number of differentially expressed genes in T4 when compared with T0. Conversely, within a row, value is the scaled number of differentially expressed genes lower in the sample on the x-axis, compared to the sample on the y-axis.


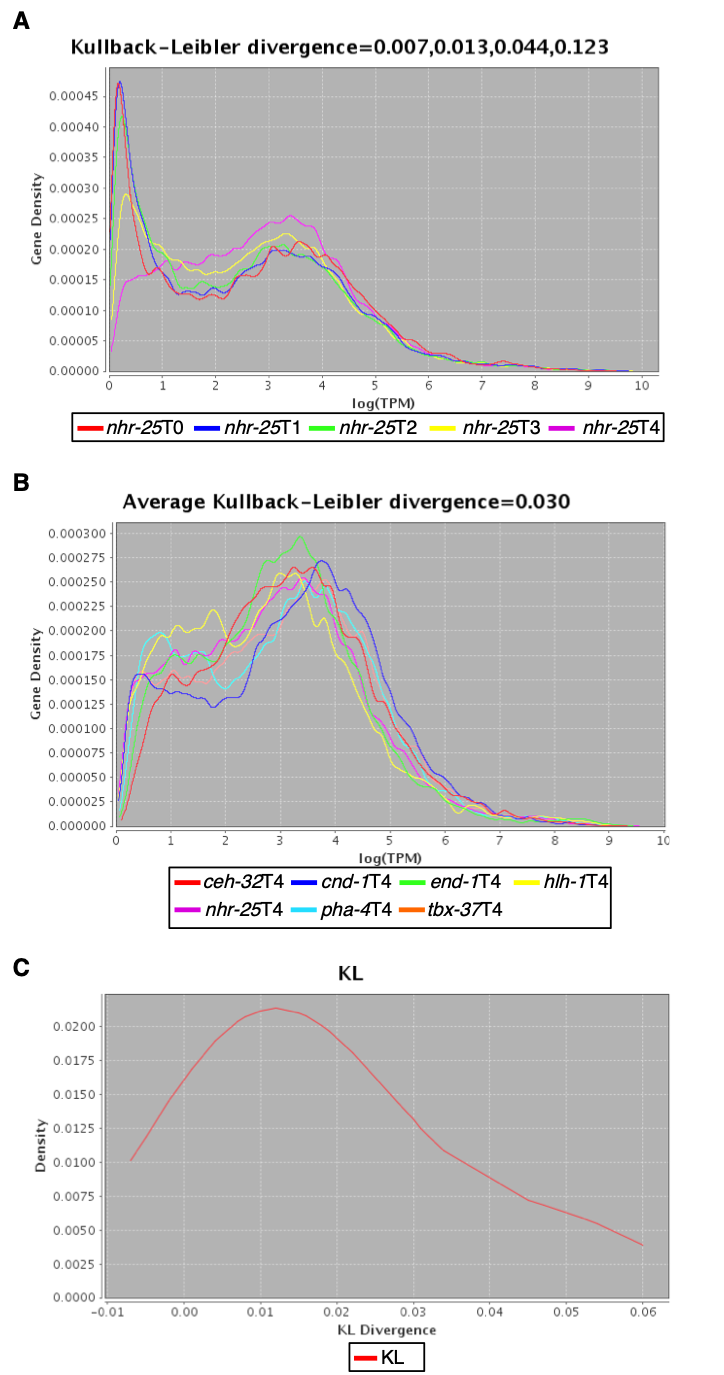


**Supplemental Figure S10.** Gene density calculated using a Kernel Density Estimator.

Divergence of these density functions was estimated using Kullback-Leibler divergence (Supplementary Methods). Hypodermal *nhr-25* samples are shown as a representative of the times within tissues (A) and timepoint 4 for all tissues is shown as a representative example of differences between tissues (B). Replicates for controls for Kullback-Leibler divergence (*hlh-1* series' not included) are displayed (C) to get a baseline expectation of the range of the Kullback-Leibler divergence values, and divergence was calculated for all the possible pairings of the replicate samples (not averaged) and the distribution of those divergences was plotted.


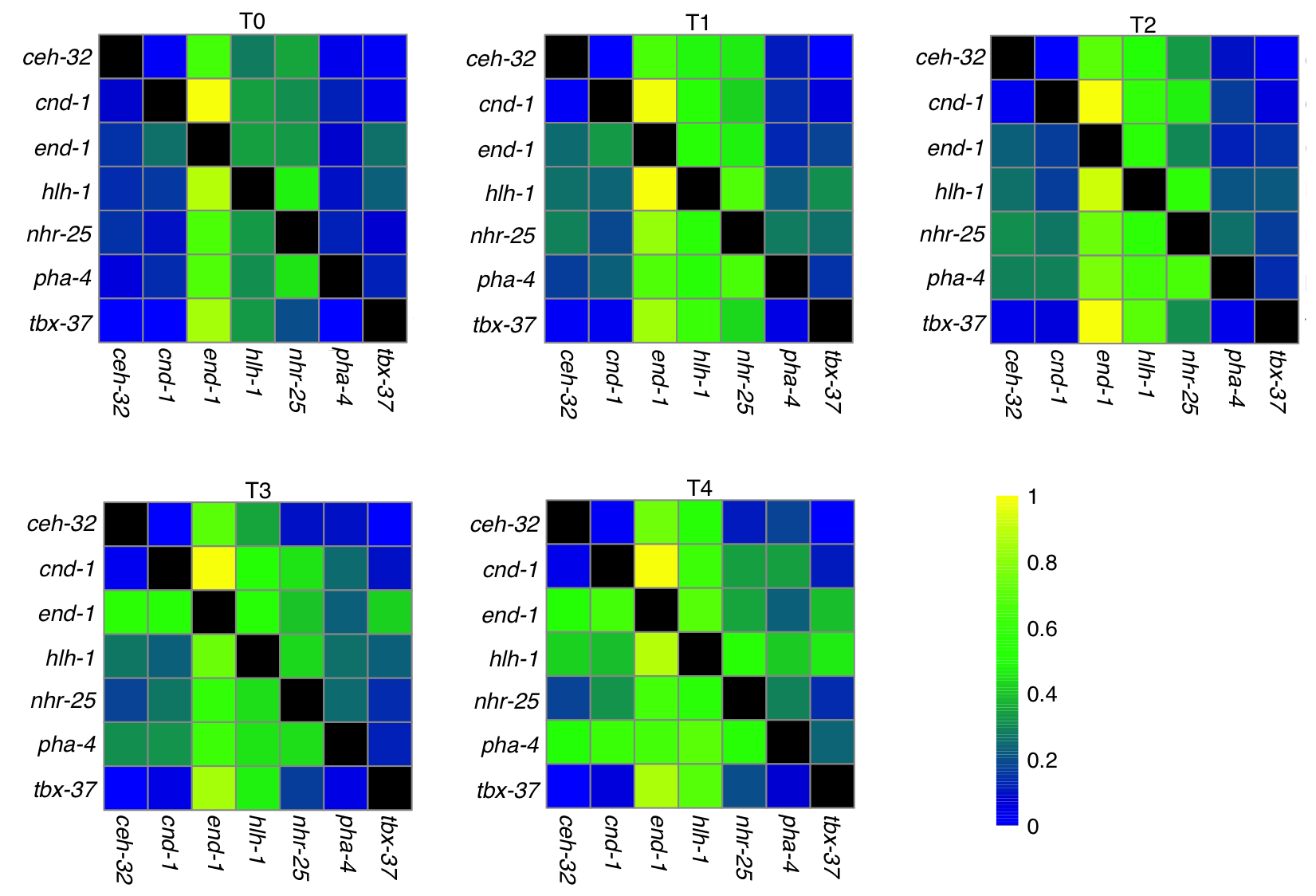


**Supplemental_Fig_S11.** Differentially expressed genes in pairwise comparisons between tissues.

Total numbers of differentially expressed genes were scaled to a max of 1 to compare the numbers of differentially expressed genes in pairwise comparisons between tissues at each of the five time points. Looking within a column, the value is the scaled number of differentially expressed genes higher in the sample on the x-axis, compared to the sample on the y-axis. For example, the third square from left in the top row of the T0 heatmap displays the scaled number of differentially expressed genes in *end-1* TO when compared with *ceh-32* T0. Conversely, within a row, the value is the scaled number of differentially expressed genes lower in the sample on the x-axis, compared to the sample on the y-axis.


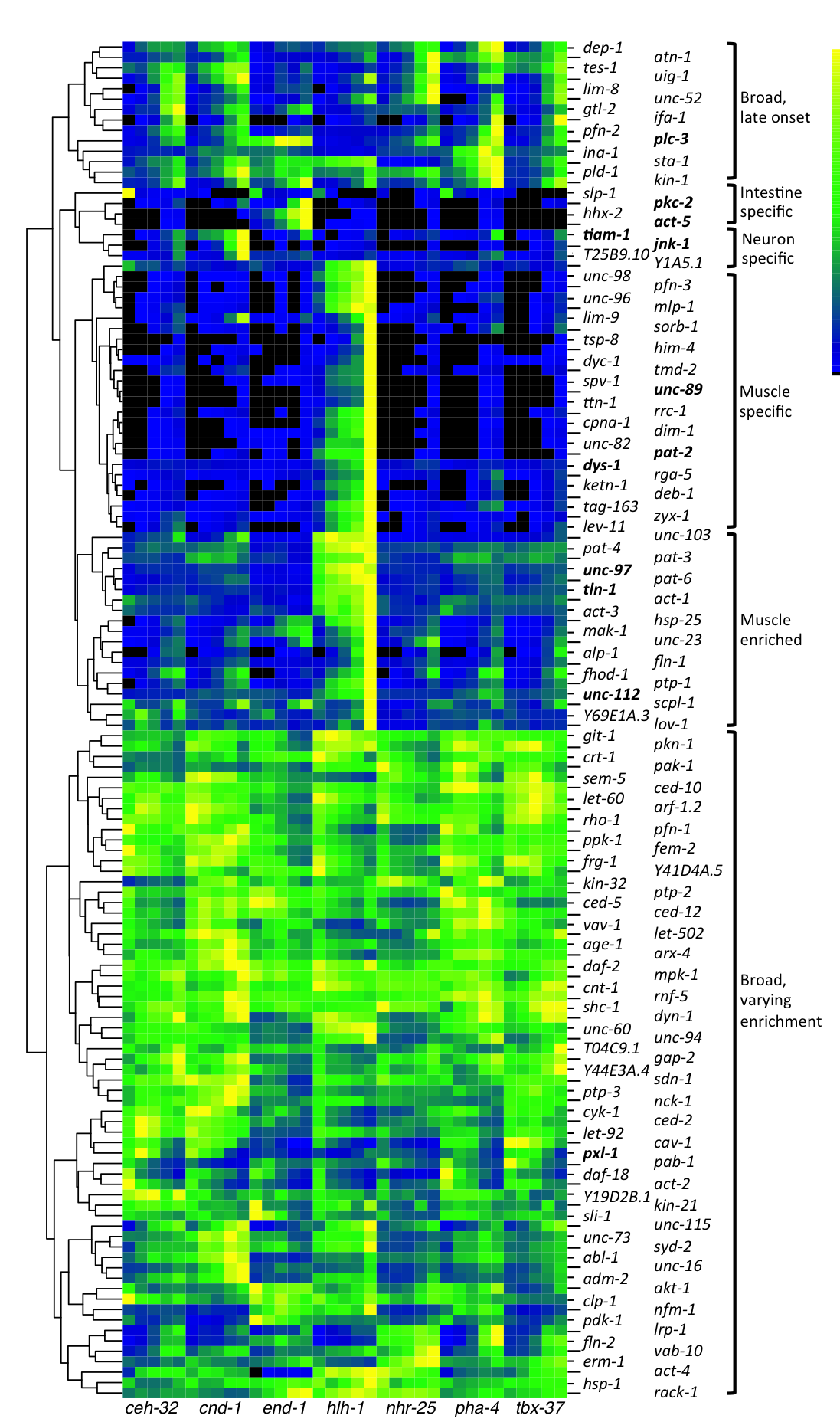


**Supplemental_Fig_S12.** Integrin adhesome genes clustered with similar gene expression patterns.

The genes that constitute the integrin adhesome have both broad and differential expression patterns. Some genes, such as the block of genes seen in this figure from *Y1A5.1* downwards to *unc-103* are highly specific to muscle cells, with a zygotic onset. Others such as those from *pat-4* down to *lov-1* are highly expressed in muscle cells, but have low to moderate expression in most tissues. Some genes are specific to non-muscle cell types such as *tiam-1,* *jnk-1,* and *T25B9.10* in neurons, and *pkc-2*, *hhx-2* and *act-5* in the intestine. Overall, about half of the genes in the integrin adhesome have broad expression amongst the cell types.

**Supplemental_Fig_S13**. Proportion of SL1 trans-splicing in operons.

In genes in operons with a small expression distance (similar expression in 35 dimensional space), the fraction of SL1 trans-splicing is relatively low. SL2 usage in downstream operon genes increases as a fraction of the total in gene pairs with a larger expression distance.

**Supplemental_Fig_S14.** Proportion of SL2 splicing in operons.

As gene expression of genes in operons increases, the ratio of SL2 reads to all splice leader reads decreases, with a higher proportion of SL1 usage in downstream genes.


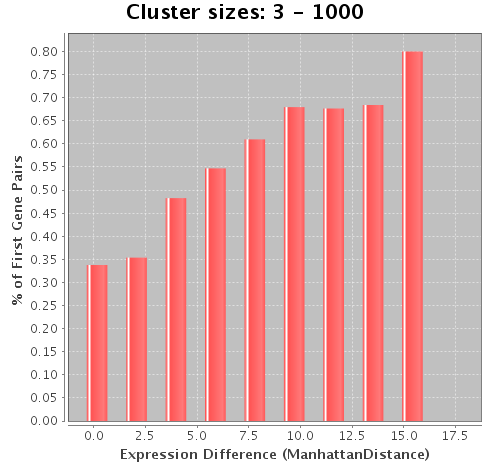


**Supplemental_Fig_S15.** Internal transcription factor binding sites in operons.

As the expression distance increases in 35 dimensional space between genes in operons (less similar gene expression patterns), there is a clear trend towards downstream genes having clusters of transcription factor binding sites in cluster sizes of 3 to 1,000.


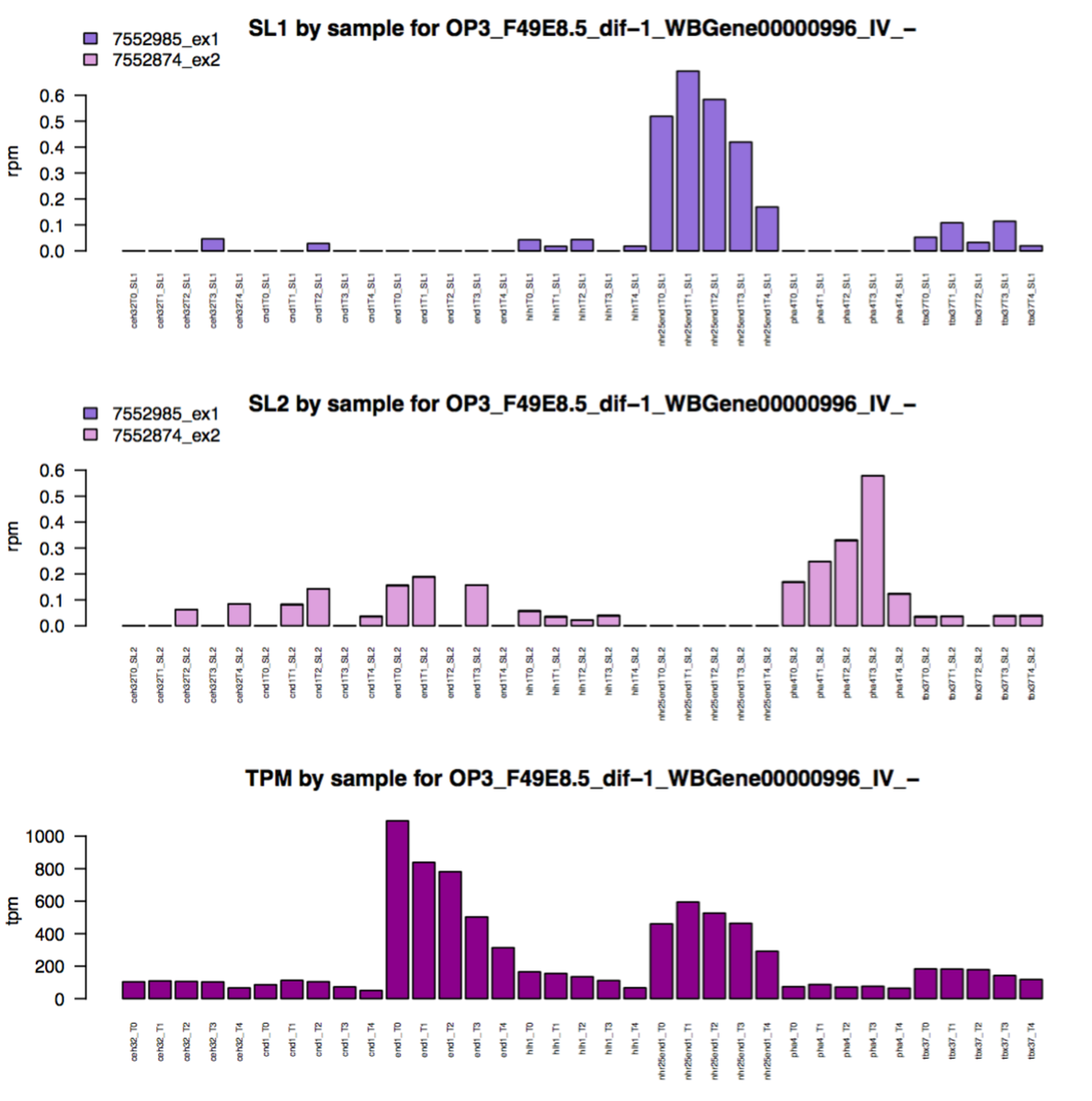


**Supplementary_Fig_S16.** Specific SL usage for *dif-1.*

In *dif-1*, which is reasonably expressed in all tissues/time points, SL1 is used in the hypodermis, whereas SL2 is used in the pharynx. The ex1 indicates one position is in the 1^st^ exon and the ex2 indicates the other position is in the 2^nd^ exon.


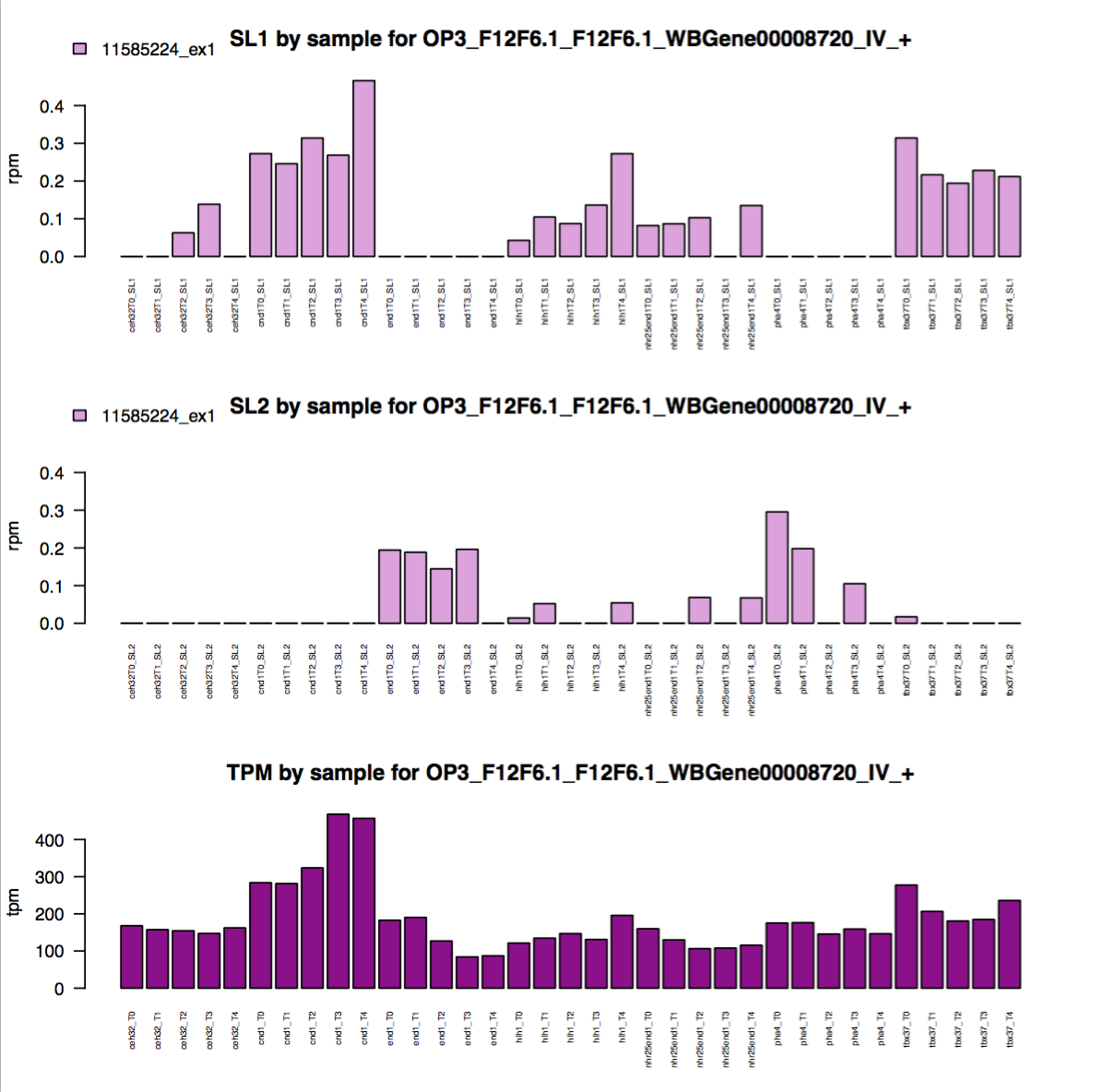


**Supplementary_Fig_S17**: Specific SL usage for *F12F6.1*.

In *F12F6.1,* which is reasonably expressed in all tissues/time points, SL1 is used in neurons, muscle, hypodermis, and the ABa lineage, while SL2 is used in the intestine, muscle, hypodermis, and pharynx.

**
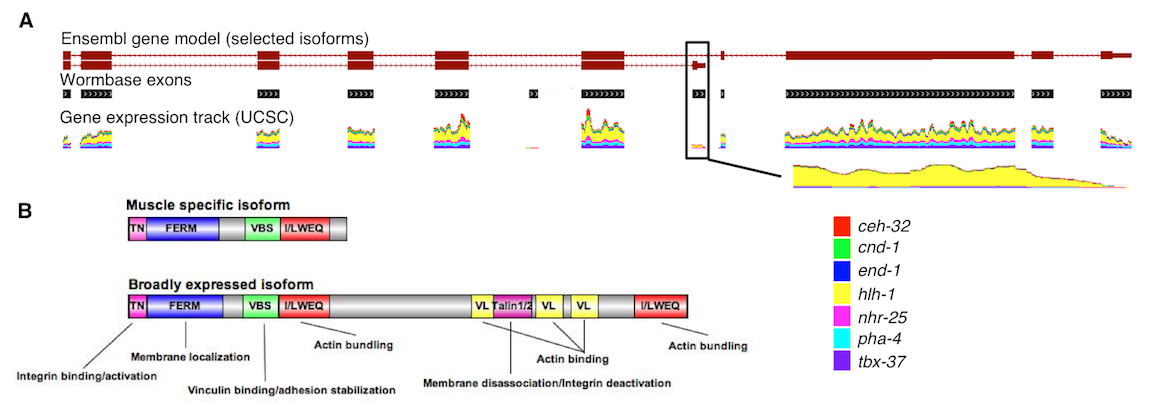
**

**Supplemental_Fig_S18.** Differential exon usage for *tln-1*

Most exons of *tln-1* have expression across all cell types, with greatest expression in the hlh-1 samples. Exon 8, however, has reads almost exclusively in muscle-derived samples (A). Gene models for *tln-1* show one of two isoforms that are predicted to use exon 8. When comparing the predicted protein structure of the longer, more broadly expressed *tln-1* isoform to the shorter muscle specific isoform, there are major differences in domain structure (B), with a number of key regulatory domains missing in the muscle specific isoform.


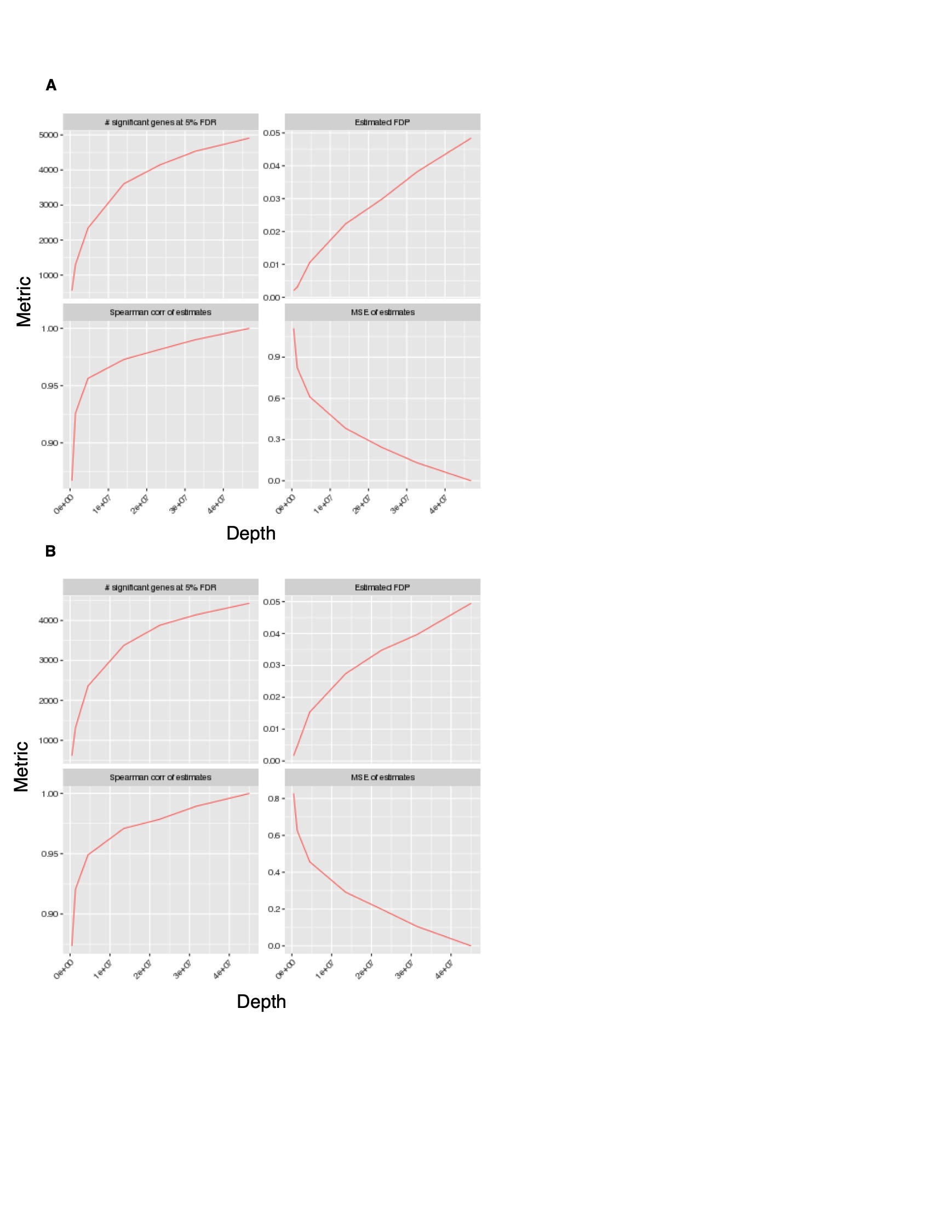


**Supplemental_Fig_S19.** Saturation of sequencing as tested by subSeq.

The program subSeq was used to subsample different read sequencing depths for gene expression differences using DESeq2. When comparing samples *end-1* T0 to *nhr-25* T0 (A), the differences in significant genes at FDR 5% is plateauing as sampled matrix size increases towards our total sequenced depth, indicating that we are nearing saturation of sequencing, but not oversaturated. A similar result is observed when comparing *end-1* T4 to *nhr-25* T4 (B). Spearman’s correlations also plateau close to 1 as matrix size increases. The false discovery proportion (FDP) is plotted in the upper rightmost quadrant of (A) and (B), and the mean squared error (MSE) is plotted in the lower rightmost quadrant.


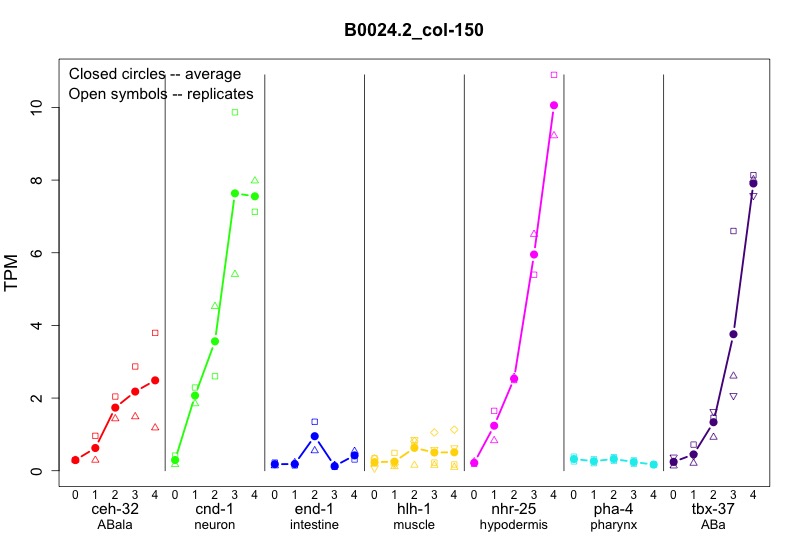


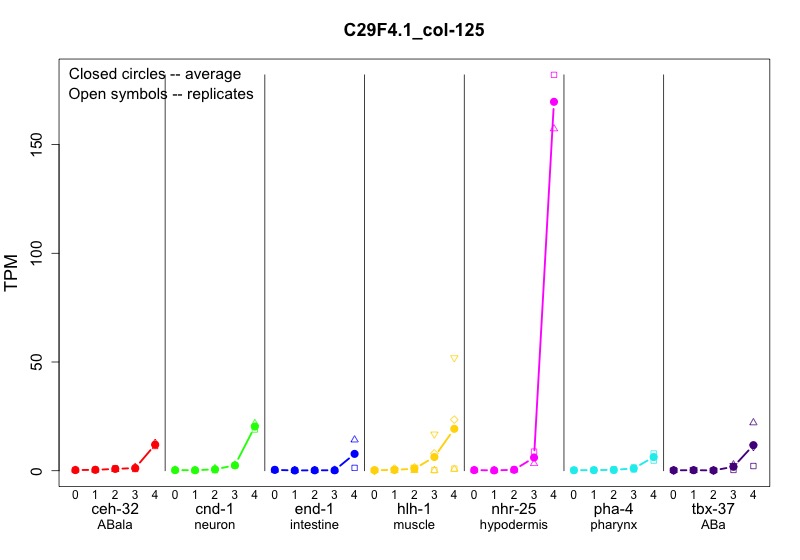


**Supplemental_Fig_S20.** Examples of expression profiles for *col-150* and *col-125*.

Expression profiles across tissues and time points for individual genes are available at <http://genome.sfu.ca/gexplore>. Individual data points as well as the averages are provided so the viewer can see the consistency of the data. Sections are labeled with both the gene used and the expected tissue.

**Supplemental_Table_S1:** Tissues excluded from analysis for DESeq2 comparisons

| **Tissue** | **Tissues to exclude** |
| --- | --- |
| *ceh-32* | *tbx-37, cnd-1* |
| *cnd-1* | *tbx-37, ceh-32* |
| *end-1* | none |
| *hlh-1* | none |
| *nhr-25* | none |
| *pha-4* | *tbx-37* |
| *tbx-37* | *cnd-1, ceh-32, pha-4* |

**Supplemental_Table_S8:** Differential usage of first exons

| **Tissue** | **Time** | **Lower exons** | **Lower junctions** | **Lower novels** | **Total genes** | **Higher exons** | **Higher junctions** | **Higher novels** | **Total** |
| --- | --- | --- | --- | --- | --- | --- | --- | --- | --- |
| ***cnd1*** | T0 | 2 | 3 | 0 | 4 | 31 | 19 | 0 | 5 |
| ***cnd1*** | T1 | 4 | 1 | 0 | 2 | 24 | 28 | 0 | 10 |
| ***cnd1*** | T2 | 0 | 1 | 0 | 1 | 26 | 14 | 0 | 26 |
| ***cnd1*** | T3 | 24 | 19 | 0 | 18 | 32 | 24 | 6 | 35 |
| ***cnd1*** | T4 | 31 | 25 | 0 | 32 | 39 | 22 | 8 | 45 |
| ***end1*** | T0 | 16 | 9 | 3 | 11 | 34 | 22 | 3 | 22 |
| ***end1*** | T1 | 15 | 5 | 3 | 8 | 17 | 7 | 0 | 17 |
| ***end1*** | T2 | 9 | 2 | 4 | 9 | 25 | 16 | 2 | 25 |
| ***end1*** | T3 | 28 | 29 | 7 | 29 | 60 | 40 | 7 | 59 |
| ***end1*** | T4 | 16 | 12 | 4 | 23 | 50 | 29 | 7 | 48 |
| ***hlh1*** | T0 | 9 | 1 | 0 | 6 | 32 | 32 | 7 | 26 |
| ***hlh1*** | T1 | 29 | 14 | 1 | 13 | 52 | 49 | 12 | 45 |
| ***hlh1*** | T2 | 28 | 19 | 2 | 20 | 73 | 63 | 12 | 52 |
| ***hlh1*** | T3 | 43 | 21 | 3 | 32 | 78 | 74 | 15 | 59 |
| ***hlh1*** | T4 | 77 | 48 | 6 | 50 | 68 | 75 | 20 | 57 |
| ***pha4*** | T0 | 6 | 3 | 0 | 2 | 2 | 1 | 1 | 3 |
| ***pha4*** | T1 | 8 | 5 | 0 | 3 | 3 | 0 | 1 | 2 |
| ***pha4*** | T2 | 11 | 3 | 0 | 6 | 12 | 3 | 0 | 5 |
| ***pha4*** | T3 | 9 | 8 | 0 | 7 | 24 | 21 | 6 | 19 |
| ***pha4*** | T4 | 26 | 13 | 1 | 20 | 34 | 28 | 8 | 31 |
| ***nhr25*** | T0 | 18 | 4 | 0 | 20 | 6 | 4 | 3 | 8 |
| ***nhr25*** | T1 | 41 | 4 | 0 | 33 | 6 | 2 | 1 | 6 |
| ***nhr25*** | T2 | 21 | 4 | 0 | 21 | 15 | 3 | 1 | 16 |
| ***nhr25*** | T3 | 6 | 2 | 0 | 4 | 9 | 5 | 2 | 10 |
| ***nhr25*** | T4 | 2 | 7 | 0 | 8 | 12 | 1 | 1 | 13 |
| ***tbx37*** | T0 | 4 | 7 | 0 | 3 | 24 | 11 | 2 | 6 |
| ***tbx37*** | T1 | 13 | 5 | 0 | 5 | 12 | 5 | 2 | 8 |
| ***tbx37*** | T2 | 7 | 3 | 0 | 5 | 9 | 5 | 1 | 6 |
| ***tbx37*** | T3 | 8 | 3 | 0 | 7 | 9 | 12 | 0 | 10 |
| ***tbx37*** | T4 | 13 | 4 | 0 | 10 | 16 | 10 | 5 | 21 |

**Supplemental_Table_S14:** Summary counts of tables Supplemental_Table_S12.txt and Supplemental_Table_S13.txt

|  | **SL1 to SL2** | **SL1 to SL1 or SL2 to SL2** | **SL1 to SL2** | **SL1 to SL1 or SL2 to SL2** | **SL1 to SL2** | **SL1 to SL1 or SL2 to SL2** |
| --- | --- | --- | --- | --- | --- | --- |
|  | **Different positions*** | **Different positions** | **Same position** | **Same position** | **total** | **total** |
| between tissues | 145 | 97 | 163 | 107 | 274 | 183 |
| between tissues confirmed by differential exon usage | 64 | 35 | 46 | 17 | 97 | 50 |
| within tissues | 59 | 50 | 56 | 50 | 103 | 89 |
| within tissues confirmed by differential exon usage | 11 | 11 | 6 | 2 | 13 | 13 |
| total genes | 150 | 104 | 171 | 113 | 283 | 191 |
| total genes with confirmed by differential exon usage | 66 | 38 | 49 | 18 | 100 | 54 |

*Different positions is defined as separated by >=20 bases

**Supplemental_Table_S15:** Putative RNA editing events

|  | **A-to-G edits with support for reference** | **A-to-G edits no support for reference** | **Non-A-to-G edits with support for reference** | **Non-A-to-G edits no support for reference** |
| --- | --- | --- | --- | --- |
| **pseudogene_exon** | 6 | 1 | 2 | 1 |
| **UTR** | 18 | 0 | 3 | 0 |
| **curated_exon** | 71 | 6 | 53 | 12 |
| **curated_intron** | 29 | 0 | 38 | 2 |
| **ncRNA_exon** | 26 | 0 | 22 | 1 |
| **transposon** | 7 | 0 | 1 | 0 |
